# Social dilemmas of sociality due to beneficial and costly contagion

**DOI:** 10.1371/journal.pcbi.1010670

**Published:** 2022-11-21

**Authors:** Daniel B. Cooney, Dylan H. Morris, Simon A. Levin, Daniel I. Rubenstein, Pawel Romanczuk

**Affiliations:** 1 Department of Mathematics, University of Pennsylvania, Philadelphia, Pennsylvania, United States of America; 2 Department of Ecology & Evolutionary Biology, University of California, Los Angeles, California, United States of America; 3 Department of Ecology & Evolutionary Biology, Princeton University, Princeton, New Jersey, United States of America; 4 Institute for Theoretical Biology, Department of Biology, Humboldt-Universität zu Berlin, Berlin, Germany; 5 Bernstein Center for Computational Neuroscience Berlin, Berlin, Germany; 6 Science of Intelligence, Research Cluster of Excellence, Berlin, Germany; University of Zurich, SWITZERLAND

## Abstract

Levels of sociality in nature vary widely. Some species are solitary; others live in family groups; some form complex multi-family societies. Increased levels of social interaction can allow for the spread of useful innovations and beneficial information, but can also facilitate the spread of harmful contagions, such as infectious diseases. It is natural to assume that these contagion processes shape the evolution of complex social systems, but an explicit account of the dynamics of sociality under selection pressure imposed by contagion remains elusive. We consider a model for the evolution of sociality strategies in the presence of both a beneficial and costly contagion. We study the dynamics of this model at three timescales: using a susceptible-infectious-susceptible (SIS) model to describe contagion spread for given sociality strategies, a replicator equation to study the changing fractions of two different levels of sociality, and an adaptive dynamics approach to study the long-time evolution of the population level of sociality. For a wide range of assumptions about the benefits and costs of infection, we identify a social dilemma: the evolutionarily-stable sociality strategy (ESS) is distinct from the collective optimum—the level of sociality that would be best for all individuals. In particular, the ESS level of social interaction is greater (respectively less) than the social optimum when the good contagion spreads more (respectively less) readily than the bad contagion. Our results shed light on how contagion shapes the evolution of social interaction, but reveals that evolution may not necessarily lead populations to social structures that are good for any or all.

## Author summary

Social interactions among individuals in animal groups provide a range of evolutionary benefits and risks. On the one hand, social contacts can promote learning and the adoption of innovations; on the other hand, such interactions can expose individuals to the harms of infectious disease. In this paper, we study the evolution of social gregariousness in the presence of both a beneficial and a costly contagion, which are jointly spreading in a population. Assuming that, all else equal, individuals prefer increased exposure to the good contagion and decreased exposure to the bad contagion, we characterize a socially-optimal level of gregariousness that best balances the relative exposure to the two contagions. However, using the mathematical frameworks of replicator equations and adaptive dynamics, we show that evolutionary competition between sociality strategies produces a social dilemma: individuals endeavoring to maximize their fitnesses drive the population to a level of gregariousness at which all individuals are worse off. In some cases, social behavior can disappear entirely—even when any level of gregariousness would be advantageous for the population as a whole. We also propose mechanisms to help overcome the social dilemma, showing how groups can help to establish more efficient levels of social interaction.

## Introduction

### The evolution of sociality

Social interaction has evolved many times [1]. Evolutionary explanations for social behavior cite its many benefits, such as the sharing of collective information. But social living has costs. For one, it can facilitate the spread of infectious diseases [2]. A basic premise of disease biology and modeling is that higher levels of contact among individuals facilitates disease spread [3]. But beneficial contagion is also possible, such as the adoption of useful innovations [4, 5] or the spread of social information [6]. Evolutionary tradeoffs between contagious benefits and contagious costs of social behavior have been hypothesized [6] and modeled theoretically for specific animal systems, such as bats [7], but a general, formal theory of how selection imposed by contagions shapes the rate social interaction itself is needed.

In this paper, we formulate a simple and flexible framework for modeling the evolution of sociality strategies given the benefits and costs of social transmission. We use an adaptive dynamics approach [8–10] to model the evolution of sociality in the presence of contagious benefits and costs. In this framework, epidemiology (contagion processes), competition (success or failure of different sociality strategies), and trait evolution (change in the overall population sociality level) occur on separate timescales: contagion is fastest, then competition, then trait evolution.

We find that the evolutionary consequences of beneficial and costly contagion for social behavior depend not only on the benefits and harms of the two contagions but also on their relative transmissibility. When the two contagions are not precisely equal in their transmissibilty, a social dilemma occurs. Individual-level selection on sociality drives the population to an equilibrium at which all individuals are worse off than they would be if they all could agree on some socially-optimal shared social interaction level: either with less access to the good contagion (e.g. less informed) or with more exposure to the bad (e.g. sicker). In some cases, beneficial sociality vanishes entirely: in an attempt to avoid disease, individuals reduce their interaction to the point that the beneficial contagion cannot spread at all. We explore ways in which this evolutionary problem—analogous to a prisoners’ dilemma—can be mitigated.

Our results reveal that evolutionary dynamics can produce social behavior, but that one should not assume that such behavior is optimal for the population—or even ideal for the individual. Rather, evolved levels of social interaction must be stable against evolutionary invasion—even if that means an outcome that is sub-optimal for all members of the social group.

### Existing related work

A substantial theoretical and empirical literature has addressed the evolution of sociality [1], but the role of contagion processes has received minimal theoretical attention, despite the fact that contagion is a near-ubiquitous property of social networks. It has often been proposed that animals may modulate their social interactions to avoid infectious diseases [6], but few models exist that study how this disease avoidance might trade off against the benefits of social contact.

The infectious disease literature includes adaptive behavior models, in which individuals change their behavior during epidemics to avoid [11–13] or seek out [14] the contagion. Similarly, Reluga [15] provides a game-theoretic treatment of disease avoidance in a two-population disease model. Other authors have modeled social contagion processes that modulate simultaneously-spreading infection processes [16–18]: examples include models of the simultaneous spread of an interaction strategy alongside an infectious disease [19–22], and of the spread of a social contagion of awareness or fear (and thus disease-avoiding behaviors) alongside an infectious epidemic [17, 23].

Much of this literature focuses on strategic or contagious behavior changes that occur on the same timescale as the disease outbreak. We are interested in the long-term evolution of social behavior itself. When the costs and benefits of contagion drive evolution, what are the consequences for levels of social behavior?

Recent work has explored the interplay between infectious disease and the behavioral evolution of social interactions [24], including models showing that division-of-labor can emerge in the presence of disease risk [25], that serial monogamy can arise under selection imposed by sexually-transmitted pathogens [26], and that fission-fusion dynamics in bat colonies can result from tradeoffs between information about roosting sites and risks from pathogen infection [7]. A common theme across these systems is the tradeoff between the benefits of social learning and the costs of infection [27], and past studies have also explored the coevolution of host sociality and pathogen virulence [28, 29]. Most relevant to the current paper is a model proposed by Ashby and Farine [29], who studied the evolution of social interaction rates in the presence of an informational contagion that conferred decreased mortality to an infectious disease spreading simultaneously in the population. The main contributions of our paper are (1) our formulation of a flexible framework for exploring the tradeoff between the costs of a bad contagion and the benefits of a good contagion, and (2) showing how the resulting evolution of social interaction rates may produce sub-optimal outcomes for the population.

### Structure of the paper

In this paper, we study the epidemiological and evolutionary dynamics of sociality strategies over three progressively longer timescales. We first consider a fast epidemiological timescale, and study how the good and bad contagion converge to either endemic or disease-free equilibria depending on the levels of social interaction in the population, including when two sociality strategies exist in fixed proportions in the population. Next, we consider how the utility of individuals obtained from steady-state levels of the good and bad contagion can influence the evolution of sociality strategies, using a replicator equation framework to see how the fraction of resident and mutant sociality strategies will evolve on an intermediate evolutionary timescale. Finally, we consider a slower evolutionary timescale in which the quantitative resident level of sociality can evolve, and employ the framework of adaptive dynamics to explore the long-run evolutionarily-stable sociality strategies. For both the replicator equation and adaptive dynamics frameworks, we consider the tension between individual and social utility optimization, asking how the levels of sociality chosen by self-interested individuals compare to the balance of good and bad contagion that would provided by a central planner.

The remainder of the paper is structured as follows. In the Model section, we introduce our model for the spread of good and bad contagions via social interaction, formulate a utility function balancing the benefits of the good contagion and the costs of the bad contagion, and calculate the socially-optimal level of sociality. In the Results section, we present our analysis of the evolutionary competition between sociality strategies, studying competition between pairs of strategies in “Replicator dynamics” and the long-time evolution of social interaction rate in “Adaptive dynamics”. In the Discussion section, we further explore the implications of the social dilemmas found in the evolutionary dynamics of sociality strategies, contextualizing our results in relation to existing work in the literature on evolutionary game theory, social evolution, and behavior-driven modeling in epidemiology.

In S1 Text, we provide detailed analysis of contagion and evolutionary dynamics for populations featuring two sociality strategies (Section A of S1 Text); extend our characterization of evolutionary stability and attempts to mitigate social dilemmas with assortative interaction rules (Section B of S1 Text), and present derivations for example linear and Cobb-Douglas utility functions from expected payoffs achieved by individuals at equilibrium states of the contagion dynamics (Section C of S1 Text).

## Model

When they interact with one another, individuals are potentially exposed to contagion. We consider two contagion processes: a beneficial (“good”) contagion process *g* and a harmful (“bad”) contagion process *b*. For example, the good contagion could be the spread of beneficial social information and the bad contagion could be the spread of a harmful infectious disease. We assume that each contagion is governed by Susceptible-Infectious-Susceptible (SIS) dynamics: individuals who are not currently infectious are susceptible (*S*), and can become infectious (*I*) through interactions with currently infectious individuals.

We call an individual’s infection status for each contagion their “state”. Four states are possible: susceptible to both good and bad, infectious with the good but not the bad, infectious with the bad but not the good, and infectious with both. We assume that the good and bad contagions spread entirely independently, so an individual’s probability of becoming infected with the bad contagion in a given social interaction does not depend on whether that individual is currently susceptible or infectious with the good contagion, and vice versa. This contrasts with situations such as contagious fear spreading alongside a pathogen, in which one’s state of fear may affect one’s probability of contracting the pathogen (as considered by Perra and coauthors [17] and by Epstein and coauthors [30, 31], for example).

Since the contagions spread independently, we can characterize population based upon the fractions susceptible to (*S*^(*g*)^) infectious with (*I*^(*g*)^) the good contagion, and the corresponding fractions *S*^(*b*)^ and *I*^(*b*)^ for the bad contagion. We assume that individuals have social interactions with rate *σ*, and that this rate of social interaction is fixed throughout the course of the contagion dynamics. This allows us to consider contagion dynamics that occur on a faster timescale than the evolutionary dynamics governing the strategies for social interaction. For the good and bad contagions, we assume that an interaction between a susceptible and infectious individual results in transmission of the contagion with probability *p*_*g*_ and *p*_*b*_, respectively. For each contagion *x* ∈ {*g*, *b*}, susceptible individuals *S*^(*x*)^ therefore become infected at a rate *σp*_*x*_*I*^(*x*)^, and we further assume that infected individuals spontaneously recover and return to the susceptible state at rate *γ*_*x*_. The contagion dynamics thus obey the following ordinary differential equations:

$$\begin{array}{c} \frac{dS^{\left(g\right)}}{dt}=-\sigma p_{g}S^{\left(g\right)}I^{\left(g\right)}+\gamma_{g}I^{\left(g\right)} \end{array}$$ (1a)

$$\begin{array}{c} \frac{dI^{\left(g\right)}}{dt}=\sigma p_{g}S^{\left(g\right)}I^{\left(g\right)}-\gamma_{g}I^{\left(g\right)} \end{array}$$ (1b)

$$\begin{array}{c} \frac{dS^{\left(b\right)}}{dt}=-\sigma p_{b}S^{\left(b\right)}I^{\left(b\right)}+\gamma_{b}I^{\left(b\right)} \end{array}$$ (1c)

$$\begin{array}{c} \frac{dI^{\left(b\right)}}{dt}=\sigma p_{b}S^{\left(b\right)}I^{\left(b\right)}-\gamma_{b}I^{\left(b\right)} \end{array}$$ (1d)

The two contagion processes have basic reproduction numbers

$$\begin{array}{c} R^{\left(g\right)}=\frac{\sigma p_{g}}{\gamma_{g}}\;\;\text{and}\;\;R^{\left(b\right)}=\frac{\sigma p_{b}}{\gamma_{b}}. \end{array}$$ (2)

We can rearrange Eq (2) to obtain the following relationship between $R^{\left(b\right)}$ and $R^{\left(g\right)}$:

$$\begin{array}{c} R^{\left(b\right)}=cR^{\left(g\right)},\;\;\text{where}\;\;c=\frac{p_{b}\gamma_{g}}{p_{g}\gamma_{b}}. \end{array}$$ (3)

The parameter *c* can be thought of as the relative transmissability of the bad contagion compared to the good. Using Eqs (2) and (3), we can describe the impact of the social contact rate *σ* on the dynamics of the good and bad contagion through the parameters $R^{\left(g\right)}$ and *c*. For simplicity, we will now primarily characterize the sociality strategies of individuals through the resulting reproduction number $R^{\left(g\right)}$. We use this parametrization of the contagion dynamics with the right-hand side expressed in terms of *c* and $R^{\left(g\right)}$ in order to have a compact representation of how the spread of the coupled contagions depend social interaction rates (encoded by $R^{\left(g\right)}=\frac{\sigma p_{g}}{\gamma_{g}}$) and the relative infectiousness of the good and bad contagion (as encoded by *c*). Notably, derivatives of the social utility and invasion fitness with respect to $R^{\left(g\right)}$ will be proportional to the derivatives of the same functions with respect to *σ*, and so social optima and evolutionarily stable strategies expressed in terms of the reproduction number $R^{\left(g\right)}$ will directly correspond to equivalent quantities expressed in terms of the social interaction rate *σ*.

After applying the formula of Eqs (2) and (3) to Eq (1), we can divide through by the recovery rates *γ*_*g*_ and *γ*_*b*_ and use the fact that *I*^(*g*)^+ *S*^(*g*)^ = 1 and *I*^(*b*)^ + *S*^(*b*)^ = 1 to express the dynamics of the good and bad contagion through the pair of decoupled differential equations:

$$\begin{array}{c} \frac{1}{\gamma_{g}}\frac{dI^{\left(g\right)}}{dt}=R^{\left(g\right)}\left(1-I^{\left(g\right)}\right)I^{\left(g\right)}-I^{\left(g\right)} \end{array}$$ (4a)

$$\begin{array}{c} \frac{1}{\gamma_{b}}\frac{dI^{\left(b\right)}}{dt}=cR^{\left(g\right)}\left(1-I^{\left(b\right)}\right)I^{\left(b\right)}-I^{\left(b\right)}. \end{array}$$ (4b)

Eq (4) shows that, for a given value of $R^{\left(g\right)}\geq 0$, the good and the bad contagions have globally-stable equilibria ${\hat{I}}^{\left(g\right)}$ and ${\hat{I}}^{\left(b\right)}$ given by

$$\begin{array}{c} {\hat{I}}^{\left(g\right)}\left(R^{\left(g\right)}\right)=\{\begin{array}{cc} 1-\frac{1}{R^{\left(g\right)}} & R^{\left(g\right)}>1\\ 0 & R^{\left(g\right)}\leq 1 \end{array} \end{array}$$ (5a)

$$\begin{array}{c} {\hat{I}}^{b}\left(R^{\left(g\right)}\right)=\{\begin{array}{cc} 1-\frac{1}{cR^{\left(g\right)}} & cR^{\left(g\right)}>1\\ 0 & cR^{\left(g\right)}\leq 1 \end{array} \end{array}$$ (5b)

**Fig 1:**
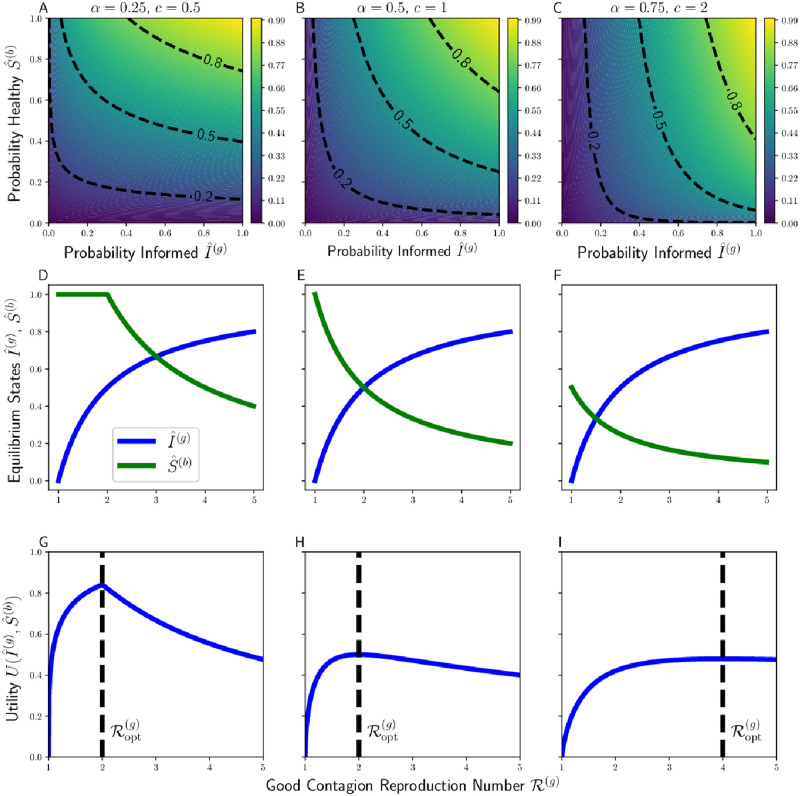
Example heatmaps of Cobb-Douglas utility for various weights *α* of emphasis on acquiring the good contagion versus avoiding the bad contagion (**A–C**), endemic equilibria ${\hat{I}}^{\left(g\right)}$ and ${\hat{S}}^{\left(b\right)}$ as a function of sociality strategy $R^{\left(g\right)}$ for different values of relative transmissibility *c* (**D–F**), and resultant overall utilities as a function of $R^{\left(g\right)}$ given *α* and *c* (**G–I**). Note that in certain cases (e.g. **G**) utility is maximized by setting $R^{\left(g\right)}=\frac{1}{c}$, the maximal degree of sociality at which the bad contagion fails to spread. Vertical dashed lines in **G–I** correspond to the socially-optimal sociality strategy $R_{\text{opt}}^{\left(g\right)}$.

For examples of how these equilibria vary with $R^{\left(g\right)}$ and *c*, see Fig 1D, 1E and 1F.

### Benefits and costs of sociality

To understand how sociality evolves given the benefits of the good contagion and the costs of the bad contagion, we quantify those benefits and costs with a utility or fitness function, *U*. We assume that an individual’s utility depends on the equilibrium prevalences of the two contagions (Eq (4)), so we have $U({\hat{I}}^{\left(g\right)},{\hat{S}}^{\left(b\right)})$.

One interpretation of the equilibrium fractions infected ${\hat{I}}^{\left(g\right)}$ and ${\hat{S}}^{\left(b\right)}$ is as each individual’s long-run fraction of time spent infected with the good contagion and susceptible to the bad contagion, respectively. We also show (see Section C of S1 Text) that some linear and Cobb-Douglas utility functions can be derived by calculating an individual’s expected utility at the endemic equilibrium, where ${\hat{I}}^{\left(g\right)}$ and ${\hat{S}}^{\left(b\right)}$ are treated as the respective probabilities of being infected with the good contagion and susceptible to the bad contagion.

Implicit in this choice of utility functions is the assumption that the spreading processes for the good and bad contagions are fast relative to the rates of reproduction or social learning in the population (which can lead to changes in sociality *σ*). That is, we assume that contagion dynamics happen on a faster timescale than do births and deaths, and ignore demographic effects in our coupled SIS contagion models. To weigh the benefits of the good contagion against the costs of the bad contagion, we would like a utility function that increases the more time one spends infected with the good contagion (${\hat{I}}^{\left(g\right)}$) and also increases the more time one remains uninfected (i.e. susceptible) with the bad contagion (${\hat{S}}^{\left(b\right)}$).

### Cobb-Douglas utility functions

To capture this key property of always preferring greater ${\hat{I}}^{\left(g\right)}$ and ${\hat{S}}^{\left(b\right)}$, we explore as a first example the Cobb-Douglas family of utility functions:

$$\begin{array}{c} U\left({\hat{I}}^{\left(g\right)},{\hat{S}}^{\left(b\right)}\right)={\left({\hat{I}}^{\left(g\right)}\right)}^{\alpha }{\left({\hat{S}}^{\left(b\right)}\right)}^{1-\alpha }, \end{array}$$ (6)

where the parameter *α* ∈ [0, 1] measures the importance placed upon seeking out the benefits of the good contagion relative to avoiding the harms of the bad (Fig 1A, 1B and 1C).

This *α* allows us to explore how our evolutionary dynamics depend upon the relative importance of the good and bad contagions for utility or fitness, and how this relative importance interacts with their relative transmissibility *c* in shaping desirable and evolutionarily-stable rates of social interaction. The Cobb-Douglas utility functions allow us to compute explicit socially-optimal and evolutionarily-stable levels of sociality, but we show in Adaptive dynamics that many of our qualitative results generalize to broader classes of utility functions with similar properties.

Noting from Eq (5) that both ${\hat{I}}^{\left(g\right)}$ and ${\hat{S}}^{\left(b\right)}$ are functions of $R^{\left(g\right)}$, we can write *U* as a function of $R^{\left(g\right)}$. Plugging in the equilibria from Eq (5), we write the Cobb-Douglas utility $U(R^{\left(g\right)}$) as:

$$\begin{array}{c} U\left(R^{\left(g\right)}\right)=\{\begin{array}{cc} 0 & :R^{\left(g\right)}\leq 1\\ {\left(1-\frac{1}{R^{\left(g\right)}}\right)}^{\alpha } & :1\leq R^{\left(g\right)}\leq \frac{1}{c}\\ {\left(1-\frac{1}{R^{\left(g\right)}}\right)}^{\alpha }{\left(\frac{1}{cR^{\left(g\right)}}\right)}^{1-\alpha } & :R^{\left(g\right)}\geq \frac{1}{c} \end{array} \end{array}$$ (7)

These three cases come from the fact that the good contagion will not spread if $R^{\left(g\right)}\leq 1$, the bad contagion will not spread if $R^{\left(b\right)}=cR^{\left(g\right)}\leq 1$, and otherwise both spread. Notably, the middle case in which $1\leq R^{\left(g\right)}\leq \frac{1}{c}$ cannot occur when *c* > 1. When *c* > 1, the bad contagion spreads more readily than the good contagion, so the bad contagion will always be present at positive endemic equilibrium when the good contagion survives in the long-time limit (provided both contagions are present in the initial population).

We also note that $U(R^{\left(g\right)})\geq 0$ for $R^{\left(g\right)}\geq 1$, and that utility is minimized when $R^{\left(g\right)}\leq 1$ and correspondingly $U(R^{\left(g\right)})=0$.

### Socially-optimal levels of sociality

For a population with monomorphic sociality (i.e. one $R^{\left(g\right)}$ shared by all individuals), there exists a socially-optimal $R^{\left(g\right)}$ that maximizes each individual’s fitness (Fig 1G, 1H and 1I).

Using the piecewise characterization of the Cobb-Douglas utility function from Eq (7), We can calculate that the reproduction $R_{\text{opt}}^{\left(g\right)}$ maximizing the social utility $U(R^{\left(g\right)})$ is given by

$$\begin{array}{c} R_{\text{opt}}^{\left(g\right)}=\;\text{max}\;(\frac{1}{c},\;\frac{1}{1-\alpha }). \end{array}$$ (8)

For completeness, we present the derivation of this socially optimal level of sociality $R_{\text{opt}}^{\left(g\right)}$ in Section B.1 of S1 Text.

From Eq (8), we see that $R_{\text{opt}}^{\left(g\right)}>1$ provided that *α* > 0, so the social optimum under the Cobb-Douglas utility features involves socializing enough to achieve good contagion transmissions whenever individual utility places any weight on the good contagion. Furthermore, because the bad contagion cannot spread when $R^{\left(b\right)}=cR^{\left(g\right)}\leq 1$, the bad contagion will be absent from the population when $R^{\left(g\right)}\leq \frac{1}{c}$. As a result, we see from Eq (8) that the socially optimal interaction rate features elimination of the bad contagion when *α* ≤ 1 − *c*, which corresponds to the case in which the bad contagion spreads less readily than the good contagion and social utility places a sufficiently small relative weight upon acquiring the good contagion. By contrast, when *α* > 1 − *c*, the socially optimal level of sociality $R_{\text{opt}}^{\left(g\right)}$ will allow for the long-time survival of both the good and bad contagion.

We further illustrate our results in Fig 1, showing the Cobb-Douglas utility, the endemic equilibria, and the socially-optimal level of sociality for various *c* and *α*.

## Results

### Replicator dynamics

Having established the socially-optimal rate of social interaction for a monomorphic population, we now consider which sociality levels (“strategies”) can succeed under evolutionary competition, if individual utility or reproductive fitness depends on infection with the good and bad contagions.

We can consider a scenario in which individuals reproduce proportional to their utility, so that the proportion of individuals with sociality strategies leading to higher utility will increase over time. Alternatively, we could consider individuals who engage in social learning, imitating the sociality strategies of peers who are obtaining higher utilities. In either case, the fraction *f* of individuals following one strategy may change over time.

To analyze this competition, we first study pairwise competition between two sociality strategies *m* and *r*. First, we introduce our models of good and bad contagion dynamics in the presence of two sociality strategies (Contagion dynamics for two levels of sociality), and derive equilibrium contagion levels. Next, we use these equilibria and our utility functions to study how the fractions of the population following each strategy will evolve over time under a replicator equation modeling evolutionary competition (Competition between two sociality strategies).

#### Contagion dynamics for two levels of sociality

We assume that, on the epidemic time scale, there is a fixed fraction *f* of individuals following a strategy of interest (“mutant strategy”) *m* with social interaction rate *σ*_*m*_. The remaining fraction 1 − *f* of individuals follow a different strategy (“resident strategy”) *r* with interaction rate *σ*_*r*_. For both the mutant and resident strategies, we assume that the social interaction rates *σ*_*m*_ and *σ*_*r*_ are fixed throughout the course of the contagion dynamics. As in the monomorphic case, we can describe good contagion reproduction numbers $R_{m}^{\left(g\right)}=\frac{\sigma_{m}p_{g}}{\gamma_{g}}$ and $R_{r}^{\left(g\right)}=\frac{\sigma_{r}p_{g}}{\gamma_{g}}$ for the resident and mutant strategies, and resultant bad contagion reproduction numbers $R_{m}^{\left(b\right)}=cR_{m}^{\left(g\right)}$ and $R_{r}^{\left(b\right)}=cR_{r}^{\left(g\right)}$. As in Model, we use these relations to parametrize the resident and mutant sociality strategies via their respective reproduction numbers under the good contagion $R_{r}^{\left(g\right)}$ and $R_{m}^{\left(g\right)}$. Under the assumption that the probability of social interactions with individuals following the resident and mutant strategies is derived from unbiased sampling of the pool of available contacts, we show in Section A of S1 Text that the level of good contagion in the resident and mutant populations evolves according to

$$\begin{array}{c} \frac{1}{\gamma_{g}}\frac{dI_{r}^{\left(g\right)}}{dt}=R_{r}^{\left(g\right)}[\frac{R_{r}^{\left(g\right)}\left(1-f\right)I_{r}^{\left(g\right)}+R_{m}^{\left(g\right)}fI_{m}^{\left(g\right)}}{R_{r}^{\left(g\right)}\left(1-f\right)+R_{m}^{\left(g\right)}f}](1-I_{r}^{\left(g\right)})-I_{r}^{\left(g\right)} \end{array}$$ (9a)

$$\begin{array}{c} \frac{1}{\gamma_{g}}\frac{dI_{m}^{\left(g\right)}}{dt}=R_{m}^{\left(g\right)}[\frac{R_{r}^{\left(g\right)}\left(1-f\right)I_{r}^{\left(g\right)}+R_{m}^{\left(g\right)}fI_{m}^{\left(g\right)}}{R_{r}^{\left(g\right)}\left(1-f\right)+R_{m}^{\left(g\right)}f}](1-I_{m}^{\left(g\right)})-I_{m}^{\left(g\right)}, \end{array}$$ (9b)

with the corresponding system of ODEs holding for the bad contagion

$$\begin{array}{c} \frac{1}{\gamma_{b}}\frac{dI_{r}^{\left(b\right)}}{dt}=cR_{r}^{\left(g\right)}[\frac{R_{r}^{\left(g\right)}\left(1-f\right)I_{r}^{\left(b\right)}+R_{m}^{\left(g\right)}fI_{m}^{\left(b\right)}}{R_{r}^{\left(g\right)}\left(1-f\right)+R_{m}^{\left(g\right)}f}](1-I_{r}^{\left(b\right)})-I_{r}^{\left(b\right)} \end{array}$$ (9c)

$$\begin{array}{c} \frac{1}{\gamma_{b}}\frac{dI_{m}^{\left(b\right)}}{dt}=cR_{m}^{\left(g\right)}[\frac{R_{r}^{\left(g\right)}\left(1-f\right)I_{r}^{\left(b\right)}+R_{m}^{\left(g\right)}fI_{m}^{\left(b\right)}}{R_{r}^{\left(g\right)}\left(1-f\right)+R_{m}^{\left(g\right)}f}](1-I_{m}^{\left(b\right)})-I_{m}^{\left(b\right)}. \end{array}$$ (9d)

In particular, the terms in square brackets in Eq (9) describe the probability that a given social contact takes place with an infectious individual, and we note that this probability depends on the relative chance of interacting with individuals following the resident and mutant strategy due to both the relative abundance of these strategies and the relative social contact rates characterized by the strategies.

We show (see Section A of S1 Text) that the overall good contagion dynamics for the resident and mutant populations combined has a basic reproduction number given by

$$\begin{array}{c} R_{\text{net}}^{\left(g\right)}=\frac{f{\left(R_{m}^{\left(g\right)}\right)}^{2}+\left(1-f\right){\left(R_{r}^{\left(g\right)}\right)}^{2}}{fR_{m}^{\left(g\right)}+\left(1-f\right)R_{r}^{\left(g\right)}}. \end{array}$$ (10)

It follows from a result of Hethcote and Yorke on two population SIS dynamics that the disease-free equilibrium $\left({\hat{I}}_{m}^{\left(g\right)},{\hat{I}}_{r}^{\left(g\right)}\right)=\left(0,0\right)$ of Eqs (9a) and (9b) is globally stable when $R_{\text{net}}^{\left(g\right)}<1$, whereas, when $R_{\text{net}}^{\left(g\right)}>1$, there exists a unique endemic equilibrium $\left({\hat{I}}_{m}^{\left(g\right)},{\hat{I}}_{r}^{\left(g\right)}\right)\neq \left(0,0\right)$ that is the achieved in the long-time limit for any initial condition starting with any presence of the good contagion in the population (i.e. for any initial state other than the disease-free equilibrium (0, 0)). An analogous result holds for the equilibria of the bad contagion dynamics with $R_{\text{net}}^{\left(b\right)}=cR_{\text{net}}^{\left(g\right)}$.

#### Competition between two sociality strategies

Since we assume contagion dynamics occur on a much faster timescale than evolutionary competition, we can explore the evolutionary consequences of fitness/utility that depend on equilibrium fractions susceptible to the bad contagion ${\hat{S}}_{\cdot }^{\left(b\right)}\left(R_{m}^{\left(g\right)},R_{r}^{\left(g\right)},f\right)$ and infected with the good contagion ${\hat{I}}_{\cdot }^{\left(g\right)}\left(R_{m}^{\left(g\right)},R_{r}^{\left(g\right)},f\right)$.

For a given mutant fraction *f*, we define utility functions *U*_*m*_(*f*) and *U*_*r*_(*f*) for mutant and resident individuals, respectively:

$$\begin{array}{c} U_{m}\left(f\right)=U({\hat{I}}_{m}^{\left(g\right)}\left(R_{m}^{\left(g\right)},R_{r}^{\left(g\right)},f\right),{\hat{S}}_{m}^{\left(b\right)}\left(R_{m}^{\left(g\right)},R_{r}^{\left(g\right)},f\right))\\ U_{r}\left(f\right)=U({\hat{I}}_{r}^{\left(g\right)}\left(R_{m}^{\left(g\right)},R_{r}^{\left(g\right)},f\right),{\hat{S}}_{r}^{b}\left(R_{m}^{\left(g\right)},R_{r}^{\left(g\right)},f\right)). \end{array}$$ (11)

To encode our assumption that an individual’s chance of being reproducing or being imitated is proportional to its utility, we model *f*(*t*) according to the following replicator equation:

$$\begin{array}{c} \frac{df}{dt}=f\left(1-f\right)[U_{m}\left(f\right)-U_{r}\left(f\right)] \end{array}$$ (12)

This replicator equation can be derived from a variety of individual-based models of social imitation [32] as well as from models of reproduction-based natural selection [33].

Eq 12 has three types of biologically plausible equilibria: $\bar{f}=0$, $\bar{f}=1$ (population takeover by the resident or mutant, respectively), and interior frequencies $\bar{f}\in \left(0,1\right)$ where $U_{m}\left(\bar{f}\right)=U_{r}\left(\bar{f}\right)$ (coexistence of the two strategies).

**Fig 2:**
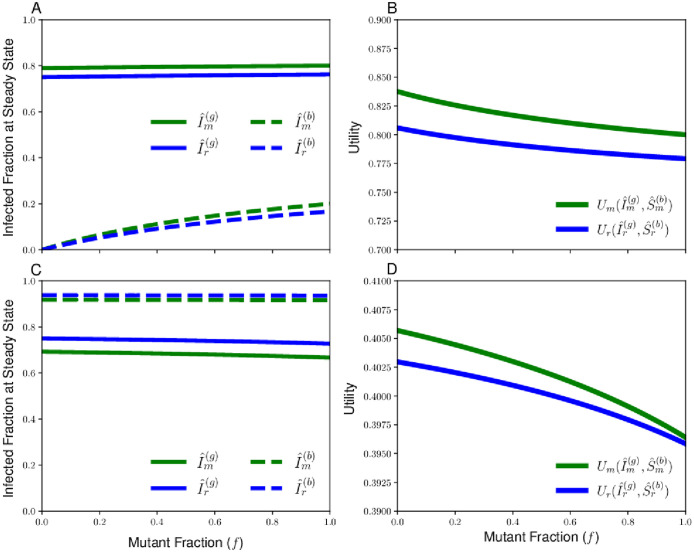
Sample contagion equilibria and Cobb-Douglas utility achieved by resident and mutant strategies as a function of the fraction of individuals following the mutant strategy *f*. We consider a resident with reproduction number $R_{r}^{\left(g\right)}=R_{\text{opt}}^{\left(g\right)}=4$ in all panels. (**A**,**C**): Endemic equilibria of the good and bad contagion for the cases of relative infectiousness and mutant reproduction number *c* = 0.25, $R_{m}^{\left(g\right)}=5$ (**A**) and *c* = 4, $R_{m}^{\left(g\right)}=3$ (**C**). (**B**,**D**): Plots of Cobb-Douglas utility *U*_*r*_(*f*) and *U*_*m*_(*f*) achieved at contagion equilibrium for individuals following resident and mutant strategy, will parameters *c* = 0.25, $R_{m}^{\left(g\right)}=5$ (**B**) or *c* = 4, $R_{m}^{\left(g\right)}=3$ (**D**). For both cases, we use a Cobb-Douglas utility function with weight parameter *α* = 0.75. In both cases, the utility of the mutant type (green curve) always exceeds the utility of the resident type (blue curve), and the replicator equation will favor fixation to an all-mutant composition.

We now use this replicator equation to study the dynamics of pairwise competition between two sociality strategies. In Fig 2, we display the endemic equilibria of the two-type contagion dynamics and the Cobb-Douglas utilities at the contagion equilibrium for both the socially-optimal level of sociality and a chosen mutant strategy across the possible compositions of *f* mutant and 1 − *f* resident sociality strategists. We consider an optimal sociality strategy $R_{\text{opt}}^{\left(g\right)}=4$, and choose a mutant strategy $R_{m}^{\left(g\right)}=7$ when the good contagion spreads more readily (Fig 2B, *c* = 0.25) and a mutant strategy $R_{m}^{\left(g\right)}=3$ when the bad contagion spreads more readily (Fig 2D, *c* = 4). In both cases, we see that *U*_*m*_(*f*) > *U*_*r*_(*f*) for all *f* ∈ [0, 1], so it follows from Eq (12) that the mutant proportion will always increase for the pairs of $R_{m}^{\left(g\right)}$ and $R_{r}^{\left(g\right)}$ we consider under replicator dynamics. Therefore, these choices of mutant strategies are able to successfully invade populations otherwise composed of the socially-optimal strategy, and will eventually fix to the composition *f* = 1. As a result, we see that strategies that maximize collective utility can lose out in evolutionary competition against rival strategies, showing that dynamics of Eq (12) produce a social dilemma in the evolution of sociality strategies.

**Fig 3:**
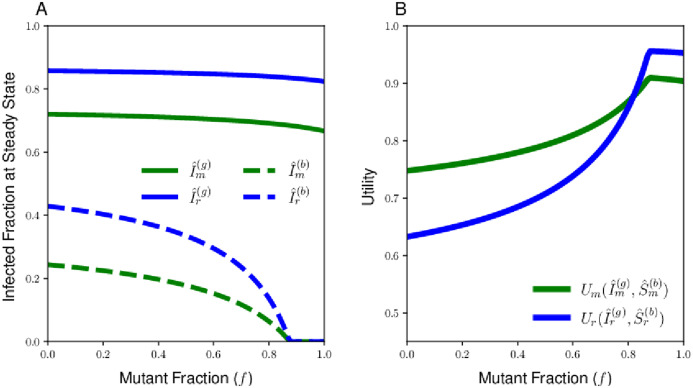
Endemic equilibria of the two contagions for the resident and mutant populations (A) and the Cobb-Douglas utility (B) for a case in which the resident and mutant sociality stategies lie on opposite sides of the social optimum. The utility for the resident strategy (blue curve) and the mutant strategy (green curve) intersect at a single fraction of mutants *f*, which is the equilibrium of the replicator equation at which the two types will coexist. The Cobb-Douglas utility has weight parameter *α* = 0.25, the relative infectiousness of the bad contagion is *c* = 0.25, and the resident and mutant reproduction numbers are given by $R_{r}^{\left(g\right)}=7$ and $R_{m}^{\left(g\right)}=3$, respectively.

In addition to the cases of pairwise dominance between strategies seen in Fig 2, we now demonstrate that there are pairs of sociality strategies for which stable coexistence can be achieved at an interior equilibrium under the replicator equation. In Fig 3, we provide an example of mutant and resident sociality strategies for which there is a fraction of mutants *f*_*eq*_ around 0.6 such that the mutant and resident have equal utilities *U*_*m*_(*f*_*eq*_) = *U*_*r*_(*f*_*eq*_). Because the mutants obtain higher utility than the residents for *f* < *f*_*eq*_ and the residents outcompete the mutants for *f* > *f*_*eq*_, we see that *f*_*eq*_ will be a stable interior steady state for the replicator equation, and a population with initial composition featuring a mix of mutants and residents will result in long-time coexistence of the two types composed of *f*_*eq*_ fraction mutants and 1 − *f*_*eq*_ fraction residents.

Having seen different possible behaviors under the replicator dynamics through Figs 2 and 3, we now turn to an analytical characterization of the possible scenarios for pairwise competition in the case of Cobb-Douglas utility. In Proposition 1, we show that there are three possible long-time behaviors—dominance of the mutant strategy, dominance of the resident strategy, and long-time coexistence of the resident and mutant strategies at a unique interior equilibrium– and that we can characterize which of these behaviors occurs by comparing the relative utility of each strategy in the limits in which one strategy is rare (*f* = 0 and *f* = 1). Notably, we find that it is not possible for the replicator equation to support bistability of the all-resident and all-mutant equilibria at *f* = 0 and *f* = 1 in the case of Cobb-Douglas utility. We provide a proof of Proposition 1 in Section A.3 of S1 Text.

**Proposition 1**. *Suppose that the resident and mutant types have sociality strategies featuring reproduction numbers* $R_{r}^{\left(g\right)}\geq 1$ *and* $R_{m}^{\left(g\right)}\geq 1$ *for the good contagion, with* $R_{r}^{\left(g\right)}\neq R_{m}^{\left(g\right)}$ *and at least one of these reproduction numbers strictly greater than* 1. *Then, for any c* > 0 *and for any resident and mutant types with reproduction numbers* $R_{r}^{\left(b\right)}=cR_{r}^{\left(g\right)}$ *and* $R_{m}^{\left(b\right)}=cR_{r}^{\left(g\right)}$ *for the bad contagion, the difference of Cobb-Douglas log-utilities* log[*U*_*m*_(*f*)] − log[*U*_*r*_(*f*)] *is a decreasing function of f*. *As a consequence, the long-time behavior can be determined by the relative values of U*_*m*_(*f*) *and* *U*_*r*_(*f*) *at the endpoints f* = 0 *and f* = 1. *The three possible cases are the following*:

- *U*_*m*_(0) > *U*_*r*_(0) *and* *U*_*m*_(1) > *U*_*r*_(1): *f* = 1 *is globally stable and the mutant will fix in the population*.
- *U*_*m*_(0) < *U*_*r*_(0) *and* *U*_*m*_(1) < *U*_*r*_(1): *f* = 0 *is globally stable and the resident will fix in the population*.
- *U*_*m*_(0) > *U*_*r*_(0) *and* *U*_*m*_(1) < *U*_*r*_(1): *There exists a unique interior equilibrium* $\hat{f}\in \left(0,1\right)$ *that is globally stable, and mutant and resident will coexist in the long-time population*.

### Adaptive dynamics

Having considered the evolutionary competition between two sociality strategies, we now turn to try to understand how the level of socialization changes over longer evolutionary timescales. To do this, we adopt the framework of adaptive dynamics, looking to characterize evolutionarily-stable levels of socialization. First, we study the contagion dynamics in the limit of infinitessimal mutant frequency (Endemic equilibrium in mutant Population). Then we characterize the socially-optimal and evolutionarily-stable outcomes of the long-time adaptive dynamics under Cobb-Douglas utility (Cobb-Douglas utility). After illustration the social dilemmas of sociality strategies for this choice of utility functions (Illustrating the social dilemma), we study a more general family of utility functions to understand the broadest set of assumptions under which there exists a unique socially-optimal level of social interaction and in which we can expect to see a discrepancy between self-interested and socially-optimal behavior under evolutionary dynamics depending on our coupled good and bad contagion processes (General utility function). In particular, we derive natural conditions required of utility functions to guarantee that the adaptive dynamics will support greater levels of social interaction than is socially optimal when the good contagion spreads more readily than the bad contagion (*c* < 1) and such that the adaptive dynamics produce less social interaction than optimal when the bad contagion spreads more readily (*c* > 1).

#### Endemic equilibrium in mutant Population

In the adaptive dynamics limit, we consider the case in which mutants are initially rare in the population. This allows us to consider the limit in which the fraction *f* of individuals with the mutant strategy tends to zero in our model for dimorphic contagion dynamics of Eqs (9a) and (9b). In this limit, the vanishing presence of the mutant population results in the dynamics for the resident population reducing to the monomorphic dynamics governed by Eq (4). The resident population will then converge to to the long-time equilibrium from Eq (5). For the mutant population, we see that the dynamics of the good contagion reduces to the following equation

$$\begin{array}{c} \frac{1}{\gamma_{g}}\frac{dI_{m}^{\left(g\right)}}{dt}=R_{m}^{\left(g\right)}(1-I_{m}^{\left(g\right)})I_{r}^{\left(g\right)}-I_{m}^{\left(g\right)}, \end{array}$$ (13)

where $R_{r}^{\left(g\right)}$ and $R_{m}^{\left(g\right)}$ are the reproduction numbers for the resident and mutant populations under the good contagion. Because, when $R_{r}^{\left(g\right)}>1$, the equilibrium fraction ${\hat{I}}_{r}^{\left(g\right)}$ of residents with the good contagion is given by ${\hat{I}}_{r}^{\left(g\right)}=1-\frac{1}{R_{r}^{\left(g\right)}}$, we find from Eq (13) that the equilibrium fraction ${\hat{I}}_{m}^{\left(g\right)}$ of mutants with the good contagion must satisfy

$$R_{m}^{\left(g\right)}(1-{\hat{I}}_{m}^{\left(g\right)}){\hat{I}}_{r}^{\left(g\right)}-{\hat{I}}_{m}^{\left(g\right)}=R_{m}^{\left(g\right)}(1-\frac{1}{R_{r}^{\left(g\right)}})\left(1-{\hat{I}}_{m}^{\left(g\right)}\right)-{\hat{I}}_{m}^{\left(g\right)}=0.$$

Solving this equation, we can find the following expression for ${\hat{I}}_{m}^{\left(g\right)}$ as a function of the mutant and resident reproduction numbers $R_{m}^{\left(g\right)}$ and $R_{r}^{\left(g\right)}$:

$$\begin{array}{c} {\hat{I}}_{m}^{\left(g\right)}\left(R_{m}^{\left(g\right)},R_{r}^{\left(g\right)}\right)=\frac{R_{m}^{\left(g\right)}\left(R_{r}^{\left(g\right)}-1\right)}{R_{m}^{\left(g\right)}\left(R_{r}^{\left(g\right)}-1\right)+R_{r}^{\left(g\right)}}. \end{array}$$ (14)

This equilibrium is stable whenever it is biologically feasible, which holds for all $R_{m}^{\left(g\right)}\geq 0$ if $R_{r}^{\left(g\right)}>1$. For the bad contagion, we can use our assumption that the reproduction numbers for the mutant and resident populations are $R_{m}^{b}=cR_{m}^{\left(g\right)}$ and $R_{r}^{b}=cR_{r}^{\left(g\right)}$ to similarly find that the level of infectiousness in the endemic equilibrium for the mutant population under the bad contagion is given by

$$\begin{array}{c} {\hat{I}}_{m}^{\left(b\right)}=\{\begin{array}{cc} 0 & :R_{r}^{\left(g\right)}\leq \frac{1}{c}\\ \frac{R_{m}^{\left(g\right)}(cR_{r}^{\left(g\right)}-1)}{R_{m}^{\left(g\right)}(cR_{r}^{\left(g\right)}-1)+R_{r}^{\left(g\right)}} & :R_{r}^{\left(g\right)}>\frac{1}{c} \end{array}. \end{array}$$ (15)

Because we formulate our utility functions in terms of susceptibility of the bad contagion, it is also helpful for us to use ${\hat{S}}_{m}^{\left(b\right)}=1-{\hat{I}}_{m}^{\left(b\right)}$ to see that equilibrium fraction of the mutant population that is susceptible to the bad contagion is

$$\begin{array}{c} {\hat{S}}_{m}^{\left(b\right)}=\{\begin{array}{cc} 1 & :R_{r}^{\left(g\right)}\leq \frac{1}{c}\\ \frac{R_{r}^{\left(g\right)}}{R_{m}^{\left(g\right)}(cR_{r}^{\left(g\right)}-1)+R_{r}^{\left(g\right)}} & :R_{r}^{\left(g\right)}>\frac{1}{c} \end{array}. \end{array}$$ (16)

In our adaptive dynamics analysis, we will need to know how these endemic equilibria vary with the mutant sociality level $R_{m}^{\left(g\right)}$, so we compute the partial derivatives

$$\begin{array}{c} \frac{\partial {\hat{I}}_{m}^{\left(g\right)}\left(R_{m}^{\left(g\right)},R_{r}^{\left(g\right)}\right)}{\partial R_{m}^{\left(g\right)}}=\frac{R_{r}^{\left(g\right)}\left(R_{r}^{\left(g\right)}-1\right)}{{\left(R_{m}^{\left(g\right)}R_{r}^{\left(g\right)}+R_{r}^{\left(g\right)}-R_{m}^{\left(g\right)}\right)}^{2}} \end{array}$$ (17a)

$$\begin{array}{c} \frac{\partial {\hat{S}}_{m}^{\left(b\right)}\left(R_{m}^{\left(g\right)},R_{r}^{\left(g\right)}\right)}{\partial R_{m}^{\left(g\right)}}=\{\begin{array}{cc} 0 & :R_{r}^{\left(g\right)}\leq \frac{1}{c}\\ \frac{R_{r}^{\left(g\right)}(cR_{r}^{\left(g\right)}-1)}{{\left(cR_{m}^{\left(g\right)}R_{r}^{\left(g\right)}+R_{r}^{\left(g\right)}-R_{m}^{\left(g\right)}\right)}^{2}} & :R_{r}^{\left(g\right)}>\frac{1}{c} \end{array}. \end{array}$$ (17b)

In the limit of local mutation in which $R_{m}^{\left(g\right)}\to R_{r}^{\left(g\right)}$, we may further compute that

$$\begin{array}{c} \frac{\partial {\hat{I}}_{m}^{\left(g\right)}\left(R_{m}^{\left(g\right)},R_{r}^{\left(g\right)}\right)}{\partial R_{m}^{\left(g\right)}}{|}_{R_{m}^{\left(g\right)}=R_{r}^{\left(g\right)}}=\frac{R_{r}^{\left(g\right)}-1}{R_{r}^{3}} \end{array}$$ (18a)

$$\begin{array}{c} \frac{\partial {\hat{S}}_{m}^{\left(b\right)}\left(R_{m}^{\left(g\right)},R_{r}^{\left(g\right)}\right)}{\partial R_{m}^{\left(g\right)}}{|}_{R_{m}^{\left(g\right)}=R_{r}^{\left(g\right)}}=\{\begin{array}{cc} 0 & :R_{r}^{\left(g\right)}\leq \frac{1}{c}\\ \frac{cR_{r}^{\left(g\right)}-1}{c^{2}R_{r}^{3}} & :R_{r}^{\left(g\right)}>\frac{1}{c} \end{array} \end{array}$$ (18b)

#### Cobb-Douglas utility

For the Cobb-Douglas utility, we have the utility of a mutant with reproduction number $R_{m}^{\left(g\right)}$ in a resident population with reproduction number $R_{r}^{\left(g\right)}$ is given by

$$\begin{array}{c} U(R_{m}^{\left(g\right)},R_{r}^{\left(g\right)})={\left({\hat{I}}_{m}^{\left(g\right)}\left(R_{m}^{\left(g\right)},R_{r}^{\left(g\right)}\right)\right)}^{\alpha }{\left({\hat{S}}_{m}^{\left(b\right)}\left(R_{m}^{\left(g\right)},R_{r}^{\left(g\right)}\right)\right)}^{1-\alpha } \end{array}$$ (19)

It is helpful for our analysis to alternatively consider the log-utility

$$\begin{array}{c} \text{log}\;(U[R_{m}^{\left(g\right)},R_{r}^{\left(g\right)}])=\alpha \;\text{log}\;[{\hat{I}}_{m}^{\left(g\right)}\left(R_{m}^{\left(g\right)},R_{r}^{\left(g\right)}\right)]+(1-\alpha )\;\text{log}\;[{\hat{S}}_{m}^{\left(b\right)}\left(R_{m}^{\left(g\right)},R_{r}^{\left(g\right)}\right)] \end{array}$$ (20)

We can now consider a relative advantage of a mutant in the resident population by considering the quantity

$$\begin{array}{c} s_{R_{r}^{\left(g\right)}}\left(R_{m}^{\left(g\right)}\right)≔\;\text{log}\;(U[R_{m}^{\left(g\right)},R_{r}^{\left(g\right)}])-\;\text{log}\;(U[R_{r}^{\left(g\right)},R_{r}^{\left(g\right)}]) \end{array}$$ (21)

Next, we can compute the local selection gradient $s_{R_{r}^{\left(g\right)}}^{\prime }\left(R_{r}^{\left(g\right)}\right)$ by differentiating the relative advantage with respect to $R_{m}^{\left(g\right)}$ and evaluating the derivative when $R_{m}^{\left(g\right)}=R_{r}^{\left(g\right)}$. Differentiating both sides of Eq (21), we can use our expression for log-utility from Eq (20), the endemic equilibria from a monomorphic population from Eq (5), and the partial derivatives from Eq (18) to see that the local selection gradient takes the following form:

$$\begin{array}{cc} s_{R_{r}^{\left(g\right)}}^{\prime }\left(R_{r}^{\left(g\right)}\right) & ≔\frac{\partial s_{R_{r}^{\left(g\right)}}\left(R_{m}^{\left(g\right)}\right)}{\partial R_{m}^{\left(g\right)}}{|}_{R_{m}^{\left(g\right)}=R_{r}^{\left(g\right)}}=\frac{\partial \;\text{log}\;(U[R_{m}^{\left(g\right)},R_{r}^{\left(g\right)}])}{\partial R_{m}^{\left(g\right)}}{|}_{R_{m}^{\left(g\right)}=R_{r}^{\left(g\right)}}\\ & =(\frac{1}{{\hat{I}}_{m}^{\left(g\right)}\left(R_{r}^{\left(g\right)},R_{r}^{\left(g\right)}\right)})\frac{\partial {\hat{I}}_{m}^{\left(g\right)}\left(R_{m}^{\left(g\right)},R_{r}^{\left(g\right)}\right)}{\partial R_{m}^{\left(g\right)}}{|}_{R_{m}^{\left(g\right)}=R_{r}^{\left(g\right)}}\\ & +(\frac{1}{{\hat{S}}_{m}^{\left(b\right)}\left(R_{r}^{\left(g\right)},R_{r}^{\left(g\right)}\right)})\frac{\partial {\hat{S}}_{m}^{\left(b\right)}\left(R_{m}^{\left(g\right)},R_{r}^{\left(g\right)}\right)}{\partial R_{m}^{\left(g\right)}}{|}_{R_{m}^{\left(g\right)}=R_{r}^{\left(g\right)}}.\\ & =\{\begin{array}{cc} \frac{\alpha }{{\left(R_{r}^{\left(g\right)}\right)}^{2}} & :R_{r}^{\left(g\right)}\leq \frac{1}{c}\\ \frac{1}{{\left(R_{r}^{\left(g\right)}\right)}^{2}}[\alpha -(1-\alpha )(R_{r}^{\left(g\right)}-\frac{1}{c})] & :R_{r}^{\left(g\right)}>\frac{1}{c} \end{array} \end{array}$$ (22)

We note that the local selection gradient is always positive for $R_{r}^{\left(g\right)}\leq \frac{1}{c}$ and is a decreasing function of $R_{r}^{\left(g\right)}$. Therefore, we deduce that there is unique evolutionary singular strategy satisfying $s_{R_{r}^{\left(g\right)}}^{\prime }\left(R_{r}^{\left(g\right)}\right)=0$, which is given by

$$\begin{array}{c} {\left(R_{r}^{\left(g\right)}\right)}^{*}=\frac{\alpha }{1-\alpha }+\frac{1}{c} \end{array}$$ (23)

Because we only consider levels of sociality for the good contagion with $R_{r}^{\left(g\right)}\geq 1$, the singular strategy given in Eq (23) is infeasible when ${\left(R_{r}^{\left(g\right)}\right)}^{*}<1$ and, consequently, the selection gradient $s_{R_{r}^{\left(g\right)}}^{\prime }\left(R_{r}^{\left(g\right)}\right)$ is negative at $R_{r}^{\left(g\right)}=1$. Using Eq (22), we see that this singular strategy is infeasible when

$$\begin{array}{c} s_{R_{r}^{\left(g\right)}}^{\prime }\left(R_{r}^{\left(g\right)}\right){|}_{R_{r}^{\left(g\right)}=1}=\alpha -(1-\alpha )(1-\frac{1}{c})<0\Rightarrow (1-2\alpha )\;c>1-\alpha . \end{array}$$ (24)

Notably, this can only occur if $\alpha <\frac{1}{2}$, when individuals are more concerned with avoiding the bad contagion than with acquiring the good contagion. When $\alpha <\frac{1}{2}$ we can rearrange the inequality from Eq (24) to obtain the following condition for the infeasibility of the singular strategy ${\left(R_{r}^{\left(g\right)}\right)}^{*}$:

$$\begin{array}{c} c>\frac{1-\alpha }{1-2\alpha }. \end{array}$$ (25)

When $\alpha <\frac{1}{2}$ and the condition of Eq (25) holds for *c*, we can use this inequality to see that the selection gradient satisfies

$$\begin{array}{c} s_{R_{r}^{\left(g\right)}}^{\prime }\left(R_{r}^{\left(g\right)}\right)=\frac{1}{{\left(R_{r}^{\left(g\right)}\right)}^{2}}[\alpha -(1-\alpha )(R_{r}^{\left(g\right)}-\frac{1}{c})]\leq \frac{1}{{\left(R_{r}^{\left(g\right)}\right)}^{2}}(1-\alpha )(1-R_{r}^{\left(g\right)}). \end{array}$$ (26)

Then, for any interior sociality strategy $R_{r}^{\left(g\right)}>1$, we see from Eq (26) that

$$s_{R_{r}^{\left(g\right)}}^{\prime }\left(R_{r}^{\left(g\right)}\right)<0\;\;\text{when}\;\;c>\frac{1-\alpha }{1-2\alpha }\;,\;R_{r}^{\left(g\right)}>1.$$

As a result, the selection gradient is always decreasing and $R_{r}^{\left(g\right)}=1$ is the resulting ESS level of sociality when Eq (25) holds. Therefore we find that our evolutionarily-stable strategy has the following piecewise characterization

$$\begin{array}{c} R_{\text{ESS}}^{\left(g\right)}=\;\text{max}\;(1,\frac{1}{c}+\frac{\alpha }{1-\alpha }). \end{array}$$ (27)

We further study the classification of $R_{\text{ESS}}^{\left(g\right)}$ as an evolutionarily-stable strategy and convergence stable strategy using the relevant conditions on the local selection gradient in Section B of S1 Text.

In Table 1, we compare the evolutionarily-stable and socially-optimal levels of sociality $R_{\text{ESS}}^{\left(g\right)}$ and $R_{\text{opt}}^{\left(g\right)}$ for the different possible values of the relative spreading abilities for the good and bad contagion *c* and the weight *α* placed upon the good and bad contagion in the Cobb-Douglas utility. Across each of the cases, we see from Table 1 that $R_{\text{ESS}}^{\left(g\right)}<R_{\text{opt}}^{\left(g\right)}$ when *c* > 1, $R_{\text{ESS}}^{\left(g\right)}=R_{\text{opt}}^{\left(g\right)}$ when *c* = 1, and $R_{\text{ESS}}^{\left(g\right)}>R_{\text{opt}}^{\left(g\right)}$ when *c* < 1. In particular, this means that the evolutionarily-stable level of interactions exceeds the social optimum when the good contagion spreads more effectively than the bad contagion, while the evolutionarily-stable level of interaction is less than the social optimum when the bad contagion spreads more effectively than the good contagion.

**Table 1:** Evolutionarily-stable and socially-optimal sociality strategies $R_{\text{ESS}}^{\left(g\right)}$ and $R_{\text{opt}}^{\left(g\right)}$ for different cases on the relative values of the relative weight *α* of the good contagion under Cobb-Douglas utility and the relative infectiousness *c* of the bad contagion.

	*αc* > (1 − *α*)(*c* − 1)	*αc* < (1 − *α*)(*c* − 1)
*c* > 1 − *α*	$\begin{array}{cc} R_{\text{ESS}}^{\left(g\right)} & =\frac{1}{c}+\frac{\alpha }{1-\alpha }\\ R_{\text{opt}}^{\left(g\right)} & =\frac{1}{1-\alpha } \end{array}$	$\begin{array}{cc} R_{\text{ESS}}^{\left(g\right)} & =1\\ R_{\text{opt}}^{\left(g\right)} & =\frac{1}{1-\alpha }>1 \end{array}$
*c* < 1 − *α*	$\begin{array}{cc} R_{\text{ESS}}^{\left(g\right)} & =\frac{1}{c}+\frac{\alpha }{1-\alpha }>1\\ R_{\text{opt}}^{\left(g\right)} & =\frac{1}{c} \end{array}$	N/A

Finally, we can use the relationship between the reproduction number $R_{\cdot }^{\left(g\right)}$ and the social interaction rate *σ*⋅ to describe the socially-optimal and evolutionarily-stable sociality strategies in terms of the underlying rate of social interactions for individuals. Noting that $\sigma_{\cdot }=\frac{\gamma_{g}R_{\cdot }^{\left(g\right)}}{p_{g}}$, we can use Eqs (8) and (27) to provide the following characterization of the socially-optimal $\sigma_{g}^{\text{opt}}$ and $\sigma_{g}^{\text{ESS}}$:

$$\begin{array}{c} \sigma_{g}^{\text{opt}}=\frac{\gamma_{g}}{p_{g}}\;\text{max}\;(\frac{1}{c},\frac{1}{1-\alpha }) \end{array}$$ (28a)

$$\begin{array}{c} \sigma_{g}^{\text{ESS}}=\frac{\gamma_{g}}{p_{g}}\;\text{max}\;(1,\frac{1}{c}+\frac{\alpha }{1-\alpha }). \end{array}$$ (28b)

Because the socially-optimal and evolutionarily-stable social interaction strategies are proportional to the equivalent quantities expressed in terms of the reproduction number of the good contagion, we see that the qualitative results and social dilemmas can be seen in either formulation of the sociality strategies.

#### Illustrating the social dilemma

**Fig 4:**
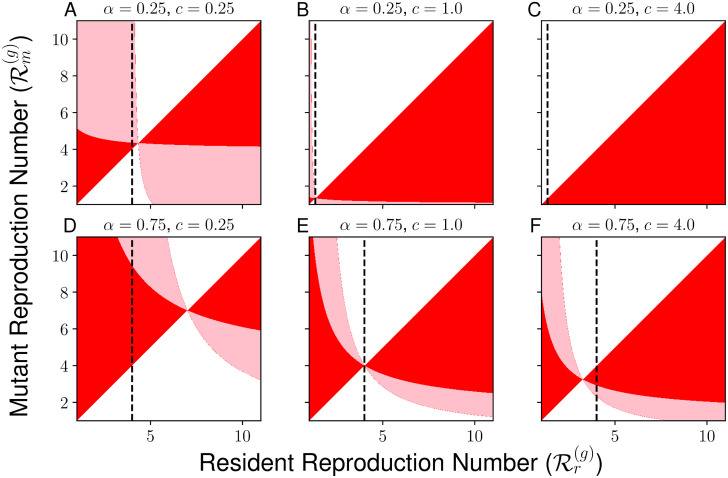
Pairwise invasibility plot with mutual invasbility or lack thereof shown. In each panel, horizontal axis describes the reproduction number $R_{r}$ of the resident strategy, while the vertical axis describes the reproduction number $R_{m}$ corresponding to the mutant strategy. The color of a given point describes the outcome of pairwise competition between the resident and mutant strategy. Points displayed in white describe pairs of strategies in which the resident strategy dominates the mutant strategy: a small cohort of the mutant will fail to invade a population primarily consisting of resident strategy, while a small cohort of the resident strategy will successfully invade a population primarily consisting of the mutant strategy. Points in red describe pairs of strategies in which the mutant strategy dominates the resident strategy: the mutant strategy will successfully invade the resident when rare, and a population of the mutant strategy will resist the invasion of a small cohort of the resident strategy. Points displayed in pink describe a case in which neither the resident nor mutant strategy dominates the other: the mutant invades the resident when rare and the resident invades the mutant when rare. The dashed line describes the socially-optimal strategy $R_{\text{opt}}^{\left(g\right)}$, while the point of intersection of two components of the red region corresponds to the evolutionarily-stable strategy $R_{\text{ESS}}^{\left(g\right)}$. All areas of mutual invasibility are off the diagonal except when arbitrarily close to the evolutionarily-stable strategy ESS, which implies that dimorphism will not evolve if mutations are small.

Now, we illustrate how the social dilemma seen in our model with Cobb-Douglas utility depends on *α* and *c*. One tool for visualizing the relative competitive abilities of different sociality strategies under adaptive dynamics is a pairwise-invasibility plot (PIP), which compares the relative competitive ability of possible mutant and resident strategies in the limit of a rare mutant. In Fig 4, we present example PIPs for various values of *c* (0.25, 1, and 4) and *α* (0.25 and 0.75) for the case of Cobb-Douglas utility, displaying the possible combinations of of sociality strategies $R_{r}^{\left(g\right)}$ and $R_{m}^{\left(g\right)}$ for which $R_{r}^{\left(g\right)}$ outcompetes $R_{m}^{\left(g\right)}$ when either strategy is rare (shaded in red), for which $R_{m}^{\left(g\right)}$ outcompetes $R_{r}^{\left(g\right)}$ when either is rare (shaded in white), and for which each of the two strategies can invade the other when rare (shaded in pink). Consistent with the classification using the local selection gradient, we see that $R_{\text{ESS}}^{\left(g\right)}>R_{\text{opt}}^{\left(g\right)}$ when *c* = 0.25, $R_{\text{ESS}}^{\left(g\right)}=R_{\text{opt}}^{\left(g\right)}$ when *c* = 1, and $R_{\text{ESS}}^{\left(g\right)}<R_{\text{opt}}^{\left(g\right)}$ when *c* = 4.

**Fig 5:**
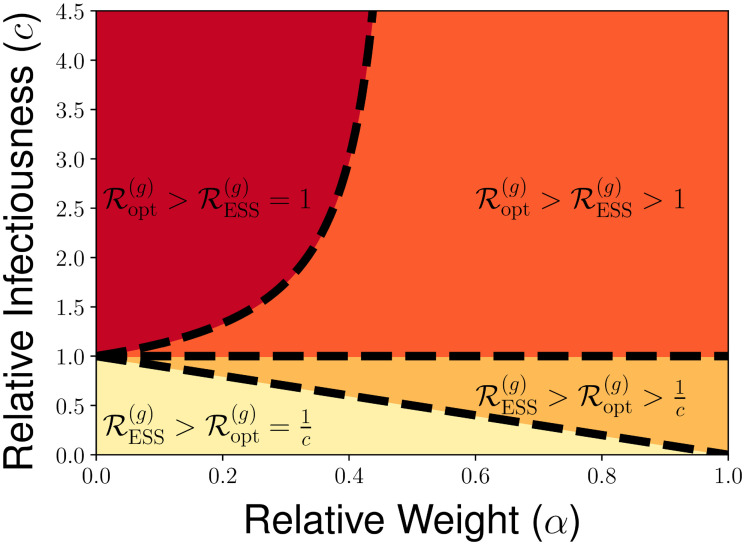
Illustration of the four possible qualitative behaviors for $R_{\text{opt}}^{\left(g\right)}$ and $R_{\text{ESS}}^{\left(g\right)}$ across the range of relative levels of infectiousness for the bad contagion *c* and relative weight placed on the good contagion *α* under Cobb-Douglas utility. The various regions are defined by the relative size of $R_{\text{ESS}}^{\left(g\right)}$ and $R_{\text{opt}}^{\left(g\right)}$, as well as whether $R_{\text{ESS}}^{\left(g\right)}=1$ or $R_{\text{ESS}}^{\left(g\right)}>1$ and whether $R_{\text{opt}}^{\left(g\right)}=\frac{1}{c}$ or $R_{\text{opt}}^{\left(g\right)}>\frac{1}{c}$, with the boundaries between regions as characterized by Table 1.

**Fig 6:**
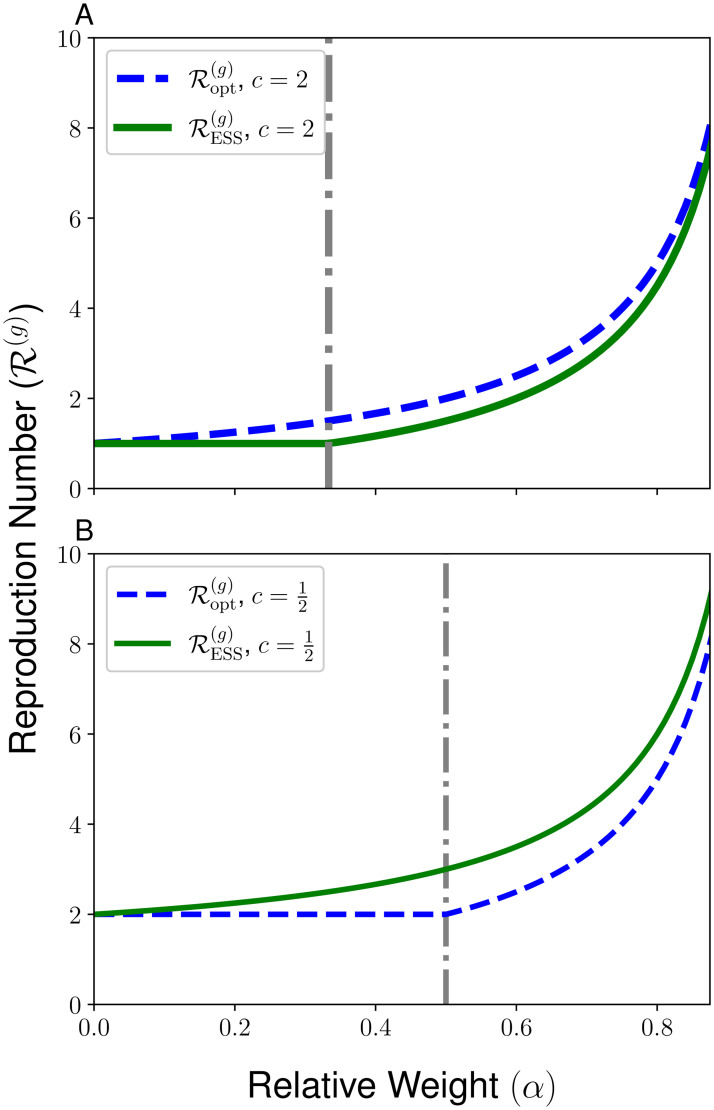
Reproduction numbers $R_{\text{ESS}}^{\left(g\right)}$ (solid green line) and $R_{\text{opt}}^{\left(g\right)}$ (dashed blue line) for the good contagion. We plot these reproduction numbers as a function of the relative importance of the good contagion *α* and for the relative infectiousness values *c* = 2 (**A**) and $c=\frac{1}{2}$ (**B**).

In addition, we present in Fig 5 an illustration of the different possible regimes for $R_{\text{ESS}}^{\left(g\right)}$ and $R_{\text{opt}}^{\left(g\right)}$ as determined by the parameters *c* and *α*, highlighting the various cases presented in Table 1. In Fig 6, we show how $R_{\text{ESS}}^{\left(g\right)}$ and $R_{\text{opt}}^{\left(g\right)}$ vary with the relative weight *α* for the cases of $c=\frac{1}{2}$ and *c* = 2 for the relative infectiousness of the bad contagion. Through the illustrations in Fig 6, we highlight the direction of the social dilemma by showing that evolutionary dynamics promote too much interaction when *c* < 1 and promote too little interaction when *c* < 1. Furthermore, we highlight the extreme forms of the social dilemma that can be achieved for certain parameters, in which either $R_{\text{ESS}}^{\left(g\right)}=1$ and the good contagion cannot spread in the population or in which $R_{\text{opt}}^{\left(g\right)}>\frac{1}{c}$ and the bad contagion could be eliminated with socially-optimal behavior but remains present in the evolutionarily-stable strategy.

#### General utility function

Here we study the possibility of social dilemmas for a more general family of utility functions describing agents who benefit from the good contagion and suffer from the bad contagion. What we would to describe through this family of utility functions is the desired property that, all else being equal, individuals would prefer increased exposure to the good contagion and decreased exposure to the bad contagion. Mathematically, we can formulate this by introducing the utility function

$$U\left[R_{m}^{\left(g\right)},R_{r}^{\left(g\right)}\right]≔U\left({\hat{I}}_{m}^{\left(g\right)}\left(R_{m}^{\left(g\right)},R_{r}^{\left(g\right)}\right),{\hat{S}}_{m}^{\left(b\right)}\left(cR_{m}^{\left(g\right)},cR_{r}^{\left(g\right)}\right)\right)$$

with continuous partial derivatives satisfying

-
$\frac{\partial U(\cdot ,\cdot )}{\partial {\hat{I}}^{\left(g\right)}\left(\cdot \right)}>0$ : an individual’s utility is improved by increased exposure to the good contagion
-
$\frac{\partial U(\cdot ,\cdot )}{\partial {\hat{S}}^{\left(b\right)}\left(\cdot \right)}>0$ : an individual’s utility is improved by decreased exposure to the bad contagion

First we examine the question of social optimality. If all individuals have the resident level of sociality $R_{r}^{\left(g\right)}$, then the levels of contagion converge to the equilibria ${\hat{I}}^{\left(g\right)}\left(R_{r}^{\left(g\right)}\right)$ and ${\hat{S}}^{\left(b\right)}\left(cR_{r}^{\left(g\right)}\right)=1-{\hat{I}}^{\left(b\right)}\left(cR_{r}^{\left(g\right)}\right)$ from Eq (5), and the utility for individuals can be written as

$$\begin{array}{c} U\left[R_{r}^{\left(g\right)},R_{r}^{\left(g\right)}\right]=U({\hat{I}}^{\left(g\right)}\left(R_{r}^{\left(g\right)}\right),{\hat{S}}^{\left(b\right)}\left(cR_{r}^{\left(g\right)}\right)) \end{array}$$ (29)

To find potential socially-optimal levels of sociality $R_{r}^{\left(g\right)}$, we differentiate Eq (29) with respect to $R_{r}^{\left(g\right)}$. To study social optima, we have to consider separately the cases in which both contagions spread (when $R_{r}^{\left(g\right)}>\;\text{max}\;\left\{1,\frac{1}{c}\right\}$), in which either only the good contagion spreads (when $\frac{1}{c}\geq R_{r}^{\left(g\right)}>1$) or only the bad contagion spreads (when $1\geq R_{r}^{\left(g\right)}>\frac{1}{c}$), or in which both contagions do not spread (when $R_{r}^{\left(g\right)}\leq \;\text{min}\;\left\{1,\frac{1}{c}\right\}$). We note that we were able to primarily ignore the latter two cases for the Cobb-Douglas utility function, as $U(R_{r}^{\left(g\right)})$ vanished when $R_{r}^{\left(g\right)}\leq 1$ for that family of functions.

Using the expressions for the monomorphic contagion equilibria from Eq (5), we see that

$$\begin{array}{cc} \frac{\partial U[R_{r}^{\left(g\right)},R_{r}^{\left(g\right)}]}{\partial R_{r}^{\left(g\right)}} & =\frac{\partial U[R_{r}^{\left(g\right)},R_{r}^{\left(g\right)}]}{\partial {\hat{I}}^{\left(g\right)}\left(R_{r}^{\left(g\right)}\right)}\frac{\partial {\hat{I}}^{\left(g\right)}\left(R_{r}^{\left(g\right)}\right)}{\partial R_{r}^{\left(g\right)}}+\frac{\partial U[R_{r}^{\left(g\right)},R_{r}^{\left(g\right)}]}{\partial {\hat{S}}^{\left(b\right)}\left(R_{r}^{\left(g\right)}\right)}\frac{\partial {\hat{S}}^{\left(b\right)}\left(R_{r}^{\left(g\right)}\right)}{\partial R_{r}^{\left(g\right)}}\\ & =\{\begin{array}{cc} 0 & :R_{r}^{\left(g\right)}\leq 1,\frac{1}{c}\\ -\frac{1}{c{\left(R_{r}^{\left(g\right)}\right)}^{2}}\frac{\partial U[R_{r}^{\left(g\right)},R_{r}^{\left(g\right)}]}{\partial {\hat{S}}^{\left(b\right)}\left(R_{r}^{\left(g\right)}\right)} & :\frac{1}{c}<R_{r}^{\left(g\right)}\leq 1\\ \frac{1}{{\left(R_{r}^{\left(g\right)}\right)}^{2}}\frac{\partial U[R_{r}^{\left(g\right)},R_{r}^{\left(g\right)}]}{\partial {\hat{I}}^{\left(g\right)}\left(R_{r}^{\left(g\right)}\right)} & :1<R_{r}^{\left(g\right)}\leq \frac{1}{c}\\ \frac{1}{{\left(R_{r}^{\left(g\right)}\right)}^{2}}[\frac{\partial U[R_{r}^{\left(g\right)},R_{r}^{\left(g\right)}]}{\partial {\hat{I}}^{\left(g\right)}\left(R_{r}^{\left(g\right)}\right)}-\frac{1}{c}\frac{\partial U[R_{r}^{\left(g\right)},R_{r}^{\left(g\right)}]}{\partial {\hat{S}}^{\left(b\right)}\left(R_{r}^{\left(g\right)}\right)}] & :R_{r}^{\left(g\right)}>1,\frac{1}{c} \end{array}. \end{array}$$ (30)

From our assumption on the partial derivatives of $U[R_{r}^{\left(g\right)},R_{r}^{\left(g\right)}]$, we see that $U[R_{r}^{\left(g\right)},R_{r}^{\left(g\right)}]$ is increasing for the case in which $1<R_{r}^{\left(g\right)}\leq \frac{1}{c}$ (which is possible when *c* < 1), and is decreasing for the case in which $\frac{1}{c}<R_{r}^{\left(g\right)}\leq 1$ (only possible when *c* > 1). This means that, for the case in which *c* ≤ 1, we can we look for socially-optimal levels of sociality among $R_{r}^{\left(g\right)}\in \left[\frac{1}{c},\infty \right)$. For the case in which *c* > 1, it is also possible that the maximizer of the social utility is $R_{r}^{\left(g\right)}=\frac{1}{c}<1$, in which case it is socially optimal to have neither contagion spread in the population. We will show in Section B of S1 Text that such a social optimum is possible for some linear utility functions and the Constant Elasticity of Substitution (CES) family of utility functions [34, 35]. Even in these extreme case in which non-interaction can be collectively optimal, we can now study the conditions under which the social utility function will have at least a unique local optimum for sociality strategies $R_{r}\in \left(1,\infty \right)$ for which the good contagion can spread.

Now we look to characterize the existence and uniqueness of maximizers of the utility $U[R_{r}^{\left(g\right)},R_{r}^{\left(g\right)}]$ when both contagions are present in the population, which occurs for $R_{r}^{\left(g\right)}>\;\text{max}\;\left\{1,\frac{1}{c}\right\}$. From the term in square brackets in Eq (30), we see that one sufficient condition on $U[R_{r}^{\left(g\right)},R_{r}^{\left(g\right)}]$ for uniqueness of a socially-optimal level of sociality $R_{r}^{\left(g\right)}$ is that the $\frac{\partial U[R_{r}^{\left(g\right)},R_{r}^{\left(g\right)}]}{\partial {\hat{I}}^{\left(g\right)}\left(R_{r}^{\left(g\right)}\right)}$ is a decreasing function of $R_{r}^{\left(g\right)}$ and that $\frac{\partial U[R_{r}^{\left(g\right)},R_{r}^{\left(g\right)}]}{\partial {\hat{S}}^{\left(b\right)}\left(R_{r}^{\left(g\right)}\right)}$ is an increasing function of $R_{r}^{\left(g\right)}$. Under these assumptions, the derivative in Eq (30) is decreasing in $R_{r}^{\left(g\right)}$ and therefore changes sign at most once for $R_{r}^{\left(g\right)}\in \left[\frac{1}{c},\infty \right)$. In that case, either there exists a unique interior social optimum, or the social optimal is achieved at one of the endpoints $R_{r}^{\left(g\right)}=1$ (only if *c* > 1), $R_{r}^{\left(g\right)}=\frac{1}{c}$, $R_{r}^{\left(g\right)}=\infty$.

If we allow for greater regularity on the utility function, we can obtain a sufficient condition for the existence of a unique social optimum featuring a finite rate of social interaction. Taking the following partial derivatives

$$\begin{array}{c} \frac{\partial }{\partial R_{r}^{\left(g\right)}}(\frac{\partial U[R_{r}^{\left(g\right)},R_{r}^{\left(g\right)}]}{\partial {\hat{I}}^{\left(g\right)}\left(R_{r}^{\left(g\right)}\right)})=\frac{\partial^{2}U\left[R_{r}^{\left(g\right)},R_{r}^{\left(g\right)}\right]}{\partial {\left({\hat{I}}^{\left(g\right)}\left(R_{r}^{\left(g\right)}\right)\right)}^{2}}\overset{>0}{\overset{︷}{\left(\frac{\partial {\hat{I}}^{\left(g\right)}\left(R_{r}^{\left(g\right)}\right)}{\partial R_{r}^{\left(g\right)}}\right)}}\\ \frac{\partial }{\partial R_{r}^{\left(g\right)}}(\frac{\partial U[R_{r}^{\left(g\right)},R_{r}^{\left(g\right)}]}{\partial {\hat{S}}^{\left(b\right)}\left(R_{r}^{\left(g\right)}\right)})=\frac{\partial^{2}U\left[R_{r}^{\left(g\right)},R_{r}^{\left(g\right)}\right]}{\partial {\left({\hat{S}}^{\left(b\right)}\left(R_{r}^{\left(g\right)}\right)\right)}^{2}}\underset{<0}{\underset{︸}{\left(\frac{\partial {\hat{S}}^{\left(b\right)}\left(R_{r}^{\left(g\right)}\right)}{\partial R_{r}^{\left(g\right)}}\right)}}, \end{array}$$

we see that taking the assumption that the second partial derivatives above are negative will allow us to deduce from Eq (30) that $\frac{\partial }{\partial R_{r}^{\left(g\right)}}U\left[R_{r}^{\left(g\right)},R_{r}^{\left(g\right)}\right]$ is a strictly decreasing function of $R_{r}^{\left(g\right)}$. Therefore we see that a natural sufficient condition for existence of unique, finite social optimum $R_{\text{opt}}^{\left(g\right)}$ is the *U*(⋅, ⋅) be twice-differentiable and strictly concave. Such an assumption holds for the CES utility function [34, 35], as well as for a variety of families of utility functions that contain Cobb-Douglas and CES as special cases [36]. However, a linear utility function $U\left[R_{r}^{\left(g\right)},R_{r}^{\left(g\right)}\right]=\alpha {\hat{I}}^{\left(g\right)}\left(R_{r}^{\left(g\right)}\right)+\left(1-\alpha \right){\hat{S}}^{\left(b\right)}\left(R_{r}^{\left(g\right)}\right)$ is not strictly concave, and we show in Section B of S1 Text that, for a such a utility function, infinite social interaction can be collectively optimal for a range of *c* and *α*.

Furthermore, for the case in which *c* ≤ 1 and the bad contagion will not spread if $R_{r}^{\left(g\right)}\leq 1$, we can use these regular and concavity assumptions along with Eq (30) to deduce that this unique local maximum of $U[R_{r}^{\left(g\right)},R_{r}^{\left(g\right)}]$ is, in fact, the global maximizer of the social utility. For the case of *c* > 1, the strategy $R_{r}^{\left(g\right)}=\frac{1}{c}<1$ will also be a local maximum because $U[R_{r},R_{r}]$ is a decreasing function on $\frac{1}{c}<R_{r}^{\left(g\right)}<1$ (see Eq (30)). In this case, we need to compare the value of the utility functions at these two points to determine the global maximizer of $U[R_{r}^{\left(g\right)},R_{r}^{\left(g\right)}]$. This presence of two local optima also has consequences for evolutionary dynamics, as we will show in Section B of S1 Text that it is possible to achieve evolutionary bistability between $R_{r}^{\left(g\right)}=\frac{1}{c}$ and a sociality strategy featuring presence of the good contagion under linear and CES utility functions when *c* > 1.

Now we will consider the question of evolutionarily-stable strategies. To study ESSes, we compute the local selection gradient

$$\begin{array}{cc} s_{R_{r}^{\left(g\right)}}^{\prime }\left(R_{r}^{\left(g\right)}\right) & =\frac{\partial U[R_{m}^{\left(g\right)},R_{r}^{\left(g\right)}]}{\partial {\hat{I}}^{\left(g\right)}\left(R_{m}^{\left(g\right)},R_{r}^{\left(g\right)}\right)}\frac{\partial {\hat{I}}^{\left(g\right)}\left(R_{m}^{\left(g\right)},R_{r}^{\left(g\right)}\right)}{\partial R_{m}^{\left(g\right)}}{|}_{R_{m}^{\left(g\right)}=R_{r}^{\left(g\right)}}\\ & +\frac{\partial U[R_{m}^{\left(g\right)},R_{r}^{\left(g\right)}]}{\partial {\hat{S}}^{\left(b\right)}\left(R_{m}^{\left(g\right)},R_{r}^{\left(g\right)}\right)}\frac{\partial {\hat{S}}^{\left(b\right)}\left(R_{m}^{\left(g\right)},R_{r}^{\left(g\right)}\right)}{\partial R_{m}^{\left(g\right)}}{|}_{R_{m}^{\left(g\right)}=R_{r}^{\left(g\right)}}. \end{array}$$ (31)

We can use Eq (18) to rewrite out expression for the local selection gradient, which will do by separately considering the cases in which $R_{r}^{\left(g\right)}\leq \frac{1}{c}$ and $R_{r}^{\left(g\right)}>\frac{1}{c}$. When $R_{r}^{\left(g\right)}\leq \frac{1}{c}$, our selection gradient takes the form

$$\begin{array}{c} s_{R_{r}^{\left(g\right)}}^{\prime }\left(R_{r}^{\left(g\right)}\right)=(\frac{R_{r}^{\left(g\right)}-1}{R_{3}^{\left(r\right)}})\frac{\partial U[R_{r}^{\left(g\right)},R_{r}^{\left(g\right)}]}{\partial {\hat{I}}^{\left(g\right)}\left(R_{r}^{\left(g\right)},R_{r}^{\left(g\right)}\right)}. \end{array}$$ (32a)

When $R_{r}^{\left(g\right)}>\frac{1}{c}$, we can write our selection gradient as

$$\begin{array}{c} s_{R_{r}^{\left(g\right)}}^{\prime }\left(R_{r}^{\left(g\right)}\right)=\frac{1}{{\left(R_{r}^{\left(g\right)}\right)}^{2}}[(\frac{R_{r}^{\left(g\right)}-1}{R_{r}^{\left(g\right)}})\frac{\partial U[R_{r}^{\left(g\right)},R_{r}^{\left(g\right)}]}{\partial {\hat{I}}^{\left(g\right)}\left(R_{r}^{\left(g\right)},R_{r}^{\left(g\right)}\right)}\\ -(\frac{R_{r}^{\left(g\right)}-\frac{1}{c}}{R_{r}^{\left(g\right)}})\frac{1}{c}\frac{\partial U[R_{r}^{\left(g\right)},R_{r}^{\left(g\right)}]}{\partial {\hat{S}}^{\left(b\right)}\left(R_{r}^{\left(g\right)},R_{r}^{\left(g\right)}\right)}] \end{array}$$ (32b)

We notice from our assumption on $\frac{\partial U(\cdot )}{\partial I^{\left(g\right)}\left(\cdot \right)}$ that the selection gradient is always positive for $R_{r}^{\left(g\right)}\leq \frac{1}{c}$, and we can compute that

$$\begin{array}{c} \underset{R_{r}^{\left(g\right)}\to {\frac{1}{c}}^{+}}{\text{lim}}s_{R_{r}^{\left(g\right)}}^{\prime }\left(R_{r}^{\left(g\right)}\right)=c^{2}(1-c)\frac{\partial U[R_{r}^{\left(g\right)},R_{r}^{\left(g\right)}]}{\partial {\hat{I}}^{\left(g\right)}\left(R_{r}^{\left(g\right)},R_{r}^{\left(g\right)}\right)}>0. \end{array}$$ (33)

In particular, this means that, for the case in which $R_{\text{opt}}^{\left(g\right)}=\frac{1}{c}$ for *c* < 1, the social optimum cannot be evolutionarily-stable. We will look to find ESS sociality strategies with $R_{r}^{\left(g\right)}\in \left(\frac{1}{c},\infty \right)$.

To explore social dilemmas when $R_{\text{opt}}^{\left(g\right)}>\frac{1}{c}$, we can look to re-express the local selection gradient in a form providing a comparison to first-order condition for the social optimization problem. We obtain

$$\begin{array}{cc} s_{R_{r}^{\left(g\right)}}^{\prime }\left(R_{r}^{\left(g\right)}\right) & =(\frac{R_{r}^{\left(g\right)}-1}{R_{r}^{3}})[\frac{\partial U[R_{r}^{\left(g\right)},R_{r}^{\left(g\right)}]}{\partial {\hat{I}}^{\left(g\right)}\left(R_{r}^{\left(g\right)},R_{r}^{\left(g\right)}\right)}-\frac{1}{c}\frac{\partial U[R_{r}^{\left(g\right)},R_{r}^{\left(g\right)}]}{\partial {\hat{S}}^{\left(b\right)}\left(R_{r}^{\left(g\right)},R_{r}^{\left(g\right)}\right)}]\\ & +\frac{1}{cR_{r}^{3}}(\frac{1}{c}-1)\frac{\partial U[R_{r}^{\left(g\right)},R_{r}^{\left(g\right)}]}{\partial {\hat{S}}^{\left(b\right)}\left(R_{r}^{\left(g\right)},R_{r}^{\left(g\right)}\right)}. \end{array}$$ (34)

Using Eq (30), we can therefore relate the local selection gradient to the derivative of the monomorphic utility through the equation

$$\begin{array}{cc} s_{R_{r}^{\left(g\right)}}^{\prime }\left(R_{r}^{\left(g\right)}\right) & =(\frac{R_{r}^{\left(g\right)}-1}{R_{r}^{\left(g\right)}})\frac{\partial U[R_{r}^{\left(g\right)},R_{r}^{\left(g\right)}]}{\partial R_{r}^{\left(g\right)}}\\ & +\frac{1}{c{\left(R_{r}^{\left(g\right)}\right)}^{3}}(\frac{1}{c}-1)\frac{\partial U[R_{r}^{\left(g\right)},R_{r}^{\left(g\right)}]}{\partial {\hat{S}}^{\left(b\right)}\left(R_{r}^{\left(g\right)},R_{r}^{\left(g\right)}\right)}. \end{array}$$ (35)

From our assumption on the utility function that $\frac{\partial U[\cdot ]}{\partial S^{\left(b\right)}\left(\cdot \right)}>0$ for $R_{r}^{\left(g\right)}>\;\text{max}\;\left\{1,\frac{1}{c}\right\}$, we can therefore deduce that, for such $R_{r}^{\left(g\right)}$,

$$\begin{array}{c} \frac{\partial U[R_{r}^{\left(g\right)},R_{r}^{\left(g\right)}]}{\partial R_{r}^{\left(g\right)}}\geq 0\;\;\text{implies}\;\;s_{R_{r}^{\left(g\right)}}^{\prime }\left(R_{r}^{\left(g\right)}\right)>0\;\text{for}\;c<1 \end{array}$$ (36a)

$$\begin{array}{c} \frac{\partial U[R_{r}^{\left(g\right)},R_{r}^{\left(g\right)}]}{\partial R_{r}^{\left(g\right)}}\leq 0\;\;\text{implies}\;\;s_{R_{r}^{\left(g\right)}}^{\prime }\left(R_{r}^{\left(g\right)}\right)<0\;\text{for}\;c>1. \end{array}$$ (36b)

In particular, this means that interior local maxima of the monomorphic utility function $U[R_{r}^{\left(g\right)},R_{r}^{\left(g\right)}]$ are not evolutionarily-stable strategies unless *c* = 1, which is a structurally unstable case. When *c* = 1, the local selection gradient coincides with the derivative of the monomorphic utility function, and we see that social optima coincide with evolutionarily-stable strategies. Furthermore, under the additional assumptions on $U[R_{m}^{\left(g\right)},R_{r}^{\left(g\right)}]$ guaranteeing the existence of a unique social optimum $R_{\text{opt}}^{\left(g\right)}$, we can deduce from Eq (36a) that any evolutionarily-stable states $R_{\text{ESS}}^{\left(g\right)}$ satisfy $R_{\text{ESS}}^{\left(g\right)}>R_{\text{opt}}^{\left(g\right)}$ for *c* < 1 and $R_{\text{ESS}}^{\left(g\right)}<R_{\text{opt}}^{\left(g\right)}$ for *c* > 1. In particular, this means that the evolutionary dynamics feature a social dilemma in which the evolutionarily-stable and socially-optimal outcomes disagree when *c* ≠ 1, generalizing the qualitative form of the social dilemma that we observed for the case of Cobb-Douglas utility.

## Discussion

In this paper, we have considered the question of how individuals choose their level of social interactions in response to the benefits of exposure to a good contagion and the costs of exposure to a bad contagion. Through both the frameworks of shorter-term replicator dynamics and long-time adaptive dynamics, we characterize evolutionarily-stable levels of sociality, and show how these evolutionary outcomes can be misaligned with the sociality strategies that optimize collective utility. Of key importance to the evolutionary dynamics is the relative transmissibility of the good and bad contagion, as the evolutionarily-stable sociality level features too much interaction when the good contagion spreads more readily than the bad contagion, while evolutionary dynamics favor too little interaction when the bad contagion in the alternative scenario. From this analysis, we see that even in a simple model of social interaction and contagion spread, the benefits of good contagion and the fear of bad can result in a social dilemma in the evolution of sociality strategies.

Most strikingly, there were two extreme examples of the social dilemma in which individually-rational behavior led to different qualitative behavior contagion behavior than is seen in the socially-optimal scenario. When the good contagion spread sufficiently more readily than the bad contagion, socially-optimal sociality strategies can eradicate the bad contagion at equilibrium. However, in such cases, the evolutionarily-stable strategy may still introduce positive levels of the bad contagion due to overpursuit of the benefits of the good contagion. When the bad contagion spreads sufficiently more rapidly than the good contagion, the evolutionarily-stable outcome featured no social interaction whatsoever, even though the socially-optimal sociality strategy always features a positive level of social interaction. Under the Cobb-Douglas utility function, a strategy featuring zero social interaction constitutes a utility minimizer, so the evolutionary dynamics end up achieving the worst possible outcome when the bad contagion is sufficiently contagious.

Having identified a social dilemma in the evolution of sociality strategies, a natural follow-up question is what mechanisms can be used to help mitigate the suboptimal outcomes achieved under evolutionary dynamics. We can draw inspiration from the literature of the evolution of cooperation to study how additional effects of population structure including assortment, reciprocity, and multilevel selection can help to promote efficient levels of socialization [37, 38]. In particular, the case of a complete collapse of social interactions as an evolutionarily-stable outcome suggests that there may be scenarios in which the tradeoff between good and bad contagion would require additional mechanisms beyond well-mixed individual-level selection in order to permit the existence of groups featuring social interactions. As a first attempt to explore one such mechanism, we show in Section B of S1 Text that an assortative process preferencing interactions with same-strategy individuals can help to mitigate the effects of the social dilemma and more closely align the evolutionarily-stable and socially-optimal levels of sociality. By matching together more frequently individuals who interact too much (respectively too little) when the good contagion spreads more (respectively less) readily than the bad contagion, this assortative process helps to internalize the negative externalities generated by suboptimal levels of social interaction.

The continuous-strategy nature of our model of sociality also bears resemblance to recent work on the sustainable management of common-pool resources like fisheries, which has shown that social pressures can help to maintain sustainable levels of extraction effort as a stable social norm [39–41]. Further work on the two-contagion model can look to more deeply connect our social dilemma of sociality to the broader literature on evolutionary game theory and on game-theoretic models of host behavior and infectious disease. In particular, the nonlinear dependence of utility upon the composition of sociality strategies in the population and the endemic equilibria of the dimorphic contagion dynamics bears similarity to game-theoretic models of vaccination both in well-mixed and spatial populations [42–44] as well a models of nonlinear public goods games from evolutionary game theory [45, 46]. These game-theoretic scenarios arising from the collective behavior of an interacting population may be a more realistic representation of social dilemmas one encounters in everyday life than the stylized models of pairwise interactions such as the Prisoners’ Dilemma.

There are also many natural directions for future research regarding how social dilemmas of sociality may arise under more complicated models of disease dynamics or social network structure. While this paper restricts attention to a pair of simple contagions with bilinear incidence functions, many models of social transmission explore the spread of information via complex contagion [47, 48]. In addition, our assumption that the two contagions spread independently in the population could be relaxed to consider a variety of possible interacting dynamics between a pair of contagions, with examples ranging from the coupled spread of a disease and awareness of the disease outbreak [49] to the cultural transmission of a risky or careful behavior that impacts the likelihood of exposure to infectious disease [19]. Because both complex contagion and the superinfection of simple contagions often produce behaviors including bistability or Hopf bifuractions in disease dynamics [47, 50], it may be possible to observe more complicated evolutionary behaviors like evolutionary cycling in sociality strategies [51] if the good contagion features a sigmoidal incidence function or if infection with the good contagion impacts the ability to acquire or avoid the bad contagion. In addition, while sociality strategies are modeled here through the rate of well-mixed interactions that individuals have, it is also reasonable to consider how the benefits and costs of social interaction can impact how individuals choose neighbors in a network-structured population. Tools such as pair approximations [52, 53] or graphons [54–56] can be used to model the spread of couple contagions on graph-structured populations, and evolutionary questions could also explore the evolution of modular network structure in the presence of infectious disease [57–59].

In a recent paper, Ashby and Farine also study the evolutionary dynamics of sociality strategies depending upon the costs and benefits induced by the joint spread of a pair SIS contagions in a well-mixed population [29]. Focusing on the case of an infectious disease and an informational contagion, the authors assume that exposure to the informational contagion reduces the rate of death due to the infectious disease. Considering the effects of birth and both natural and disease-dependent death, Ashby and Farine apply an adaptive dynamics approach study pairwise invasibility of sociality strategies at demographic-epidemiological equilibria and to study the coevolution of host sociality strategy and virulence of the infectious disease. One advantage of their approach is that it allows explicit study of the ecoevolutionary dynamics for a specific tradeoff between the good and bad contagion. By contrast, our use of a simpler pair of SIS contagions and a flexible utility function measuring the costs and benefits of social interaction makes possible comparisons between socially-optimal and evolutionarily-stable sociality strategies. A shared feature of the two models is the key role played by the relative transmissibility of the two contagions (encoded in our model by the parameter *c*), which helps to shape the sociality strategies supported by the long-time evolutionary dynamics [29].

While many models for the evolution of social animal groups involve network or groups structures [25, 59–62], our model for the evolutionary dynamics of social strategies provides a simple, well-mixed baseline model for understanding the tension between the benefits and costs of informational and disease transmission via social interactions. This social dilemma of sociality motivates further study into mechanisms that can help promote socially-optimal rates of social interaction [37] and the establishment of social groups featuring long-time interaction. This misalignment between individual and collective interests is also reminiscent of the social dilemmas of social distancing explored in modern disease outbreaks [18, 63–66], in which individuals may choose to interact more than is collectively optimal in pursuit of economic or personal benefits of social interactions. Because infectious disease and social learning has been attributed as factors driving social evolution in settings ranging from the development of modular population structure [60] and division-of-labor [25] in social insects to the cultural evolution of collectivist social norms in human populations [67], we hope that this model highlights the challenges faced by individuals and populations in light of the inherent benefits and costs of social interaction.

## Supporting information

S1 Text Description of additional results for the two-type contagion and evolutionary dynamics (Section A), additional results for adaptive dynamics analysis (Section B), and derivation of utility functions using model of expected utility (Section C). (PDF)

S1 File Mathematica notebook for derivation in adaptive dynamics model featuring assortative interactions (description of this model and results of this of the analysis are provided in Section C of S1 Text). (PDF)
