# Supplementary material for: Social dilemmas of sociality due to beneficial and costly contagion
Source: PLoS Comput Biol. 2022 Nov 21;18(11):e1010670. doi: 10.1371/journal.pcbi.1010670 (PMC9721494; doi:10.1371/journal.pcbi.1010670)
Supplement: S1 Text — (PDF) [file pcbi.1010670.s001.pdf]

Appendix for "Social dilemmas of sociality due to beneficial and costly contagion"

Daniel B. Cooney[1,*], Dylan H. Morris[2,*], Simon A. Levin[3], Daniel I. Rubenstein[3], and Pawel Romanczuk[4,5,6]

[1]Department of Mathematics, University of Pennsylvania, Philadelphia, USA
[2]Department of Ecology & Evolutionary Biology, University of California, Los Angeles, USA
[3]Department of Ecology & Evolution Biology, Princeton University, Princeton, USA
[4]Institute for Theoretical Biology, Department of Biology, Humboldt-Universität zu Berlin, 10115 Berlin, Germany
[5]Bernstein Center for Computational Neuroscience Berlin, 10115 Berlin, Germany
[6]Science of Intelligence, Research Cluster of Excellence, Marchstr. 23, 10587 Berlin, Germany, https://www.scienceofintelligence.de
[*]These authors contributed equally and should be considered joint first authors; correspondence to dbcooney@sas.upenn.edu, dylan@dylanhmorris.com

August 4, 2022

Appendices

Contents

A Properties of Contagion Dynamics and Evolutionary Dynamics in Dimorphic Populations 2
- A.1 Dimorphic Contact Rates . . . 2
- A.2 Basic Reproduction Number for Dimorphic Contagion Dynamics . . . 3
- A.3 Analysis of Replicator Equation for Cobb-Douglas Utility . . . 6

B Additional Analysis of the Adaptive Dynamics Limit 8
- B.1 Derivation of Socially Optimal Level of Sociality for Cobb-Douglas Utility . . . 9
- B.2 Quantifying the Social Dilemma via the Price of Anarchy . . . 10
- B.3 Evolutionary and Convergence Stability for Cobb-Douglas Utility . . . 11
- B.4 Linear Utility Function . . . 13
- B.5 Constant Elasticity of Substitution (CES) Utility . . . 15
- B.6 Assortative Interactions and Mitigating the Social Dilemma . . . 21

C Microfoundation for Population Utility Function 23

A Properties of Contagion Dynamics and Evolutionary Dynamics in Dimorphic Populations

In this section, we discuss additional properties of the contagion and evolutionary dynamics in the case of two competing sociality strategies. In Section A.1, we derive the contact rates experienced in the two-type contagion dynamics in a population under various possible compositions of the population and the possible sociality strategies of the resident and mutant type. In Section A.2, we then characterize the basic reproduction number of the two-strategy contagion dynamics, exploring how the pair of sociality strategies can determine whether the long-time contagion dynamics will converge to a contagion-free state or to a unique endemic equilibrium. Finally, in Section A.3, we study the behavior of the replicator equation for the evolutionary dynamics for a given pair of sociality strategies. We provide a proof of Proposition 1, showing that the qualitative behavior of the long-time composition of resident and mutant sociality strategies can be determined by analyzing the ability of each strategy to invade the other when initially rare in the population.

A.1 Dimorphic Contact Rates

To explore the contagion dynamics in a population with two sociality strategies, we start with a density-dependent description of the population. We assume that the total population has size N, of whom $N_m = fN$ follow a mutant strategy making σ_m social contacts per unit time and $N_r = (1-f)N$ follow a resident strategy with an analogous social contact rate of σ_m. Focusing first on a single contagion, we describe for, each contagion $x \in \{g, b\}$, the disease states in the resident population by the densities $\mathbb{I}_r^{(x)}$ and $\mathbb{S}_r^{(x)} = N_r - \mathbb{I}_r^{(x)}$ and in the mutant population by $\mathbb{I}_m^{(x)}$ and $\mathbb{S}_m^{(x)} = N_m - \mathbb{I}_m^{(x)}$. Under these sociality strategies, infectious resident and mutants individuals collectively have $\sigma_r \mathbb{I}_r^{(x)}$ and $\sigma_m \mathbb{I}_m^{(x)}$ social contacts per unit time, while the rate of social contacts for the whole population is $\sigma_m f N + \sigma_r (1-f) N$. Therefore the probability that an individuals meets an infectious individual in a given social interaction can be written as

$$\frac{\sigma_m \mathbb{I}_m^{(x)} + \sigma_r \mathbb{I}_r^{(x)}}{N\left(\sigma_m f N + \sigma_r (1-f)\right)}.$$

Further assuming that social interactions produce infection with probability p_x and that infectious individuals recover with rate γ_x, the density of infectious mutant and resident individuals evolve according to the system of ODEs

$$\frac{\mathrm{d}\mathbb{I}_m^{(x)}}{\mathrm{d}t} = \sigma_m p_x \left[\frac{\sigma_m \mathbb{I}_m^{(x)} + \sigma_r \mathbb{I}_r^{(x)}}{N\left(\sigma_m f + \sigma_r (1-f)\right)}\right] \mathbb{S}_m^{(x)} - \gamma_x \mathbb{I}_m^{(x)} \tag{S1.A.1a}$$

$$\frac{\mathrm{d}\mathbb{I}_r^{(x)}}{\mathrm{d}t} = \sigma_r p_x \left[\frac{\sigma_m \mathbb{I}_m^{(x)} + \sigma_r \mathbb{I}_r^{(x)}}{N\left(\sigma_m f + \sigma_r (1-f)\right)}\right] \mathbb{S}_r^{(x)} - \gamma_x \mathbb{I}_r^{(x)} \tag{S1.A.1b}$$

Using the reproduction numbers $\mathcal{R}_m^{(x)} = \dfrac{\sigma_m p_x}{\gamma}$ and $\mathcal{R}_r^{(g)} = \dfrac{\sigma_r p}{\gamma_x}$ and denoting the fractions $I_x^{(x)} := \dfrac{\mathbb{I}_y^{(x)}}{N_y}$ and $S_y^{(x)} := \dfrac{\mathbb{S}_y^{(x)}}{N_y}$ of infectious and susceptible individuals in the populations $y \in \{r, m\}$, we

can further obtain the following frequency-dependent analogue of Equation (S1.A.1)

$$\frac{1}{\gamma_x}\frac{\mathrm{d}I_m^{(x)}}{\mathrm{d}t} = \mathcal{R}_m^{(g)}\Big[\frac{\mathcal{R}_m^{(g)} f I_m^{(x)} + \mathcal{R}_r^{(g)}(1-f)I_r^{(x)}}{\mathcal{R}_m^{(x)} f + \mathcal{R}_r^{(x)}(1-f)}\Big]S_m^{(x)} - I_m^{(x)} \tag{S1.A.2a}$$

$$\frac{1}{\gamma_x}\frac{\mathrm{d}I_r^{(x)}}{\mathrm{d}t} = \mathcal{R}_r^{(x)}\Big[\frac{\mathcal{R}_m^{(x)} f I_m^{(x)} + \mathcal{R}_r^{(x)}(1-r)I_r^{(x)}}{\mathcal{R}_m^{(x)} f + \mathcal{R}_r^{(x)}(1-f)}\Big]S_r^{(x)} - I_r^{(x)}. \tag{S1.A.2b}$$

Finally, rescaling time and using the fact that $S_y^{(x)} = 1 - I_y^{(x)}$ allows us to obtain the frequency-depending dimorphic contagion dynamics of Equations (9a) and (9b) for the good contagion and an analogous system for the bad contagion.

A.2 Basic Reproduction Number for Dimorphic Contagion Dynamics

To gain insight into the contagion process with two levels of sociality and to assess the stability of the disease-free equilibrium, we use the next-generation matrix method of Diekmann and colleagues [1] and van den Driessche and Watmough [2] to find the overall basic reproduction number of the contagion process. For this analysis, we multiply both sides of Equations (S1.A.2a) and (S1.A.2b) by γ_x, which allows to rewrite the coupled contagion dynamics for the resident and mutant populations as

$$\begin{aligned}\frac{\mathrm{d}I_m}{\mathrm{d}t} &= \mathcal{F}_m(I_m, I_r) - \mathcal{V}_m(I_m, I_r)\\ \frac{\mathrm{d}I_r}{\mathrm{d}t} &= \mathcal{F}_r(I_m, I_r) - \mathcal{V}_r(I_m, I_r),\end{aligned} \tag{S1.A.3}$$

where

$$\mathcal{F}_m(I_m^{(x)}, I_r^{(x)}) = \gamma_x\left\{\mathcal{R}_m^{(x)}\Big[\frac{\mathcal{R}_r^{(x)}(1-f)I_r^{(x)} + \mathcal{R}_m^{(x)} f I_m^{(x)}}{\mathcal{R}_r^{(x)}(1-f) + \mathcal{R}_m^{(x)} f}\Big](1 - I_m^{(x)})\right\}$$

$$\mathcal{F}_r(I_m^{(x)}, I_r^{(x)}) = \gamma_x\left\{\mathcal{R}_r^{(x)}\Big[\frac{\mathcal{R}_r^{(x)}(1-f)I_r^{(x)} + \mathcal{R}_m^{(x)} f I_m^{(x)}}{\mathcal{R}_r^{(x)}(1-f) + \mathcal{R}_m^{(x)} f}\Big](1 - I_r^{(x)})\right\}$$

$$\mathcal{V}_m(I_m^{(x)}, I_r^{(x)}) = \gamma_x I_m^{(x)}$$

$$\mathcal{V}_r(I_m^{(x)}, I_r^{(x)}) = \gamma_x I_r^{(x)}$$

and $\mathcal{F}_m$ and $\mathcal{F}_r$ represent rates of new infections, while V_m and V_r represent rates of recoveries.

The next generation matrix is the matrix FV^{-1} where the matrices F and V are given by

$$F = \left.\begin{pmatrix} \dfrac{\partial \mathcal{F}_m}{\partial I_m^{(x)}} & \dfrac{\partial \mathcal{F}_m}{\partial I_r^{(x)}} \\ \dfrac{\partial \mathcal{F}_r}{\partial I_m^{(x)}} & \dfrac{\partial \mathcal{F}_r}{\partial I_r^{(x)}} \end{pmatrix}\right|_{(I_m^{(x)}, I_r^{(x)})=(0,0)} = \begin{pmatrix} \dfrac{\gamma_x f\left(\mathcal{R}_m^{(x)}\right)^2}{\hat{\mathcal{R}}^{(x)}} & \dfrac{\gamma_x(1-f)\mathcal{R}_m^{(x)}\mathcal{R}_r^{(x)}}{\hat{\mathcal{R}}^{(x)}} \\ \dfrac{\gamma_x f\mathcal{R}_m^{(x)}\mathcal{R}_r^{(x)}}{\hat{\mathcal{R}}^{(x)}} & \dfrac{\gamma_x(1-f)\left(\mathcal{R}_r^{(x)}\right)^2}{\hat{\mathcal{R}}^{(x)}} \end{pmatrix} \tag{S1.A.4}$$

$$V = \begin{pmatrix} \frac{\partial V_m}{\partial I_m^{(x)}} & \frac{\partial V_m}{\partial I_r^{(x)}} \\ \\ \frac{\partial V_r}{\partial I_m^{(x)}} & \frac{\partial V_r}{\partial I_r^{(x)}} \end{pmatrix}_{(I_m^{(x)}, I_r^{(x)})=(0,0)} = \begin{pmatrix} \gamma_x & 0 \\ \\ 0 & \gamma_x \end{pmatrix}, \tag{S1.A.5}$$

with $\hat{\mathcal{R}}^{(x)} := \mathcal{R}_m^{(x)}(1-f) + \mathcal{R}_m^{(x)} f$. We may then compute the next generation matrix, which takes the following form:

$$FV^{-1} = \begin{pmatrix} \frac{\gamma_x f \left(\mathcal{R}_m^{(x)}\right)^2}{\hat{\mathcal{R}}^{(x)}} & \frac{\gamma_x (1-f) \mathcal{R}_m^{(x)} \mathcal{R}_r^{(x)}}{\hat{\mathcal{R}}^{(x)}} \\ \\ \frac{\gamma_x f \mathcal{R}_m^{(x)} \mathcal{R}_r^{(x)}}{\hat{\mathcal{R}}^{(x)}} & \frac{\gamma_x (1-f) \left(\mathcal{R}_r^{(x)}\right)^2}{\hat{\mathcal{R}}^{(x)}} \end{pmatrix} \begin{pmatrix} \frac{1}{\gamma_x} & 0 \\ 0 & \frac{1}{\gamma_x} \end{pmatrix} = \begin{pmatrix} \frac{f \left(\mathcal{R}_m^{(x)}\right)^2}{\hat{\mathcal{R}}^{(x)}} & \frac{(1-f) \mathcal{R}_m^{(x)} \mathcal{R}_r^{(x)}}{\hat{\mathcal{R}}^{(x)}} \\ \\ \frac{f \mathcal{R}_m^{(x)} \mathcal{R}_r^{(x)}}{\hat{\mathcal{R}}^{(x)}} & \frac{(1-f) \left(\mathcal{R}_r^{(x)}\right)^2}{\hat{\mathcal{R}}^{(x)}} \end{pmatrix} \tag{S1.A.6}$$

We note that, in this case, our next generation matrix given by Equation S1.A.6 is a rank one matrix, as the two columns are scalar multiples of each other. The ratio between the first column and the second column is $\frac{f \mathcal{R}_m^{(x)}}{(1-f)\mathcal{R}_r^{(x)}}$, which is a consequence of our assumptions that the difference between the resident and mutant strategies is their relative rate of social contacts σ_m and σ_r and that each social interaction follows an unbiased sampling of interaction partners from the pool of available contacts (with probabilities of interaction between sociality strategies determined by the relative contact rates and relative abundances of the resident and mutant strategies).

The quantity $\mathcal{R}_{\text{net}}^{(x)}$ given by the spectral radius of the next generation matrix is the overall basic reproduction number of the contagion process. When $\mathcal{R}_{\text{net}}^{(x)} > 1$, the contagion-free equilibrium is unstable, and the results of Hethcote and Yorke [3] then imply that there exists a unique stable endemic equilibrium. When $\mathcal{R}_{\text{net}}^{(x)} < 1$, the contagion-free equilibrium is stable, and as shown by Hethcote and Yorke [3], this implies that no endemic equilibrium exists.

The eigenvalues of F are 0 and $\frac{f \left(\mathcal{R}_m^{(x)}\right)^2 + (1-f)\left(\mathcal{R}_r^{(x)}\right)^2}{f \mathcal{R}_m^{(x)} + (1-f)\mathcal{R}_r^{(x)}}$. Since the second eigenvalue is always non-negative for $0 \leq f \leq 1$ and $\mathcal{R}_m^{(x)}, \mathcal{R}_r^{(x)} \geq 0$ (with at least one greater than 0), it is the spectral radius, so

$$\mathcal{R}_{\text{net}} = \frac{f \left(\mathcal{R}_m^{(x)}\right)^2 + (1-f)\left(\mathcal{R}_r^{(x)}\right)^2}{f \mathcal{R}_m^{(x)} + (1-f)\mathcal{R}_r^{(x)}}. \tag{S1.A.7}$$

$\mathcal{R}_{\text{net}}$is the ratio of the second moment to the first moment for the subpopulation reproduction numbers:

$$\mathcal{R}_{\text{net}}^{(x)} = \frac{E(\mathcal{R}^2)}{E(\mathcal{R})}$$

This formula is analogous to the basic reproduction number $\mathcal{R}_{\text{RG}}$ studied for SIS models with interactions taking place on random graphs with heterogeneous distributions over individual degree

k [4–8], given by

$$\mathcal{R}_{\mathrm{RG}} = \frac{\beta}{\gamma}\frac{\langle k\rangle}{\langle k^2\rangle}. \tag{S1.A.8}$$

From this correspondence, we can see that our results for the two-group SIS model with different rates of interaction for the resident and mutant groups can be reinterpreted as a model for interactions occurring on a stochastic block model with two groups of individuals having different linking probabilities.

When $0 < f < 1$, the condition $\mathcal{R}_{\mathrm{net}}^{(x)} < 1$ for stability of the contagion-free equilibrium can be rewritten as

$$4f\left(\mathcal{R}_m^{(x)} - \frac{1}{2}\right)^2 + 4(1-f)\left(\mathcal{R}_r^{(x)} - \frac{1}{2}\right)^2 < 1. \tag{S1.A.9}$$

This stability boundary characterizes an ellipse in the $\mathcal{R}_r^{(x)}, \mathcal{R}_m^{(x)}$ plane centered at $(\mathcal{R}_r^{(x)}, \mathcal{R}_m^{(x)}) = (\frac{1}{2}, \frac{1}{2})$ with an $\mathcal{R}_m^{(x)}$-axis length of $\frac{1}{\sqrt{f}}$ and an $\mathcal{R}_r^{(x)}$-axis length of $\frac{1}{\sqrt{1-f}}$, which we illustrate in Figure 1.

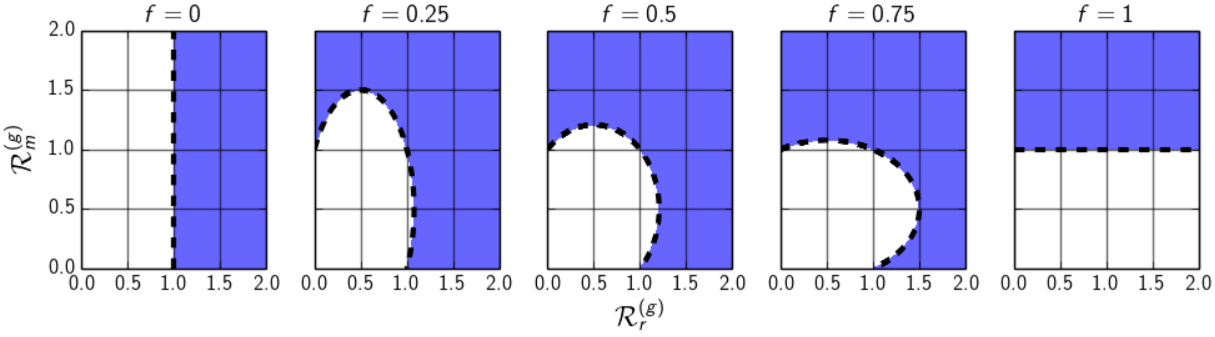

Figure 1: Regions for which the net reproduction number $\mathcal{R}_{\mathrm{net}}^{(x)} > 1$ (blue) for various resident and mutant reproduction numbers $\mathcal{R}_r^{(x)}$ and $\mathcal{R}_m^{(x)}$, for various fractions of mutant population f.

Remark A.1. We can use Equation (S1.A.7) to deduce the following properties of $\mathcal{R}_{\mathrm{net}}$.

- In the limiting cases of $f = 0$ and $f = 1$, the net reproduction number reduces to $\mathcal{R}_{\mathrm{net}}^{(x)} = \mathcal{R}_r^{(x)}$ and $\mathcal{R}_{\mathrm{net}}^{(x)} = \mathcal{R}_r^{(x)}$, respectively.
- If $\mathcal{R}_m^{(x)}, \mathcal{R}_r^{(x)} > 1$, then $\mathcal{R}_{\mathrm{net}}^{(x)} > 1$ and contagion-free equilibrium is unstable.
- If $0 \leq \mathcal{R}_m^{(x)}, \mathcal{R}_r^{(x)} < 1$, then $\mathcal{R}_{\mathrm{net}}^{(x)} \leq 1$ and the contagion-free equilibrium is stable.
- If $\mathcal{R}_r^{(x)} = 1$, then the signs of $\mathcal{R}_{\mathrm{net}}^{(x)} - 1$ and $\mathcal{R}_m^{(x)} - 1$ agree. In other words, if the fully-resident population has a marginally stable contagion-free equilibrium, than the stability of the contagion-free equilibrium under the dimorphic dynamics is determined by the sign of $\mathcal{R}_m^{(x)} - 1$.
- If $\mathcal{R}_r^{(x)} < 1$, then $\mathcal{R}_{\mathrm{net}}^{(x)} > 1$ provided that

$$\mathcal{R}_m^{(x)} > \frac{1}{2} + \sqrt{\frac{1}{4} + \left(\frac{1-f}{f}\right)\mathcal{R}_r^{(x)}\left(1 - \mathcal{R}_r^{(x)}\right)}.$$

This threshold is maximized when $\mathcal{R}_r^{(x)} = \frac{1}{2}$.

A.3 Analysis of Replicator Equation for Cobb-Douglas Utility

Here we consider the long-time behavior of the replicator equation for pairwise competition for sociality strategies from the Replicator Dynamics Section in the case of Cobb-Douglas utility. The main output of this section is the proof of Proposition 1, which characterizes the possible long-time outcomes for the replicator equation in the case of Cobb-Douglas utility.

To understand the stability of equilibria of the replicator equation with Cobb-Douglas utility, it is convenient to study a following modified form of Equation (12) based upon log-transformed utilities. In this case, given resident and mutant sociality strategies featuring good contagion reproductive numbers $\mathcal{R}_m^{(g)}$ and $\mathcal{R}_m^{(g)}$, the fraction f of individuals with the mutant strategy evolves according to

$$\frac{\mathrm{d}f}{\mathrm{d}t} = f(1-f)\left[\log\left(U_m(f)\right) - \log\left(U_r(f)\right)\right], \tag{S1.A.10}$$

where

$$\begin{aligned} \log\left(U_m(f)\right) &= \alpha \log\left[U\left(\hat{I}_m^{(g)}(\mathcal{R}_m^{(g)}, \mathcal{R}_r^{(g)}, f), \hat{S}_m^b(\mathcal{R}_m^{(g)}, \mathcal{R}_r^{(g)}, f)\right)\right] \\ \log\left(U_r(f)\right) &= \alpha \log\left[U\left(\hat{I}_r^{(g)}(\mathcal{R}_m^{(g)}, \mathcal{R}_r^{(g)}, f), \hat{S}_r^b(\mathcal{R}_m^{(g)}, \mathcal{R}_r^{(g)}, f)\right)\right] \end{aligned} \tag{S1.A.11}$$

and the quantities $\hat{I}_y^x(\cdot)$ and $\hat{S}_y^x(\cdot)$ are equilibrium values of the dimorphic contagion dynamics as describe by Equations (9a) and (9b).

Solutions to Equation (S1.A.10) will have the same long-time behavior as solutions to Equation (12). Therefore we can characterize the long-time behavior of Equation (12) using a useful monotonicity property for the relative log-utility as a function of the fraction of mutants f. Next, we recall the statement of Proposition 1, in which we show that that the only possible long-time behaviors for the replicator equation under Cobb-Douglas utility are dominance of the mutant strategy, dominance of the resident strategy, and coexistence of the two strategies at a unique interior equilibrium. Furthermore, the behavior for a given pair of strategies can be determined by evaluating the utilities of the resident and mutant strategies at the endpoints $f = 0$ and $f = 1$ in which the population has an all-resident or all-mutant composition, so dominance under pairwise invasibility analysis correspond to dominance under competition at relative frequencies of the mutant and resident at all $f \in [0, 1]$.

Proposition 1. *Suppose that the resident and mutant types have sociality strategies featuring reproduction numbers $\mathcal{R}_r^{(g)} \geq 1$ and $\mathcal{R}_m^{(g)} \geq 1$ for the good contagion, with $\mathcal{R}_r^{(g)} \neq \mathcal{R}_m^{(g)}$ and at least one of these reproduction numbers strictly greater than 1. Then, for any $c > 0$ and for any resident and mutant types with reproduction numbers $\mathcal{R}_r^{(b)} = c\mathcal{R}_r^{(g)}$ and $\mathcal{R}_m^{(b)} = c\mathcal{R}_m^{(g)}$ for the bad contagion, the difference of Cobb-Douglas log-utilities $\log\left[U_m(f)\right] - \log\left[U_r(f)\right]$ is a decreasing function of f. As a consequence, the long-time behavior can be determined by the relative values of $U_m(f)$ and $U_r(f)$ at the endpoints $f = 0$ and $f = 1$. The three possible cases are the following:*

- *$U_m(0) > U_r(0)$ and $U_m(1) > U_r(1)$: $f = 1$ is globally stable and the mutant will fix in the population.*
- *$U_m(0) < U_r(0)$ and $U_m(1) < U_r(1)$: $f = 0$ is globally stable and the resident will fix in the population.*

- $U_m(0) > U_r(0)$ *and* $U_m(1) < U_r(1)$: *There exists a unique interior equilibrium* $\hat{f} \in (0,1)$ *that is globally stable, and mutant and resident will coexist in the long-time population.*

We note that the fact that the difference in log-utilities is decreasing rules out the possibility that $U_m(0) > U_r(0)$ and $U_m(1) < U_r(0)$, and therefore it is impossible for the replicator dynamics to achieve bistability of a full-resident and full-mutant population under Cobb-Douglas utility.

Proof. It is useful to write the dimorphic SIS dynamics of Equations (9a) and (9b) in terms of the transmission function $\Lambda_x(I_m^{(x)}, I_r^{(x)})$:

$$\Lambda_x(I_m^{(x)}, I_r^{(x)}) = \frac{\mathcal{R}_m^{(x)} f I_m^{(x)} + \mathcal{R}_r^{(x)}(1-f) I_r^{(x)}}{\mathcal{R}_m^{(x)} f + \mathcal{R}_r^{(x)}(1-f)} \geq 0. \tag{S1.A.12}$$

We can use this transmission function to find the implicit expressions for nonzero contagion equilibria

$$\begin{aligned} \hat{I}_m^{(x)} &= \frac{\mathcal{R}_m^{(x)} \Lambda_x(\hat{I}_m^{(x)}, \hat{I}_r^{(x)})}{1 + \mathcal{R}_m^{(x)} \Lambda_x(\hat{I}_m^{(x)}, \hat{I}_r^{(x)})} &&= 1 - \frac{1}{1 + \mathcal{R}_m^{(x)} \Lambda_x(\hat{I}_m^{(x)}, \hat{I}_r^{(x)})} \\ \hat{I}_r^{(x)} &= \frac{\mathcal{R}_r^{(x)} \Lambda_x(\hat{I}_m^{(x)}, \hat{I}_r^{(x)})}{1 + \mathcal{R}_r^{(x)} \Lambda_x(\hat{I}_m^{(x)}, \hat{I}_r^{(x)})} &&= 1 - \frac{1}{1 + \mathcal{R}_r^{(x)} \Lambda_x(\hat{I}_m^{(x)}, \hat{I}_r^{(x)})} \end{aligned}. \tag{S1.A.13}$$

From our assumptions on the reproduction numbers $\mathcal{R}_m^{(g)}$ and $\mathcal{R}_r^{(g)}$, we know that the good contagion will be present at equilibrium for both resident and mutant: $\hat{I}_m^{(g)} > 0$ and $\hat{I}_r^{(g)} > 0$. For the bad contagion, either the equilibrium fraction of susceptible individuals is 1 for both groups (when $\mathcal{R}_{\text{net}}^{(g)} < \frac{1}{c}$)) or it can expressed in terms of Equation (S1.A.13) via $(\hat{S}_r^{(b)}, \hat{S}_m^{(b)}) = (1 - \hat{I}_r^{(b)}, 1 - \hat{I}_m^{(b)})$ (when $\mathcal{R}_{\text{net}}^{(g)} \geq \frac{1}{c}$. Using these properties of the equilibria for both contagions, we see that the difference between Cobb-Douglas log-utilities for the mutant and resident populations has the following piecewise characterization

$$\log(U_m(f)) - \log(U_r(f)) = \begin{cases} \alpha \log\left(\frac{\hat{I}_m^{(g)}}{\hat{I}_r^{(g)}}\right) & : \mathcal{R}_{\text{net}}^{(g)} < \frac{1}{c} \\ \alpha \log\left(\frac{\hat{I}_m^{(g)}}{\hat{I}_r^{(g)}}\right) + (1-\alpha)\log\left(\frac{\hat{S}_m^{(b)}}{\hat{S}_r^{(b)}}\right) & : \mathcal{R}_{\text{net}}^{(g)} \geq \frac{1}{c} \end{cases} \tag{S1.A.14}$$

Now we look to study how the difference in log-utilities changes with mutant fraction f. Using Equation (S1.A.12), we compute that, for each contagion $x \in \{g, b\}$,

$$\frac{\partial \Lambda_x}{\partial f} = \frac{\mathcal{R}_m^{(x)} \mathcal{R}_r^{(x)} (\hat{I}_m^{(x)} - \hat{I}_r^{(x)})}{\left(f\mathcal{R}_m^{(x)} + (1-f)\mathcal{R}_r^{(x)}\right)^2} \tag{S1.A.15}$$

Using Equation (S1.A.13), the ratio of endemic equilibrium levels for the good contagion is given by

$$\zeta_g := \log\left(\frac{\hat{I}_m^{(g)}}{\hat{I}_r^{(g)}}\right) = \log\left[\left(\frac{\mathcal{R}_m^{(g)}}{\mathcal{R}_r^{(g)}}\right) \frac{1 + \mathcal{R}_r^{(g)} \Lambda_g}{1 + \mathcal{R}_m^{(g)} \Lambda_g}\right]. \tag{S1.A.16}$$

We can then differentiate to see that

$$\frac{\partial \zeta_g}{\partial f} = \frac{\mathcal{R}_r^{(g)}}{1+\mathcal{R}_r^{(g)}\Lambda_g}\frac{\partial \Lambda}{\partial f} - \frac{\mathcal{R}_m^{(g)}}{1+\mathcal{R}_m^{(g)}\Lambda_g}\frac{\partial \Lambda}{\partial f} = \frac{\partial \Lambda_g}{\partial f}\left(\frac{\mathcal{R}_r^{(g)}}{1+\mathcal{R}_r^{(g)}\Lambda_g} - \frac{\mathcal{R}_m^{(g)}}{1+\mathcal{R}_m^{(g)}\Lambda_g}\right) = \frac{\partial \Lambda_g}{\partial f}\frac{1}{\Lambda_g}(\hat{I}_r^{(g)} - \hat{I}_m^{(g)}) < 0, \tag{S1.A.17}$$

where we deduce the direction of the inequality by using Equation (S1.A.15) to note that $\frac{\partial \Lambda_x}{\partial f}$ and $\hat{I}_r^{(x)} - \hat{I}_m^{(x)}$ always have opposite signs (for both the good and bad contagion) and note that the inequality is strict because of the assumption that $\mathcal{R}_m^{(g)} \neq \mathcal{R}_r^{(g)}$.

Similarly, we can use Equation (S1.A.13) to compute the log-ratio for the susceptible fractions at the nontrivial endemic equilibrium for the bad contagion as

$$\nu_b := \log\left(\frac{\hat{S}_m^{(b)}}{\hat{S}_r^{(b)}}\right) = \log\left(\frac{1-\hat{I}_m^{(b)}}{1-\hat{I}_r^{(b)}}\right) = \log\left(\frac{1+c\mathcal{R}_r^{(g)}\Lambda_b}{1+c\mathcal{R}_m^{(g)}\Lambda_b}\right). \tag{S1.A.18}$$

Then we can differentiate to see that

$$\frac{\partial \nu_b}{\partial f} = \frac{\partial \Lambda_b}{\partial f}\left(\frac{c\mathcal{R}_r^{(g)}}{1+c\mathcal{R}_r^{(g)}\Lambda_b} - \frac{c\mathcal{R}_m^{(g)}}{1+c\mathcal{R}_m^{(g)}\Lambda_b}\right) = \frac{\partial \Lambda_b}{\partial f}\frac{1}{\Lambda_b}(\hat{I}_r^{(b)} - \hat{I}_m^{(b)}) < 0. \tag{S1.A.19}$$

Differentiating Equation (S1.A.14) with respect to f, we see from Equations (S1.A.17) and (S1.A.19) that

$$\frac{\partial}{\partial f}\left[\log(U_m(f)) - \log(U_f(f))\right] = \left\{\begin{array}{cc} \alpha\frac{\partial \zeta_g}{\partial f} & : \mathcal{R}^g_{\text{net}} < \frac{1}{c} \\ \alpha\frac{\partial \zeta_g}{\partial f} + (1-\alpha)\frac{\partial \nu_b}{\partial f} & : \mathcal{R}^g_{\text{net}} \geq \frac{1}{c} \end{array}\right\} < 0. \tag{S1.A.20}$$

Therefore we see that the difference in log-utilities is a differentiable, monotonically decreasing function of the mutant fraction f, implying that the there will be a unique stable equilibrium of the replicator dynamics of Equation (12) and (S1.A.10) and that the trifold alternative described above holds for the longtime behavior of these replicator equations. □

B Additional Analysis of the Adaptive Dynamics Limit

In this section, we further explore our adaptive dynamics analysis of the social dilemma of sociality. In Section B.1, we provide the derivation of the formula for the socially optimum level of social interaction for the case of Cobb-Douglas utility. In Section B.2, we the Price of Anarchy to quantify the gap between social utility for populations following the socially-optimal and evolutionarily-stable sociality strategies. In Section B.3, we further characterize the evolutionary and convergence stability of sociality strategies under adaptive dynamics, showing that the sociality strategy corresponding to $\mathcal{R}_{\text{ESS}}^{(g)}$ is the unique evolutionarily-stable and convergence stable strategy for the Cobb-Douglas utility. In Section B.4), we analyze the socially-optimal and evolutionarily-stable sociality strategies in the case of a linear utility function, showing that it possible to achieve infinite sociality levels and bistable evolutionary dynamics in this case. In Section B.5, we perform a similar analysis for the Constant Elasticity of Substitution (CES) family of utility functions, show, for the case in which the bad contagion spreads more readily than the good contagion ($c > 1$), that there is broad range of utility parameters for which the evolutionary dynamics achieve bistability between an ESS featuring spread of both contagions and an ESS at $\frac{1}{c}$ in which both contagions are eliminated. Finally,

in Section B.6, we consider the role of assortative interactions in which individuals preferentially interact with individuals with the same sociality strategy. This assortative mechanism helps to internalize negative externalities generated by subpoptimal rates of social interaction, and we show that assortment helps to mitigate the social dilemma and produces evolutionarily-stable levels of social interaction that are closer to the social optimum.

B.1 Derivation of Socially Optimal Level of Sociality for Cobb-Douglas Utility

For completeness, we provide in this section the derivation of the socially optimum interaction rate $\mathcal{R}^{(g)}_{\text{opt}}$ for the case of Cobb-Douglas utility. The main effort involved in this derivation is checking the conditions under which the utility for the population is maximized by a level of social interaction at which the bad contagion is unable to spread $\frac{1}{c}$ or at a level of social interaction at which both the good and bad contagion spread in the population.

Since $U(\mathcal{R}^{(g)}) = 0$ for $\mathcal{R}^{(g)} \leq 1$, it suffices to optimize U on the interval $(1, \infty)$. We can equivalently maximize $\log[U(\mathcal{R}^{(g)})]$; differentiating that log-utility yields

$$\frac{\partial}{\partial \mathcal{R}^{(g)}} \log[U(\mathcal{R}^{(g)})] = \begin{cases} \dfrac{\alpha}{\mathcal{R}^{(g)}\left(\mathcal{R}^{(g)} - 1\right)} & : 1 \leq \mathcal{R}^{(g)} < \dfrac{1}{c} \\ \dfrac{1}{\mathcal{R}^{(g)}}\left[\dfrac{\alpha}{\mathcal{R}^{(g)} - 1} - (1-\alpha)\right] & : \mathcal{R}^{(g)} > \dfrac{1}{c} \end{cases}. \tag{S1.B.1}$$

The log-utility is not differentiable at $\mathcal{R}^{(g)} = \frac{1}{c}$, but it has left and right derivatives at $\frac{1}{c}$ given by the expressions from the cases $\mathcal{R}^{(g)} < \frac{1}{c}$ and $\mathcal{R}^{(g)} > \frac{1}{c}$, respectively.

From Equation (S1.B.1), we see that the log-utility is increasing for $\mathcal{R}^{(g)} < \frac{1}{c}$, so its maximizer must reside in $[\frac{1}{c}, \infty)$. We also see from (S1.B.1) that the log-utility has a local maximum at $(\mathcal{R}^{(g)})^* = \frac{1}{1-\alpha}$ provided that $(\mathcal{R}^{(g)})^* > \frac{1}{c}$, a condition that is satisfied when $c > 1-\alpha$. Furthermore, in this case, log-utility is increasing for $\mathcal{R}^{(g)} \in [\frac{1}{c}, (\mathcal{R}^{(g)})^*)$ and decreasing for $\mathcal{R}^{(g)} > (\mathcal{R}^{(g)})^*$, so $(\mathcal{R}^{(g)})^*$ maximizes the log-utility when $c > 1 - \alpha$.

For the alternative case in which $c \leq 1 - \alpha$, we can see from Equation (S1.B.1) that, for $c < 1$ and $\mathcal{R}^{(g)} > \frac{1}{c}$,

$$\begin{aligned} \frac{\partial \log[U(\mathcal{R}^{(g)})]}{\partial \mathcal{R}^{(g)}} &\leq c\left[\frac{\alpha}{c^{-1} - 1} - (1-\alpha)\right] = \left(\frac{c}{1-c}\right)\left[\alpha c - (1-c)(1-\alpha)\right] \\ &= \left(\frac{c}{1-c}\right)\left[\alpha + c - 1\right] < 0, \end{aligned} \tag{S1.B.2}$$

and therefore we can combine this with the fact that log-utility is increasing for $\mathcal{R}^{(g)} < \frac{1}{c}$ to deduce that the log-utility is maximized at $\mathcal{R}^{(g)} = \frac{1}{c}$ when $c \leq 1 - \alpha$. To summarize, under the Cobb-Douglas utility, the socially-optimal level of sociality $\mathcal{R}^{(g)}_{\text{opt}}$ is given by

$$\mathcal{R}^{(g)}_{\text{opt}} = \max\left(\frac{1}{c}, \frac{1}{1-\alpha}\right). \tag{S1.B.3}$$

B.2 Quantifying the Social Dilemma via the Price of Anarchy

In addition to our analysis considered in Adaptive Dynamics, we can compare the evolutionarily-stable and socially-optimal outcomes based upon the utility levels achieved by monomorphic populations featuring each strategy. In Figure 2, we plot the Cobb-Douglas utilities obtained by populations following strategies $\mathcal{R}_{\mathrm{ESS}}^{(g)}$ and $\mathcal{R}_{\mathrm{opt}}^{(g)}$ given in Table 1 for the same values of α and c as in Figure 4c. Notably, the utility of the ESS strategy when $c = 2$ is increasing in α, while the utility of the social optimum for $c = 2$ and both strategies for $c = \frac{1}{2}$ achieve a minimal utility for intermediate values of α. This means that, for the evolutionary dynamics with $c = 2$, caring more about exposure to good contagion produces a better outcome long-run outcome even though the bad contagion spreads more readily than the good contagion.

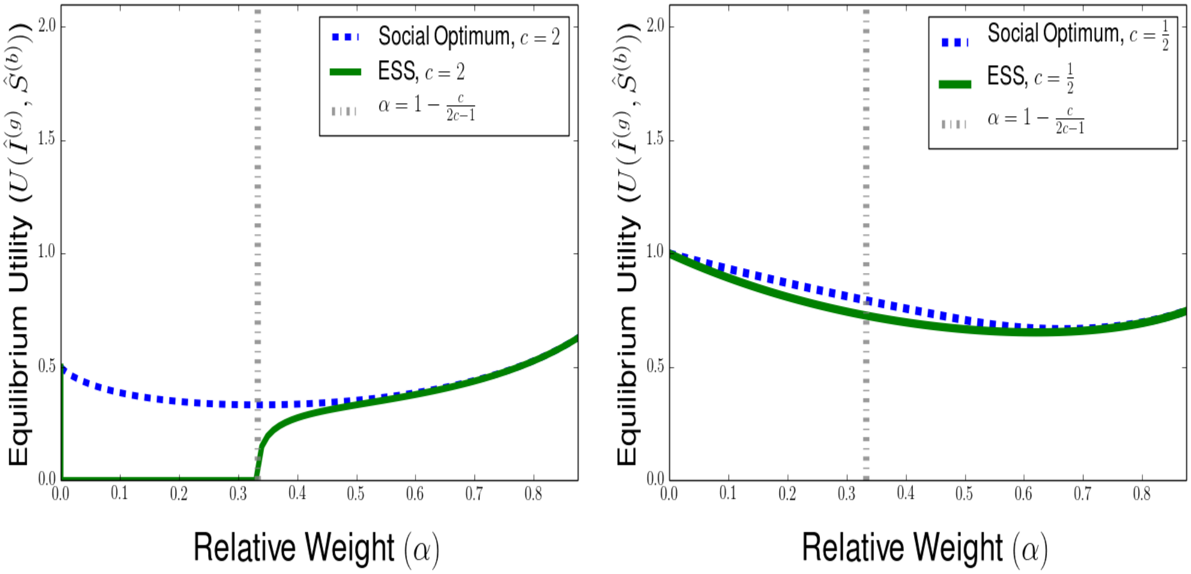

Figure 2: Utility for individuals at evolutionarily-stable and socially-optimal levels of sociality. We plot these utilities as a function of the relative importance of the good contagion α and for the relative infectiousness values $c = 2$ (left) and $c = \frac{1}{2}$ (right).

Another quantity often used in game theory to compare the efficiency of Nash equilibria and social optima is the Price of Anarchy (PoA), introduced by Papadimitriou [9, 10], which measures the ratio of the utilities between such outcomes [9–12]. In our context, we can compare the evolutionarily-stable and socially-outcomes by defining the PoA as

$$\mathrm{PoA} := \frac{U[\mathcal{R}_{\mathrm{ESS}}^{(g)}, \mathcal{R}_{\mathrm{ESS}}^{(g)}]}{U[\mathcal{R}_{\mathrm{opt}}^{(g)}, \mathcal{R}_{\mathrm{opt}}^{(g)}]}. \tag{S1.B.4}$$

Because the Cobb-Douglas utility is non-negative and $\mathcal{R}_{\mathrm{opt}}^{(g)}$ maximizes $U[\mathcal{R}_m^{(g)}, \mathcal{R}_r^{(g)}]$, the PoA takes on values between 0 and 1. In Figure 3, we plot the PoA as a function of α for the cases $c = \frac{1}{2}$ and $c = 2$. For $c = 2$, the PoA is 0 for $\alpha < \frac{1}{3}$, when the evolutionarily-stable population is achieving the worst possible payoff. The PoA turns out to be a non-decreasing function of α when $c = 2$, while the inefficiency of the evolutionarily-stable outcome is maximized at an intermediate value of α when $c = \frac{1}{2}$.

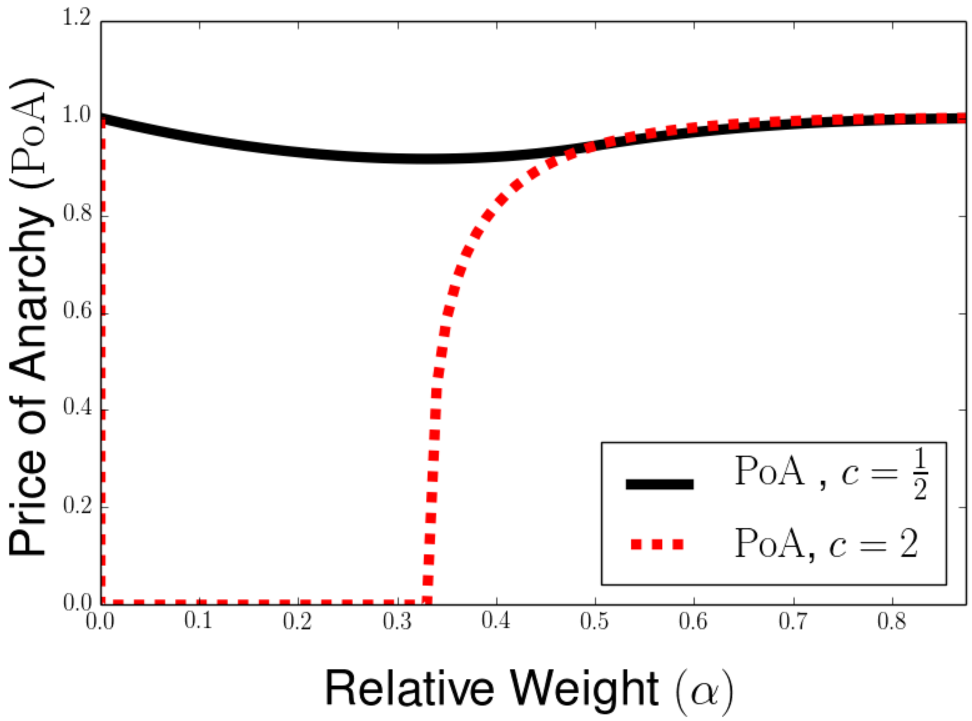

Figure 3: The Price-of-Anarchy (PoA) characterizing the efficiency of the evolutionarily-stable sociality $\mathcal{R}^{(g)}_{\text{ESS}}$ relative to the socially-optimal sociality $\mathcal{R}^{(g)}_{\text{opt}}$. The PoA is plotted as a function of α, and we consider the cases $c = \frac{1}{2}$ (black solid line) and $c = 2$ (red dashed line).

B.3 Evolutionary and Convergence Stability for Cobb-Douglas Utility

Now we will further examine the evolutionary and convergence stability of the sociality strategy $\mathcal{R}^{(g)}_{\text{ESS}} = \max\left(1, \dfrac{1}{c} + \dfrac{\alpha}{1-\alpha}\right)$ in the case of Cobb-Douglas utility using the classification criteria for evolutionarily singular strategies [13–15]. For a strategy $(\mathcal{R}^{(g)}_r)^*$ to be evolutionarily-stable, the strategy must be a local maximizer of the relative utility function $s_{(\mathcal{R}^{(g)}_r)^*}(\mathcal{R}^{(g)}_m)$ [15]. This always holds in the boundary case when $(\mathcal{R}^{(g)}_r)^* = 1$ because $s_{(\mathcal{R}^{(g)}_r)^*}(\mathcal{R}^{(g)}_m)$ is decreasing for $\mathcal{R}^{(g)}_m$ near 1. For the interior case in which $(\mathcal{R}^{(g)}_r)^* = \frac{1}{c} + \frac{\alpha}{1-\alpha}$, we will apply the second derivative test. We see

that

$$
\begin{aligned}
&\frac{\partial^2 s_{\mathcal{R}_r^{(g)}}(\mathcal{R}_m^{(g)})}{\partial\left(\mathcal{R}_m^{(g)}\right)^2}\Bigg|_{\mathcal{R}_m^{(g)}=\mathcal{R}_r^{(g)}} \\
&= \left[\frac{\partial}{\partial \mathcal{R}_m^{(g)}}\left(\frac{\partial \log\left[U\left(\mathcal{R}_m^{(g)},\mathcal{R}_r^{(g)}\right)\right]}{\partial \mathcal{R}_m^{(g)}}\right)\right]\Bigg|_{\mathcal{R}_m^{(g)}=\mathcal{R}_r^{(g)}} \\
&= \left[\frac{\partial}{\partial \mathcal{R}_m^{(g)}}\left(\frac{\alpha \mathcal{R}_r^{(g)}}{\mathcal{R}_m^{(g)}\left(\mathcal{R}_m^{(g)}\mathcal{R}_r^{(g)}+\mathcal{R}_r^{(g)}-\mathcal{R}_m^{(g)}\right)}-\frac{(1-\alpha)\left(c\mathcal{R}_r^{(g)}-1\right)}{c\mathcal{R}_m^{(g)}\mathcal{R}_r^{(g)}+\mathcal{R}_r^{(g)}-\mathcal{R}_m^{(g)}}\right)\right]\Bigg|_{\mathcal{R}_m^{(g)}=\mathcal{R}_r^{(g)}} \qquad \text{(S1.B.5)} \\
&= \left[-\frac{\alpha \mathcal{R}_r^{(g)}\left(2\mathcal{R}_m^{(g)}\mathcal{R}_r^{(g)}+\mathcal{R}_r^{(g)}-2\mathcal{R}_m^{(g)}\right)}{\left(\mathcal{R}_r^{(g)}(\mathcal{R}_m^{(g)})^2+\mathcal{R}_m^{(g)}\mathcal{R}_r^{(g)}-(\mathcal{R}_m^{(g)})^2\right)^2}+\frac{(1-\alpha)\left(c\mathcal{R}_r^{(g)}-1\right)^2}{\left(c\mathcal{R}_r^{(g)}\mathcal{R}_m^{(g)}+\mathcal{R}_r^{(g)}-\mathcal{R}_m^{(g)}\right)^2}\right]\Bigg|_{\mathcal{R}_m^{(g)}=\mathcal{R}_r^{(g)}} \\
&= \frac{1}{(\mathcal{R}_m^{(g)})^4}\left[-\alpha\left(2\mathcal{R}_m^{(g)}-1\right)+\left(\frac{1-\alpha}{c^2}\right)\left(c\mathcal{R}_m^{(g)}-1\right)^2\right]
\end{aligned}
$$

Then, evaluating the derivative at the interior singular strategy $\left(\mathcal{R}_m^{(g)}\right)^* = \frac{\alpha}{1-\alpha}+\frac{1}{c}$, we get that

$$
\frac{\partial^2 s_{\mathcal{R}_r^{(g)}}(\mathcal{R}_m^{(g)})}{\partial\left(\mathcal{R}_m^{(g)}\right)^2}\Bigg|_{\mathcal{R}_m^{(g)}=\mathcal{R}_r^{(g)}=\frac{\alpha}{1-\alpha}+\frac{1}{c}} = -\frac{\alpha^2}{1-\alpha}-\alpha\left(\frac{2}{c}-1\right) \qquad \text{(S1.B.6)}
$$

The interior singular strategy $(\mathcal{R}_r^{(g)})^*$ is an ESS when the righthand side of Equation (S1.B.5) is negative, which occurs when

$$
c < 2\left(\frac{1-\alpha}{1-2\alpha}\right) \qquad \text{(S1.B.7)}
$$

Because we know from Equation (25) that $(\mathcal{R}_r^{(g)})^*$ is infeasible for $c > \frac{1-\alpha}{1-2\alpha}$, it follows that the interior singular strategy $(\mathcal{R}_r^{(g)})^*$ is an ESS whenever it corresponds to a feasible replication number for the good contagion.

Having shown that $(\mathcal{R}_r^{(g)})^*$ is evolutionarily-stable under local mutation, we can now address the convergence stability of the equilibrium (i.e. whether such a stable equilibrium could actually be achieved starting under evolution from a nearby sociality strategy). For an endpoint singular strategy, convergence stability follows from the sign of the local selection gradient near the boundary. To demonstrate convergence stability for interior singular strategies, we require that that the local selection gradient is an increasing function at the evolutionarily singular strategy [13–15]. For the singular strategy $(\mathcal{R}_r^{(g)})^* = \dfrac{1}{c}+\frac{\alpha}{1-\alpha}$ to be convergence stable, we need to verify that

$$
s''_{\mathcal{R}_r^{(g)}}(\mathcal{R}_r^{(g)}) = \frac{\partial^2 \log\left[U(\mathcal{R}_m^{(g)},\mathcal{R}_r^{(g)})\right]}{\partial\left(\mathcal{R}_m^{(g)}\right)^2}\Bigg|_{\mathcal{R}_m^{(g)}=\mathcal{R}_r^{(g)}=(\mathcal{R}_r^{(g)})^*} + \frac{\partial^2 \log\left[U(\mathcal{R}_m^{(g)},\mathcal{R}_r^{(g)})\right]}{\partial \mathcal{R}_m^{(g)}\partial \mathcal{R}_r^{(g)}}\Bigg|_{\mathcal{R}_m^{(g)}=\mathcal{R}_r^{(g)}=(\mathcal{R}_r^{(g)})^*} < 0 \qquad \text{(S1.B.8)}
$$

For the second term, we calculate that

$$
\begin{aligned}
&\frac{\partial^2 \log\left[U(\mathcal{R}_m^{(g)}, \mathcal{R}_r^{(g)})\right]}{\partial \mathcal{R}_m^{(g)} \partial \mathcal{R}_r^{(g)}}\Bigg|_{\mathcal{R}_m^{(g)}=\mathcal{R}_r^{(g)}} \\
&\quad = \frac{\partial}{\partial \mathcal{R}_r^{(g)}} \left(\frac{\alpha}{\left(\mathcal{R}_r^{(g)} - 1\right)\left(\mathcal{R}_m^{(g)}\mathcal{R}_r^{(g)} + \mathcal{R}_r^{(g)} - \mathcal{R}_m^{(g)}\right)} - \frac{(1-\alpha)\,\mathcal{R}_m^{(g)}}{\mathcal{R}_r^{(g)}\left(c\mathcal{R}_m^{(g)}\mathcal{R}_r^{(g)} + \mathcal{R}_r^{(g)} - \mathcal{R}_m^{(g)}\right)} \right)\Bigg|_{\mathcal{R}_m^{(g)}=\mathcal{R}_r^{(g)}} \\
&\quad = \left[\frac{-\alpha}{\left(\mathcal{R}_m^{(g)}\mathcal{R}_r^{(g)} + \mathcal{R}_r^{(g)} - \mathcal{R}_m^{(g)}\right)^2} - \frac{1-\alpha}{\left(c\mathcal{R}_m^{(g)}\mathcal{R}_r^{(g)} + \mathcal{R}_r^{(g)} - \mathcal{R}_m^{(g)}\right)^2} \right]\Bigg|_{\mathcal{R}_m^{(g)}=\mathcal{R}_r^{(g)}} \\
&\quad = -\frac{1}{\left(\mathcal{R}_r^{(g)}\right)^4} \left[\alpha + \left(\frac{1-\alpha}{c^2}\right)\right]
\end{aligned}
\tag{S1.B.9}
$$

Combining Equations (S1.B.5) and (S1.B.9), we can see that

$$
\begin{aligned}
s''_{\mathcal{R}_r^{(g)}}(\mathcal{R}_r^{(g)}) &= \frac{1}{\left(\mathcal{R}_r^{(g)}\right)^4}\left[-\alpha\left(2\mathcal{R}_r^{(g)} - 1\right) + \left(\frac{1-\alpha}{c}\right)\left(c\mathcal{R}_r^{(g)} - 1\right)^2 - \left(\alpha + \frac{1-\alpha}{c^2}\right)\right]\Bigg|_{\mathcal{R}_r^{(g)}=(\mathcal{R}_r^{(g)})^*} \\
&= \frac{1}{\left(\mathcal{R}_r^{(g)}\right)^3}\left[c\,(1-\alpha)\,(\mathcal{R}_r^{(g)})^* - 2\,(1-\alpha+\alpha c))\right]
\end{aligned}
\tag{S1.B.10}
$$

Therefore we see that $s''_{\mathcal{R}_r^{(g)}}(\mathcal{R}_r^{(g)}) < 0$ when

$$
(\mathcal{R}_r^{(g)})^* < \frac{2(1-\alpha) + 2\alpha c}{c(1-\alpha)} = 2\left(\frac{\alpha}{1-\alpha} + \frac{1}{c}\right)
\tag{S1.B.11}
$$

Our ESS $(\mathcal{R}_r^{(g)})^* = \frac{\alpha}{1-\alpha} + \frac{1}{c}$ satisfies this condition, so we can conclude that $(\mathcal{R}_r^{(g)})^*$ is convergence stable whenever it is biologically feasible.

B.4 Linear Utility Function

We can also consider a utility function which places a convex combination of weight on the fractions of time spent informed for the good contagion and susceptible to the bad contagion. This utility function takes the form

$$
U[\mathcal{R}_m^{(g)}, \mathcal{R}_r^{(g)}] := \alpha \hat{I}_m^{(g)}(\mathcal{R}_m^{(g)}, \mathcal{R}_r^{(g)}) + (1-\alpha)\left(\hat{S}_m^{(b)}(\mathcal{R}_m^{(g)}, \mathcal{R}_r^{(g)})\right).
\tag{S1.B.12}
$$

For a monomorphic population, we can use the expressions from Equation (5) for the endemic equilibria to see that the linear utility function takes the following form:

$$
U[\mathcal{R}_r^{(g)}, \mathcal{R}_r^{(g)}] = \begin{cases}
1-\alpha & : \mathcal{R}_r^{(g)} > 1, \dfrac{1}{c} \\
\alpha\left(1 - \dfrac{1}{\mathcal{R}_r^{(g)}}\right) + (1-\alpha) & : 1 < \mathcal{R}_r^{(g)} \leq \dfrac{1}{c} \\
(1-\alpha)\left(\dfrac{1}{c\mathcal{R}_r^{(g)}}\right) & : \frac{1}{c} < \mathcal{R}_r^{(g)} \leq 1 \\
\alpha\left(1 - \dfrac{1}{\mathcal{R}_r^{(g)}}\right) + (1-\alpha)\left(\dfrac{1}{c\mathcal{R}_r^{(g)}}\right) & : \mathcal{R}_r^{(g)} > 1, \dfrac{1}{c}
\end{cases}.
\tag{S1.B.13}
$$

Because $U[\mathcal{R}_r^{(g)}, \mathcal{R}_r^{(g)}]$ is a piecewise affine function of $\frac{1}{\mathcal{R}_r^{(g)}}$, we see that social optimum will be achieved at one of the endpoints $\mathcal{R}_r^{(g)} \in \{1, \frac{1}{c}, \infty\}$. This utility function is always increasing when $1 < \mathcal{R}_r^{(g)} < \dfrac{1}{c}$, always decreasing when $\frac{1}{c} < \mathcal{R}_r^{(g)} < 1$, and is increasing for $\mathcal{R}_r^{(g)} > \dfrac{1}{c}$ when $\alpha c > 1 - \alpha$. When the latter condition holds, we see that the socially-optimal sociality strategy is $\mathcal{R}_{\mathrm{opt}}^{(g)} = \infty$. If instead, $\alpha c < 1 - \alpha$, then $U[\mathcal{R}_r^{(g)}, \mathcal{R}_r^{(g)}]$ is decreasing for $\mathcal{R}_r^{(g)} > \dfrac{1}{c}$, $\mathcal{R}_{\mathrm{opt}}^{(g)} = \frac{1}{c}$. For the case in which $\mathcal{R}_{\mathrm{opt}}^{(g)} = \frac{1}{c}$, this social optimum will feature long-time survival of the good contagion and elimination of the bad contagion when $c < 1$, and will feature long-time extinction of both contagions when $c \geq 1$.

Turning to the question of evolutionary stability, we consider the following expression for the relative advantage of a mutant over a resident

$$s_{\mathcal{R}_r^{(g)}}(\mathcal{R}_m^{(g)}) := U\left[\mathcal{R}_m^{(g)}, \mathcal{R}_r^{(g)}\right] - U\left[\mathcal{R}_r^{(g)}, \mathcal{R}_r^{(g)}\right]. \tag{S1.B.14}$$

This allows us to compute the local selection gradient as

$$s'_{\mathcal{R}_r^{(g)}}(\mathcal{R}_r^{(g)}) = \alpha \left.\frac{\partial \hat{I}_m^{(g)}(\mathcal{R}_m^{(g)}, \mathcal{R}_r^{(g)})}{\partial \mathcal{R}_m^{(g)}}\right|_{\mathcal{R}_m^{(g)} = \mathcal{R}_r^{(g)}} + (1-\alpha) \left.\frac{\partial \hat{S}_m^{(b)}(\mathcal{R}_m^{(g)}, \mathcal{R}_r^{(g)})}{\partial \mathcal{R}_m^{(g)}}\right|_{\mathcal{R}_m^{(g)} = \mathcal{R}_r^{(g)}}. \tag{S1.B.15}$$

Using Equation (18), we can further write the selection gradient as

$$s'_{\mathcal{R}_r^{(g)}}(\mathcal{R}_r^{(g)}) = \begin{cases} 0 & : \mathcal{R}_r^{(g)} \leq 1, \dfrac{1}{c} \\ \dfrac{1}{(\mathcal{R}_r^{(g)})^3}\left(\mathcal{R}_r^{(g)} - 1\right) & : 1 < \mathcal{R}_r^{(g)} \leq \dfrac{1}{c} \\ -\dfrac{1}{c^2(\mathcal{R}_r^{(g)})^3}\left(c\mathcal{R}_r^{(g)} - 1\right) & : \dfrac{1}{c} < \mathcal{R}_r^{(g)} \leq 1 \\ \dfrac{1}{c^2(\mathcal{R}_r^{(g)})^3}\left[(\alpha c + \alpha - 1)\, c\mathcal{R}_r^{(g)} + 1 - \alpha - \alpha c^2\right] & : \mathcal{R}_r^{(g)} > 1, \dfrac{1}{c} \end{cases}. \tag{S1.B.16}$$

Notably, this means that the selection gradient is always increasing if $\mathcal{R}_r^{(g)} \in (\frac{1}{c}, 1)$ and always descreasing if $\mathcal{R}_r^{(g)} \in (1, \frac{1}{c})$, but that the sign of the selection gradient for $\mathcal{R}_r^{(g)} > \max\{1, \frac{1}{c}\}$ will depend on the sign of the slope for the term in square brackets that is linear in $\mathcal{R}_r^{(g)}$. For the case in which both contagions spread (when $\mathcal{R}_r^{(g)} > \max\{1, \frac{1}{c}\}$ we see that there is a possible interior evolutionarily singular strategy given by

$$\mathcal{R}_{\mathrm{int}}^{(g)} := 1 + \frac{(1-\alpha)(c-1)}{1 - \alpha - \alpha c}, \tag{S1.B.17}$$

which satisfies the feasibility condition $\mathcal{R}_{\mathrm{int}}^{(g)} \geq 1$ when the signs of $c - 1$ and $1 - \alpha - \alpha c$ agree. As a result, the the evolutionary stability of the strategies $\mathcal{R}_r^{(g)} = \infty$, and $\mathcal{R}_{\mathrm{int}}^{(g)}$ both depend on the signs of $c - 1$ and $\alpha c - (1 - \alpha)$.

In Table 1, we compare the evolutionarily-stable and socially-optimal sociality strategies $\mathcal{R}_{\mathrm{ESS}}^{(g)}$ and $\mathcal{R}_{\mathrm{opt}}^{(g)}$ for the possible ranges of values of the relative spreading ability c of the two contagions and the weight α placed on infection with the good contagion under the linear utility function. For all of the cases in the table, we see that either the evolutionarily-stable outcome coincides with the social

optimum, or there is a social dilemma featuring a discrepancy between the levels of sociality whose direction coincides with the social dilemma in the Cobb-Douglas case (e.g. $\mathcal{R}^{(g)}_{\text{ESS}} \leq \mathcal{R}^{(g)}_{\text{opt}}$ for $c > 1$ and $\mathcal{R}^{(g)}_{\text{ESS}} \leq \mathcal{R}^{(g)}_{\text{opt}}$ when $c < 1$). Notably, when $c > 1$ and $c > \frac{1-\alpha}{\alpha}$, we find that the evolutionary dynamics support bistability of $\frac{1}{c}$ and ∞ as evolutionarily-stable states, whose basins of attraction are separated by an evolutionarily unstable state $\mathcal{R}^{(g)}_{\text{int}}$. In Figure 4, we further illustrate the various parameter regimes in which these socially-optimal and evolutionarily-stable are achieved.

	$c > \dfrac{1-\alpha}{\alpha}$	$c < \dfrac{1-\alpha}{\alpha}$
$c > 1$	$\mathcal{R}^{(g)}_{\text{ESS}} \in \left\{\frac{1}{c}, \infty\right\}$<br>$\mathcal{R}^{(g)}_{\text{opt}} = \infty$	$\mathcal{R}^{(g)}_{\text{ESS}} = \frac{1}{c}$<br>$\mathcal{R}^{(g)}_{\text{opt}} = \frac{1}{c}$
$c < 1$	$\mathcal{R}^{(g)}_{\text{ESS}} = \infty$<br>$\mathcal{R}^{(g)}_{\text{opt}} = \infty$	$\mathcal{R}^{(g)}_{\text{ESS}} = 1 + \dfrac{(1-\alpha)(1-c)}{c(1-\alpha-\alpha c)} > \frac{1}{c}$<br>$\mathcal{R}^{(g)}_{\text{opt}} = \frac{1}{c}$

Table 1: Evolutionarily-stable and socially-optimal sociality strategies $\mathcal{R}^{(g)}_{\text{ESS}}$ and $\mathcal{R}^{(g)}_{\text{opt}}$ for different cases on the relative values of the relative weight α of the good contagion under linear utility and the relative infectiousness c of the good contagion.

B.5 Constant Elasticity of Substitution (CES) Utility

In this section, we consider the evolutionary dynamics for the CES utility function, introduced by Arrow [16, 17], which takes the following form

$$U\left[\mathcal{R}^{(g)}_m, \mathcal{R}^{(g)}_r\right] := \left[\alpha\left(I^{(g)}_m(\mathcal{R}^{(g)}_m, \mathcal{R}^{(g)}_r)\right)^{\rho} + (1-\alpha)\left(S^{(b)}_m(\mathcal{R}^{(g)}_m, \mathcal{R}^{(g)}_r)\right)^{\rho}\right]^{\dfrac{1}{\rho}}, \tag{S1.B.18}$$

where we consider values of the substitution parameter $\rho \in (0,1)$. We note that the CES utility function takes the form of the linear utility function from Equation (S1.B.12) when we set $\rho = 1$, and that we recover the Cobb-Douglas utility function in the form of Equation (6) in the limit as $\rho \to 0$. The parameter α governs the relative weight placed on acquiring the good contagion versus avoiding the bad contagion, and we see that this parameter retains this interpretation when we obtain the linear and Cobb-Douglas utility functions in the limits as $\rho \to 1$ and $\rho \to 0$, respectively.

For a monomorphic population following the sociality strategy $\mathcal{R}^{(g)}_r$, we can use the expressions from Equation (5) for the endemic equilibria for the good and bad contagion to write the following piecewise characterization of the CES utility:

$$U\left[\mathcal{R}^{(g)}_r, \mathcal{R}^{(g)}_r\right] = \begin{cases} (1-\alpha)^{\frac{1}{\rho}} & : \mathcal{R}^{(g)}_r < 1, \frac{1}{c} \\ (1-\alpha)^{\frac{1}{\rho}}\left[\dfrac{1}{c\mathcal{R}^{(g)}_r}\right] & : \frac{1}{c} < \mathcal{R}^{(g)}_r \leq 1 \\ \alpha^{\frac{1}{\rho}}\left[1 - \dfrac{1}{\mathcal{R}^{(g)}_r}\right] & : 1 < \mathcal{R}^{(g)}_r \leq \frac{1}{c} \\ \left[\alpha\left(1 - \dfrac{1}{\mathcal{R}^{(g)}_r}\right)^{\rho} + (1-\alpha)\left[\dfrac{1}{c\mathcal{R}^{(g)}_r}\right]^{\rho}\right]^{\frac{1}{\rho}} & : \mathcal{R}^{(g)}_r > 1, \frac{1}{c} \end{cases}. \tag{S1.B.19}$$

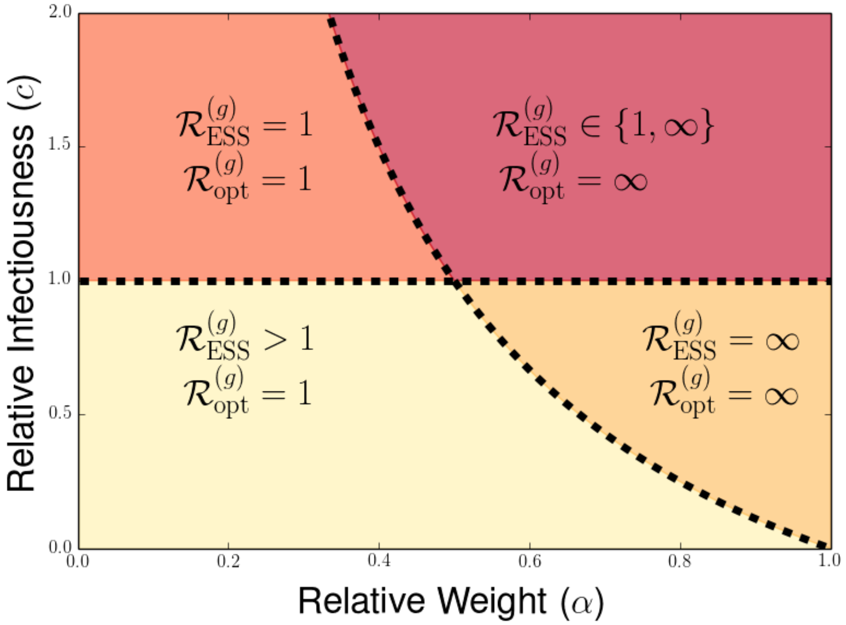

Figure 4: Illustration of the four possible qualitative behaviors for $\mathcal{R}^{(g)}_{\text{opt}}$ and $\mathcal{R}^{(g)}_{\text{ESS}}$ across the range of relative levels of infectiousness for the bad contagion c and relative weight placed on the good contagion α under linear utility. First, parameter space is divided into two regions, one in which $\alpha c < 1-\alpha$ and $\mathcal{R}^{(g)}_{\text{opt}} = 1$ (light yellow and orange), and the other in which $\alpha c > 1-\alpha$ and $\mathcal{R}^{(g)}_{\text{opt}} = \infty$ (dark yellow and red). We further subdivide the region in which $\alpha c < 1-\alpha$ into the cases in which $c < 1$ and $\mathcal{R}^{(g)}_{\text{ESS}} > 1$ (light yellow) and in which $c > 1$ and $\mathcal{R}^{(g)}_{\text{ESS}} \geq 1$ (orange). The region in which $\alpha c > 1-\alpha$ is further subdivided into regions in which $c < 1$ and $\mathcal{R}^{(g)}_{\text{ESS}} = \infty$ (dark yellow) and a region in which $c > 1$ and the evolutionary dynamics feature bistability between $\mathcal{R}^{(g)}_{\text{ESS}} = 1$ and $\mathcal{R}^{(g)}_{\text{ESS}} = \infty$ (red).

Because the CES utility function is twice-differentiable and strictly concave for $\rho \in (0,1)$, we know from our analysis in the section that the utility function $U[\mathcal{R}^{(g)}_r, \mathcal{R}^{(g)}_r]$ has a unique local maximizer for $\mathcal{R}^{(g)}_r \geq \frac{1}{c}$. For $c < 1$, this local maximizer is actually the global maximizer for biologically feasible $\mathcal{R}^{(g)}_r$ satisfying $\mathcal{R}^{(g)}_r \geq 0$.

However, we notice also notice that $U\left[\mathcal{R}^{(g)}_r, \mathcal{R}^{(g)}_r\right]$ is decreasing on $(\frac{1}{c}, 1)$ for the case in which $c > 1$. In this case, $\mathcal{R}^{(g)}_r = \frac{1}{c}$ is also a local maximizer of $U\left[\mathcal{R}^{(g)}_r, \mathcal{R}^{(g)}_r\right]$, which has utility $U[\frac{1}{c}, \frac{1}{c}] = (1-\alpha)^{\frac{1}{\rho}}$. By considering the limit $\lim_{\mathcal{R}^{(g)}_r \to \infty} U\left[\mathcal{R}^{(g)}_r, \mathcal{R}^{(g)}_r\right] = \alpha^{\frac{1}{\rho}}$, we can deduce that $\alpha > \frac{1}{2}$ (and correspondingly $\alpha > 1-\alpha$) is a sufficient condition for the maximizer $U\left[\mathcal{R}^{(g)}_r, \mathcal{R}^{(g)}_r\right]$ be achieved by a sociality strategy $\mathcal{R}^{(g)}_{\text{opt}} > 1$ in which the good contagion is present at equilibrium. In particular, this means that, whenever more emphasis is placed on the good contagion than the bad contagion, the socially-optimal sociality strategy will feature spread of both the good and bad contagion when $c < 1$.

Next, we turn to the adaptive dynamics analysis for studying the evolution of sociality strategies under CES utility. When $c = 1$, we can use Equation (35) to see that the derivative of the

monomorphic utility and selection gradient coincide, and therefore $\mathcal{R}_{\text{ESS}}^{(g)} = \mathcal{R}_{\text{opt}}^{(g)}$ in this case. When $c < 1$, we know from the discussion in the General Utility Function section that there will be a unique socially-optimal strategy $\mathcal{R}_{\text{opt}}^{(g)} \geq \frac{1}{c} > 1$, and that any evolutionarily stable strategy $\mathcal{R}_{\text{ESS}}^{(g)}$ will satisfy $\mathcal{R}_{\text{ESS}}^{(g)} > \mathcal{R}_{\text{opt}}^{(g)}$. In Figure 5, we illustrate the socially-optimal and evolutionarily-stable sociality strategies $\mathcal{R}_{\text{opt}}^{(g)}$ and $\mathcal{R}_{\text{ESS}}^{(g)}$ for the CES utility function when $c = \frac{1}{2}$. We see that $\mathcal{R}_{\text{ESS}}^{(g)} > \mathcal{R}_{\text{opt}}^{(g)}$ when any weight is placed on acquiring the good contagion (i.e. $\alpha > 0$), and the form of the social dilemma resembles the qualitative behavior seen for Cobb-Douglas utility in Figure 4(c,bottom) in the case of $c < 1$.

Finally, we turn to analyze the case of $c > 1$, in which there are two local maxima for the social utility $U[\mathcal{R}_r^{(g)}, \mathcal{R}_r^{(g)}]$. Using Equation (32b) and the fact that and $\dfrac{\partial U[\mathcal{R}_r^{(g)}, \mathcal{R}_r^{(g)}]}{\partial \hat{S}^{((b))}(\mathcal{R}_r^{(g)}, \mathcal{R}_r^{(g)})} > 0$, $\dfrac{\partial \hat{S}^{((b))}(\mathcal{R}_r^{(g)}, \mathcal{R}_r^{(g)})}{\partial \mathcal{R}_r^{(g)}} < 0$, and $\dfrac{\partial \hat{I}^{((g))}(\mathcal{R}_r^{(g)}, \mathcal{R}_r^{(g)})}{\partial \mathcal{R}_r^{(g)}} = 0$ for $c > 1$ and $\mathcal{R}_r^{(g)} < 1$, we see that, in this regime,

$$s'_{\mathcal{R}_r^{(g)}}(\mathcal{R}_r^{(g)}) = \frac{\partial U[\mathcal{R}_r^{(g)}, \mathcal{R}_r^{(g)}]}{\partial \hat{I}^{((g))}(\mathcal{R}_r^{(g)}, \mathcal{R}_r^{(g)})} \frac{\partial \hat{I}^{((g))}(\mathcal{R}_r^{(g)}, \mathcal{R}_r^{(g)})}{\partial \mathcal{R}_r^{(g)}} + \frac{\partial U[\mathcal{R}_r^{(g)}, \mathcal{R}_r^{(g)}]}{\partial \hat{S}^{((b))}(\mathcal{R}_r^{(g)}, \mathcal{R}_r^{(g)})} \frac{\partial \hat{S}^{((b))}(\mathcal{R}_r^{(g)}, \mathcal{R}_r^{(g)})}{\partial \mathcal{R}_r^{(g)}} < 0.$$

Because the local selection gradient $s'_{\mathcal{R}_r^{(g)}}(\mathcal{R}_r^{(g)})$ is negative for when $\frac{1}{c} < \mathcal{R}_r^{(g)} < 1$, we can deduce that the sociality strategy $\mathcal{R}_r^{(g)} = \frac{1}{c}$ will be evolutionarily stable.

To further understand the basin of attraction of this state under adaptive dynamics, we now look to calculate the selection gradient for $\mathcal{R}_r^{(g)} > 1$. Our goal is to use Equation (35) to calculate the local selection gradient for the CES utility when $c > 1$ and $\mathcal{R}_r^{(g)} > 1$, and we are particularly interested in the behavior of the adaptive dynamics in the case as $\mathcal{R}_r^{(g)} \to 1^+$. We recall from Equation (35) that the local selection gradient for $\mathcal{R}_r^{(g)} > \max\{1, \frac{1}{c}\}$ takes the following form

$$\begin{aligned} s'_{\mathcal{R}_r^{(g)}}(\mathcal{R}_r^{(g)}) = & \left(\frac{\mathcal{R}_r^{(g)} - 1}{\mathcal{R}_r^{(g)}} \right) \frac{\partial U[\mathcal{R}_r^{(g)}, \mathcal{R}_r^{(g)}]}{\partial \mathcal{R}_r^{(g)}} \\ & + \frac{1}{c \left(\mathcal{R}_r^{(g)} \right)^3} \left(\frac{1}{c} - 1 \right) \frac{\partial U[\mathcal{R}_r^{(g)}, \mathcal{R}_r^{(g)}]}{\partial \hat{S}^{(b)}(\mathcal{R}_r^{(g)}, \mathcal{R}_r^{(g)})}. \end{aligned}$$

We will now compute the main quantities that appear in this expression for the local selection gradient. We first differentiate the expression for CES utility from Equation (S1.B.19) in the case of $\mathcal{R}_r^{(g)} > \max\{1, \frac{1}{c}\}$, which allows us to see that

$$\begin{aligned} \frac{\partial U\left[\mathcal{R}_r^{(g)}, \mathcal{R}_r^{(g)}\right]}{\partial \mathcal{R}_r^{(g)}} = & \left[\alpha \left(1 - \frac{1}{\mathcal{R}_r^{(g)}} \right)^{\rho} + (1 - \alpha) \left(\frac{1}{c \mathcal{R}_r^{(g)}} \right)^{\rho} \right]^{\frac{1}{\rho} - 1} \\ & \times \left(\frac{1}{\mathcal{R}_r^{(g)}} \right)^2 \left[\alpha \left(\frac{\mathcal{R}_r^{(g)} - 1}{\mathcal{R}_r^{(g)}} \right)^{\rho - 1} - \left(\frac{1 - \alpha}{c} \right) \left(\frac{1}{c \mathcal{R}_r^{(g)}} \right)^{\rho - 1} \right]. \end{aligned} \tag{S1.B.20}$$

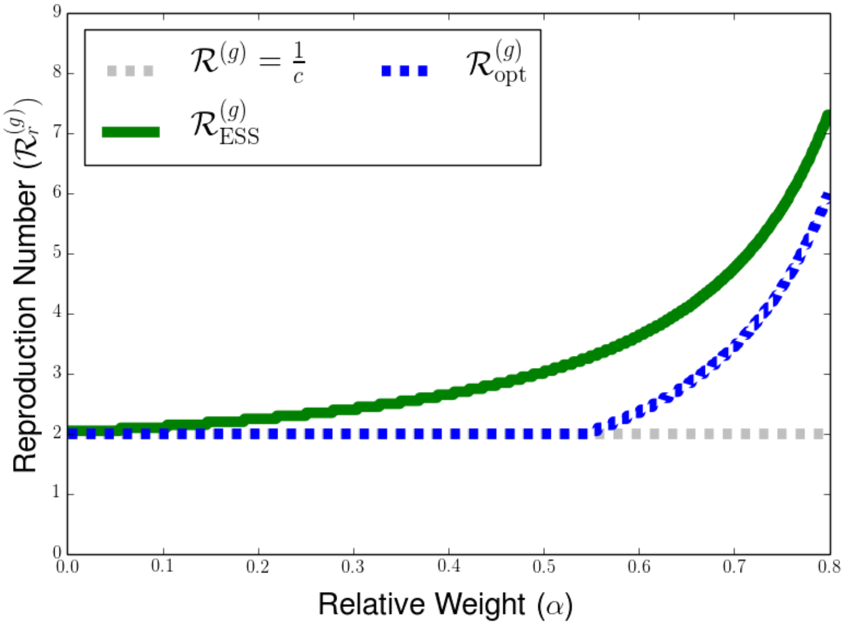

Figure 5: Illustration of social dilemma for adaptive dynamics under CES utility function when $c = \frac{1}{2}$. The reproduction numbers $\mathcal{R}_r^{(g)}$ are presented for both evolutionarily-stable sociality strategy $\mathcal{R}^{(g)}_{\text{ESS}}$ (solid green line) and the socially-optimal sociality strategy $\mathcal{R}^{(g)}_{\text{opt}}$ (dashed blue line), and are plotted as functions of the weight parameter α describing the relative importance placed upon the good contagion. The dashed gray line represents $\mathcal{R}_r^{(g)} = \frac{1}{c}$, the reproduction number corresponding to the highest level of social interaction at which the bad contagion does not spread. The substitution parameter is fixed at $\rho = 0.5$, and the weight parameter α ranges between 0 and 0.8.

In the limit as $\mathcal{R}_r^{(g)} \to 1^+$, we can use our expression from Equation (S1.B.20) and the fact that we are considering CES utility parameters with substitution parameters $\rho > 0$ to deduce that

$$\begin{aligned}
&\lim_{\mathcal{R}_r^{(g)} \to 1^+} \left[\left(\frac{\mathcal{R}_r^{(g)} - 1}{\mathcal{R}_r^{(g)}} \right) \frac{\partial U\left[\mathcal{R}_r^{(g)}, \mathcal{R}_r^{(g)}\right]}{\partial \mathcal{R}_r^{(g)}} \right] \\
&= \left(\frac{1-\alpha}{c} \right)^{\frac{1}{\rho}-1} \lim_{\mathcal{R}_r^{(g)} \to 1^+} \left[\alpha \left(\frac{\mathcal{R}_r^{(g)} - 1}{\mathcal{R}_r} \right)^{\rho} - \left(\frac{1-\alpha}{c} \right) \left(\frac{1}{c\mathcal{R}_r^{(g)}} \right)^{\rho-1} \left(\frac{\mathcal{R}_r^{(g)} - 1}{\mathcal{R}_r} \right) \right] = 0
\end{aligned} \tag{S1.B.21}$$

Next, we consider the partial derivative of utility with respect to the equilibrium level of the bad contagion. We use Equations (5) and (S1.B.18) to see that

$$\begin{aligned}
\frac{\partial U\left[\mathcal{R}_r^{(g)}, \mathcal{R}_r^{(g)}\right]}{\partial \hat{S}^{(b)}(\mathcal{R}_r^{(g)}, \mathcal{R}_r^{(g)})} &= \left[\alpha \left(\hat{I}^{((g))}(\mathcal{R}_r^{(g)}, \mathcal{R}_r^{(g)}) \right)^{\rho} + (1-\alpha) \left(\hat{S}^{((b))}(\mathcal{R}_r^{(g)}, \mathcal{R}_r^{(g)}) \right)^{\rho} \right]^{\frac{1}{\rho}-1} \\
&\quad \times (1-\alpha) \left(\hat{S}^{((b))}(\mathcal{R}_r^{(g)}, \mathcal{R}_r^{(g)}) \right)^{\rho-1} \\
&= (1-\alpha) \left[\alpha \left(1 - \frac{1}{\mathcal{R}_r^{(g)}} \right)^{\rho} + (1-\alpha) \left(\frac{1}{c\mathcal{R}_r^{(g)}} \right)^{\rho} \right]^{\frac{1}{\rho}-1} \left(\frac{1}{c\mathcal{R}_r^{(g)}} \right)^{\rho-1},
\end{aligned} \tag{S1.B.22}$$

and we see that, in the limit as $\mathcal{R}_r^{(g)} \to 1^+$

$$\left.\frac{\partial U\left[\mathcal{R}_r^{(g)}, \mathcal{R}_r^{(g)}\right]}{\partial \hat{S}^{(b)}(\mathcal{R}_r^{(g)}, \mathcal{R}_r^{(g)})}\right|_{\mathcal{R}_r^{(g)} \to 1^+} = (1-\alpha)^{\frac{1}{\rho}} > 0. \tag{S1.B.23}$$

We can now use Equations (35), (S1.B.21), and (S1.B.23) to see that, for $c > 1$, the local selection gradient satisfies

$$\left. s'_{\mathcal{R}_r^{(g)}}(\mathcal{R}_r^{(g)}) \right|_{\mathcal{R}_r^{(g)} \to 1^+} = \frac{1}{c}\left(\frac{1}{c} - 1\right)(1-\alpha)^{\frac{1}{\rho}} < 0. \tag{S1.B.24}$$

From the continuity of all of the quantities appearing in the local selection gradient of Equation (35), we can now deduce that, for $c < 1$, there will always some sociality strategies $\mathcal{R}_r^{(g)} > 1$ that will be in the basin of attraction of the boundary state $\frac{1}{c} < 1$ under the adaptive dynamics. This means that either $\frac{1}{c}$ may be a global attractor under the evolutionary dynamics, or that it will coexist in multistability with an evolutionary attractor featuring positive levels of both the good and bad contagion at equilibrium. Now, we will use a numerical approach explore these two possibilities of evolutionary collapse of social interaction and bistability of two evolutionarily-stable sociality strategies.

In Figure 6, we illustrate the CES utility function and an example of the selection gradient for the adaptive dynamics under CES utility. In Figure 6(left), we display the utility function for three different values of the weight parameter, presenting one example in which utility is maximized by the sociality strategy $\mathcal{R}_r^{(g)} = \frac{1}{c}$ (with $\alpha = 0.1$) in which neither contagion spreads, as well as two examples in which the socially-optimal sociality strategy is achieved by a strategy $\mathcal{R}_{\text{opt}}^{(g)} > 1$ under which both contagions survive at equilibrium. In Figure 6(right), we illustrate an example selection gradient for the CES utility function. We find that the selection gradient vanishes at three points. At two of these points (labeled $\mathcal{R}_{\text{ESS1}} = \frac{1}{c}$ and $\mathcal{R}_{\text{ESS2}}$), the selection gradient decreases past zero as $\mathcal{R}_r^{(g)}$ increases beyond these points, so these sociality strategies are evolutionarily stable. The third zero (labeled $\mathcal{R}_{\text{uns}} > 1$) is located at a point at which the local gradient is increasing, and therefore it is an evolutionarily-unstable singular strategy, separating the basins of attraction for the two ESS sociality strategies. Because $\mathcal{R}_{\text{uns}} > 1$, this example shows the possibility that an initial population with a sociality strategy $\mathcal{R}_r^{(g)} > 1$ can be located in the evolutionary basin of attraction of the strategy $\mathcal{R}_{\text{ESS1}}$ featuring elimination of both contagions, even though a more highly-social population can sustain both contagions under the long-time limit of the adaptive dynamics.

In Figure 7, we further illustrate the social dilemma by plotting the social optima and evolutionary singular strategies (Figure 7, left) and the CES utilities achieved for these sociality strategies (Figure 7, right) across a range of weight parameters α. We find that the sociality strategy $\mathcal{R}_{\text{ESS1}} = \frac{1}{c}$ featuring no equilibrium contagion is the unique evolutionarily-stable strategy for sufficiently small values of α, while an additional pair of singular strategies $\mathcal{R}_{\text{ESS2}}$ and $\mathcal{R}_{\text{uns}}$ appear when $\alpha \approx 0.46$, yielding bistability between $\mathcal{R}_{\text{ESS1}}$ and $\mathcal{R}_{\text{ESS2}}$. By comparing the utilities achieved these sociality strategies, we find that utility achieved by the non-trivial ESS strategy $\mathcal{R}_{\text{ESS2}}$ is less than that achieved by the social optimum $\mathcal{R}_{\text{opt}}^{(g)}$, but that the utility achieved by the equilibrium $\mathcal{R}_{\text{ESS1}}$ featuring no long-term contagion results in even lower utility than the suboptimal ESS outcome achieve by $\mathcal{R}_{\text{ESS2}}$. On top of the social dilemma realized by convergence to the evolutionarily-stable outcome of $\mathcal{R}_{\text{ESS2}}$ achieved by the adaptive dynamics for sufficiently large initial sociality stategy $\mathcal{R}_r^{(g)}$, we see that the bistability of $\mathcal{R}_{\text{ESS2}}$ and the non-interacting equilibrium $\mathcal{R}_{\text{ESS1}} = \frac{1}{c}$ that the CES utility function can achieve an even worse social dilemma and a long-time collapse of social interaction in the population.

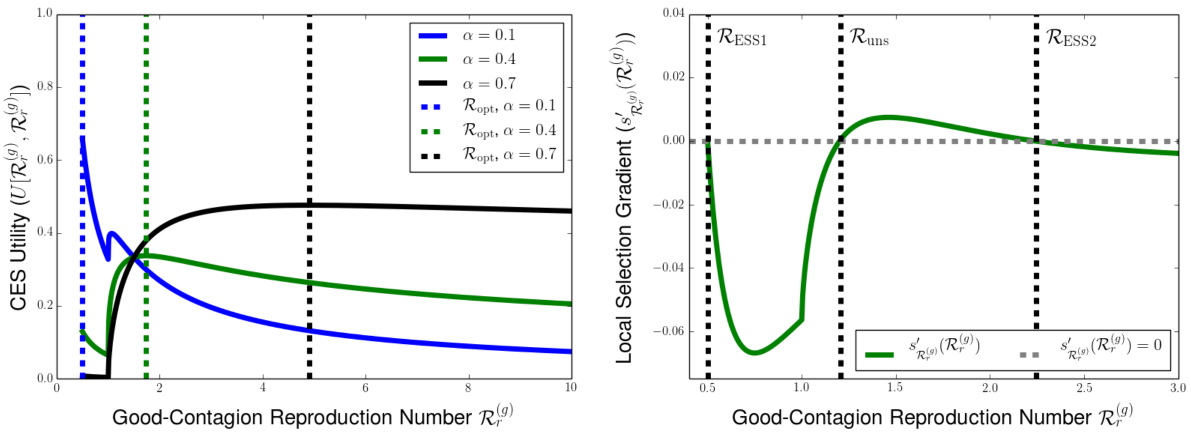

Figure 6: Illustration of the social utility (left) and selection gradient (right) for the CES utility function when the bad contagion spreads more readily than the good contagion ($c = 2$). Left: Social utility plotted as a function of the good-contagion reproduction number $\mathcal{R}_r^{(g)}$ for weight parameters $\alpha = 0.1$ (solid blue line), $\alpha = 0.4$ (solid green line), and $\alpha = 0.7$ (solid black line). Dashed lines describe the socially-optimal values of $\mathcal{R}_r^{(g)}$, with colors corresponding to the color of the utility function with the same α parameter. Right: Plot of the local selection gradient $s'_{\mathcal{R}_r^{(g)}}(\mathcal{R}_r^{(g)})$ (solid green line), plotted as a function of the reproduction number $\mathcal{R}_r^{(g)}$. Dashed, horizontal gray line corresponds to a selection gradient $s'_{\mathcal{R}_r^{(g)}}(\mathcal{R}_r^{(g)}) = 0$, and the vertical dashed black lines describe, from left to right, the ESS sociality strategy $\mathcal{R}_{\text{ESS1}} = \frac{1}{c} < 1$ featuring no long-time contagion, the evolutionarily-unstable strategy $\mathcal{R}_{\text{uns}} > 1$, and the ESS sociality strategy $\mathcal{R}_{\text{ESS2}} > \mathcal{R}_{\text{uns}}$ under which both contagions survive at equilibrium. Because selection gradient is negative for some sociality strategies $\mathcal{R}_r^{(g)} > 1$, there is a regime of strategies in which the good contagion is eliminated under the adaptive dynamics. The substitution value for is $\rho = 0.5$ for both panels, and the weight parameter is $\alpha = 0.525$ in the right panel.

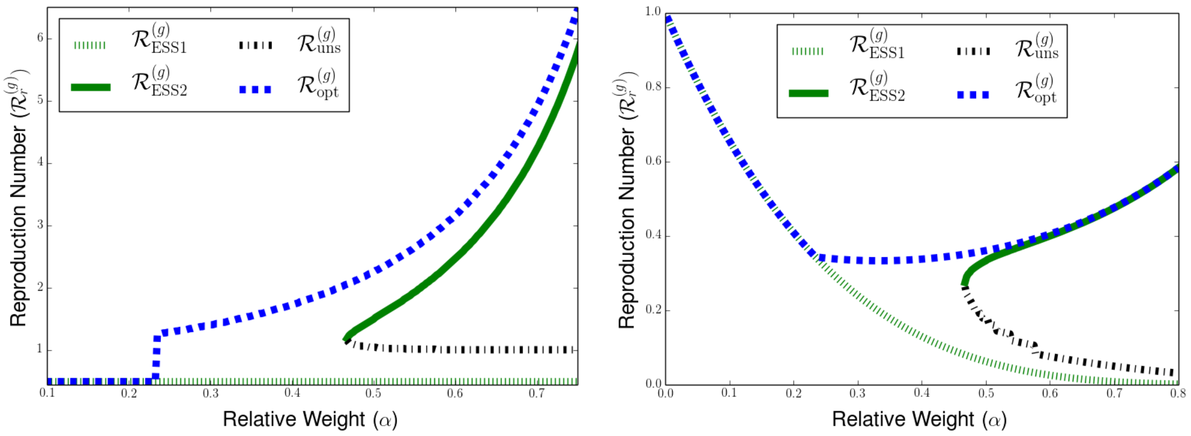

Figure 7: Illustration of the social dilemma for adaptive dynamics when the bad contagion spreads more readily than the good contagion ($c = 2$). Reproduction numbers $\mathcal{R}_r^{(g)}$ for sociality strategies (left panel) and associated social utilities $U[\mathcal{R}_r^{(g)}, \mathcal{R}_r^{(g)}]$ are plotted as functions of the relative weight parameter α for the CES utility. Strategies presented are the socially-optimal sociality strategy $\mathcal{R}_{\text{opt}}^{(g)}$ (dashed blue line), the evolutionarily-stable strategy $\mathcal{R}_{\text{ESS1}} = \frac{1}{c}$ at which no contagion spreads (dotted green line), the evolutionarily-stable strategy $\mathcal{R}_{\text{ESS2}}$ at which both contagions spread (solid green line), and the evolutionarily-unstable singular strategy $\mathcal{R}_{\text{uns}}$ separating the basins of attraction of the two ESS sociality strategy (dash-dotted black line). CES utility has weight parameters ranging from $\alpha = 0$ to $\alpha = 0.8$, and the substitution parameter is $\rho = 0.25$ for both panels.

B.6 Assortative Interactions and Mitigating the Social Dilemma

Now we consider a modified version of our processes in which individuals have a disproportionate probability of interaction with individuals who use the same sociality strategy. This mechanism, often called preferential mixing, has been used to study disease dynamics in multigroup settings [18]. Similar kinds of assortative interactions have been shown to help promote cooperation in two-strategy games [19–21], and also help to promote more efficient evolutionarily-stable strategies for continuous-strategy social dilemmas [21–24].

For our model of assortative interactions, we again characterize the interaction rate of individuals through their rate of social interaction σ and their corresponding reproduction number $\mathcal{R}^{(g)} := \frac{\sigma p_g}{\gamma_g}$ under the good contagion with well-mixed interactions. For each interaction, we assume that an individual is automatically paired with someone following the same sociality strategy with probability ρ, and is paired with a randomly chosen member of the group with probability $1 - \rho$. The parameter ρ measures the degree of homophily of social interactions, with $\rho = 0$ corresponding well-mixed interactions and $\rho = 1$ corresponding to the monomorphic interaction case.

In a population composed of a fraction f following a mutant socaility strategy $\mathcal{R}_m^{(g)}$ and a fraction $1 - f$ following a resident strategy $\mathcal{R}_r^{(g)}$, we can use the approach from Section A.1 to derive the dimorphic dynamics for both the good and bad contagion with assortative interactions. For the

good contagion, we obtain the following systems of differential equations

$$\begin{aligned}\frac{1}{\gamma_g}\frac{\mathrm{d}I_r^{(g)}}{\mathrm{d}t} &= \mathcal{R}_r^{(g)}\Big[\rho I_r^{(g)} + (1-\rho)\frac{\mathcal{R}_r^{(g)}(1-f)I_r^{(g)} + \mathcal{R}_m^{(g)} f I_m^{(g)}}{\mathcal{R}_r^{(g)}(1-f) + \mathcal{R}_m^{(g)} f}\Big]\left(1 - I_r^{(g)}\right) - I_r^{(g)} \\ \frac{1}{\gamma_g}\frac{\mathrm{d}I_m^{(g)}}{\mathrm{d}t} &= \mathcal{R}_m^{(g)}\Big[\rho I_m^{(g)} + (1-\rho)\frac{\mathcal{R}_r^{(g)}(1-f)I_r^{(g)} + \mathcal{R}_m^{(g)} f I_m^{(g)}}{\mathcal{R}_r^{(g)}(1-f) + \mathcal{R}_m^{(g)} f}\Big]\left(1 - I_m^{(g)}\right) - I_m^{(g)}\end{aligned}. \tag{S1.B.25}$$

We can also derive an analogous system for the bad contagion with reproduction numbers $\mathcal{R}_m^{(b)} = c\mathcal{R}_m^{(g)}$ and $\mathcal{R}_r^{(b)} = c\mathcal{R}_r^{(g)}$. In the adaptive dynamics limit in which $f \to 0$, the residents see no effect from interactions with mutants and the contagion dynamics for the resident type reduces to the monomorphic dynamics of Equation (1). Therefore the equilibrium levels for the resident strategy are given by $\hat{I}_r^{(g)} = 1 - \frac{1}{\mathcal{R}_r^{(g)}}$ and $\hat{S}_r^{(b)} = \frac{1}{c\mathcal{R}_r^{(g)}}$. In the same limit, the contagion dynamics for the mutant population are governed by

$$\frac{1}{\gamma_g}\frac{\mathrm{d}I_m^{(g)}}{\mathrm{d}t} = \mathcal{R}_m^{(g)}\left(1 - I_m^{(g)}\right)\left(\rho I_m^{(g)} + (1-\rho)I_r^{(g)}\right) - I_m^{(g)} \tag{S1.B.26a}$$

$$\frac{1}{\gamma_b}\frac{\mathrm{d}I_m^{(b)}}{\mathrm{d}t} = c\mathcal{R}_m^{(g)}\left(1 - I_m^{(b)}\right)\left(\rho I_m^{(b)} + (1-\rho)I_r^{(b)}\right) - I_m^{(b)}. \tag{S1.B.26b}$$

For simplicity, we will restrict our attention for the adaptive dynamics analysis to the case of $\mathcal{R}_m^{(g)} > \dfrac{1}{c}$, so that the resident dynamics will approach an endemic equilibrium for both the good and bad contagion. Using this assumption and the fact that the resident population evolves independently to its stable equilibrium, we can substitute $\hat{I}_r^{(g)} = 1 - \frac{1}{\mathcal{R}_r^{(g)}}$ into Equation (S1.B.26) to see that the long-time behavior of the mutant population under the good contagion will be determined by

$$\begin{aligned}\frac{1}{\gamma_g}\frac{\mathrm{d}I_m^{(g)}}{\mathrm{d}t} &= -\rho\mathcal{R}_m^{(g)}(I_m^{(g)})^2 + \left(\rho\mathcal{R}_m^{(g)} - (1-\rho)\mathcal{R}_m^{(g)}\hat{I}_r^{(g)} - 1\right)I_m^{(g)} + (1-\rho)\mathcal{R}_m^{(g)}\hat{I}_r^{(g)} \\ &= -\rho\mathcal{R}_m^{(g)}(I_m^{(g)})^2 - \left(1 + \mathcal{R}_m^{(g)} - 2\rho\mathcal{R}_m^{(g)} - (1-\rho)\frac{\mathcal{R}_m^{(g)}}{\mathcal{R}_r^{(g)}}\right)I_m^{(g)} + \mathcal{R}_m^{(g)}(1-\rho)\left(1 - \frac{1}{\mathcal{R}_r^{(g)}}\right).\end{aligned} \tag{S1.B.27}$$

Then we see that the only biologically feasible equilibrium for the mutant population under the good contagion is

$$\begin{aligned}\hat{I}_m^{(g)} = &-\frac{1 + \mathcal{R}_m^{(g)} - 2\rho\mathcal{R}_m^{(g)} - (1-\rho)\frac{\mathcal{R}_m^{(g)}}{\mathcal{R}_r^{(g)}}}{2\rho\mathcal{R}_m^{(g)}} \\ &+ \frac{\sqrt{\left(1 + \mathcal{R}_m^{(g)} - 2\rho\mathcal{R}_m^{(g)} - (1-\rho)\frac{\mathcal{R}_m^{(g)}}{\mathcal{R}_r^{(g)}}\right)^2 - 4\rho(1-\rho)\left(\mathcal{R}_m^{(g)}\right)^2\left(1 - \frac{1}{\mathcal{R}_r^{(g)}}\right)}}{2\rho\mathcal{R}_m^{(g)}}.\end{aligned} \tag{S1.B.28}$$

By a similar approach, the mutant population will achieve an equilibrium for the bad contagion of

$$\begin{aligned}\hat{I}_m^{(g)} = &-\frac{1 + c\mathcal{R}_m^{(g)} - 2\rho c\mathcal{R}_m^{(g)} - (1-\rho)\frac{\mathcal{R}_m^{(g)}}{\mathcal{R}_r^{(g)}}}{2\rho c\mathcal{R}_m^{(g)}} \\ &+ \frac{\sqrt{\left(1 + c\mathcal{R}_m^{(g)} - 2\rho c\mathcal{R}_m^{(g)} - (1-\rho)\frac{\mathcal{R}_m^{(g)}}{\mathcal{R}_r^{(g)}}\right)^2 - 4\rho(1-\rho)c^2\left(\mathcal{R}_m^{(g)}\right)^2\left(1 - \frac{1}{c\mathcal{R}_r^{(g)}}\right)}}{2\rho c\mathcal{R}_m^{(g)}}.\end{aligned} \tag{S1.B.29}$$

Using these equilibrium levels of infectiousness for the mutant population, we can calculate the local selection gradient for the Cobb-Douglas utility. The tedious calculation included in the supplemental Mathematica notebook (available at https://github.com/dbcooney/Social-Dilemmas-of-Sociality-due-to-Beneficial-and-Costly-Contagion) eventually yields the local selection gradient

$$s'_{\mathcal{R}_m^{(g)}}(\mathcal{R}_m^{(g)}) = \frac{-(1-\alpha)c(\mathcal{R}_m^{(g)})^2 + (1-\alpha+c\left[\alpha+\rho\left(1-\alpha\right)\right])\,\mathcal{R}_m^{(g)} - \rho}{R\left(R-\rho\right)\left(cR-\rho\right)} \tag{S1.B.30}$$

Notably, we see that the selection gradient coincides with the non-assortative case when $\rho = 0$ and with the derivative of the monomorphic utility function when $\rho = 1$. The numerator of the selection gradient from Equation (S1.B.30) is a decreasing function of $\mathcal{R}_m^{(g)}$, and therefore, for each assortment probability ρ, there will be a unique evolutionarily-stable sociality strategy $\mathcal{R}_{\text{ESS}}^{(g)}(\rho)$. Setting the lefthand side of Equation (S1.B.30) equal to 0, we see that this unique family of ESSes is given by

$$\mathcal{R}_{\text{ESS}}^{(g)}(\rho) = \frac{1+c\left(1-\alpha\right)+\rho c(1-\alpha)+\sqrt{(\alpha-\alpha c+\alpha c\rho-c\rho-1)^2-4\rho c\left(1-\alpha\right)}}{2c\left(1-\alpha\right)}. \tag{S1.B.31}$$

We can also visualize how the evolutionarily-stable level of sociality varies with ρ by plotting the sign of selection gradient for varying values of ρ and $\mathcal{R}_m^{(g)}$ [25]. In Figure 8, we plot the regions of positive selection gradient in red and of negative selection gradient in blue for examples corresponding to both $c = \frac{1}{2}$ (left) and $c = 2$ (right). The border of the red and blue regions is given by the solid black line the two regions given by the expression from Equation (S1.B.31) for the evolutionarily-stable strategy $\mathcal{R}_{\text{ESS}}^{(g)}(\rho)$ for given ρ. As a point of comparison, we plot the black dashed line which interpolates linearly in r between the evolutionarily-stable sociality $\mathcal{R}_{\text{ESS}}^{(g)}$ when assortment $\rho = 0$ and the socially-optimal level of sociality $\mathcal{R}_{\text{opt}}^{(g)}$ when $\rho = 1$. We see that, in both cases, $\mathcal{R}_{\text{ESS}}^{(g)}(\rho)$ moves towards the social optimum more slowly than this linear interpolation, which tells us that, while the addition of assortment does help to mitigate the social dilemma, it takes a relatively large level of assortment to make an appreciable impact on improving the social efficiency of the evolutionarily-stable outcome.

C Microfoundation for Population Utility Function

So far, we have consider the utility functions for our evolutionary dynamics as given functions of the equilibrium levels of the contagion in the population. Here we offer a derivation of how linear and Cobb-Douglas utility functions can arise as the expected or time-averaged utility for an individual living in a population at steady state under the contagion dynamics. This serves as a means for understanding how different tradeoffs between the benefits of the good contagion and the costs of the bad contagion at the population level are connected to the underlying payoffs achieved by individuals following various sociality strategies in the presence of the coupled contagion dynamics.

We calculate the expected utility from payoffs associated with an individual's state under the good and bad contagion, treating the fractions susceptible and infectious at steady state as the probabilities that a given individual is in that state. We describe the status of individuals for two contagion by the pair of types $T = (T^g, T^b) \in \{S^{(g)}, I^{(g)}\} \times \{S^{(b)}, I^{(g)}\}$. For the good contagion, we assume that a contribution of $a_g > 0$ is made to individual utility when an individual is infected, while a contribution of 0 is made when the individual is susceptible. For the bad contagion, we assume that a contribution of 0 is made to individual utility when the individual is infected, while a

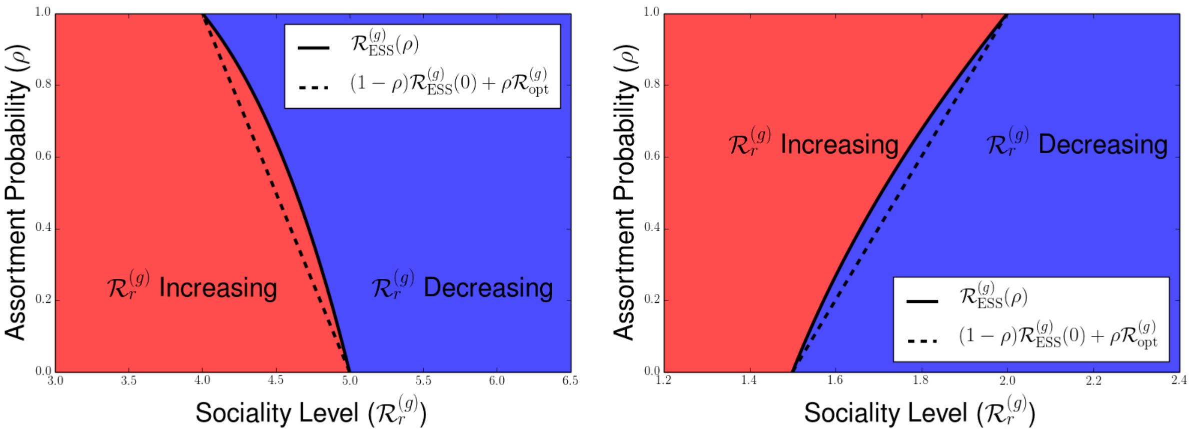

Figure 8: Plot of regions in which the local selection gradient is increasing (red) and decreasing (blue) for varying values of sociality strategy $\mathcal{R}^{(g)}_m$ and assortment probability ρ, with $c = \frac{1}{2}$ (left) and $c = 2$ (right). Solid line black line is the evolutionarily-stable level of sociality $\mathcal{R}^{(g)}_{\rm ESS}(\rho)$ for assortment probability r, and the dashed black line corresponds to $(1-\rho)\mathcal{R}^{(g)}_{\rm ESS} + \rho\mathcal{R}^{(g)}_{\rm opt}$, linearly interpolating between the ESS under well-mixed interactions and the socially-optimal level of sociality.

contribution of $a_b > 0$ is made when the individual is susceptible. If we consider that an individual's utility is the sum of its contributions from the good and bad contagion, then we can see that the utility is given for each of four possible disease states as

$$\begin{array}{c c} & \begin{array}{cc} S^{(g)} & I^{(g)} \end{array} \\ \begin{array}{c} S^{(b)} \\ I^{(b)} \end{array} & \left[\begin{array}{cc} a_b & a_b + a_g \\ 0 & a_g \end{array}\right] \end{array} \tag{S1.C.1}$$

Because the two contagions spread independently through the population, the average utility of a population with monomorphic interaction rates are given by

$$\begin{aligned} u(I^{(g)}, S^{(b)}) &= S^{(b)}\left(1 - I^{(g)}\right) a_b + S^{(b)} I^{(g)} \left(a_b + a_g\right) \\ &+ \left(1 - S^{(b)}\right)\left(1 - I^{(g)}\right)(0) + \left(1 - S^{(b)}\right) I^{(g)} a_g \\ &= a_b S^{(b)} + a_g I^{(g)}, \end{aligned} \tag{S1.C.2}$$

and choosing parameters $a_g = \alpha \in [0,1]$ and $a_b = 1 - \alpha \in [0,1]$, we recover the linear utility function for a monomorphic population

$$U(I^{(g)}, S^{(b)}) = \alpha I^{(g)} + (1-\alpha) S^{(b)} \tag{S1.C.3}$$

If instead, we consider individual utility given by the product of the contributions from the good contagion and the bad contagion, the utility for the four pairs of disease states is given by

$$\begin{array}{cc} & \begin{array}{cc} S^{(g)} & I^{(g)} \end{array} \\ \begin{array}{c} S^{(b)} \\ I^{(b)} \end{array} & \begin{bmatrix} 0 & a_b a_g \\ 0 & 0 \end{bmatrix} \end{array} \tag{S1.C.4}$$

Because the two contagion spread independently, the average utility in the population is given by

$$u(I^{(g)}, S^{(b)}) = a_b a_g I^{(g)} S^{(b)},$$

and the choice $a_b = a_g = 1$ provides us with the utility function takes the form

$$U(I^{(g)}, S^{(b)}) = I^{(g)} S^{(b)}$$

corresponding to a Cobb-Douglas utility with positive returns to scale and symmetric dependence on its inputs.

In a similar manner, we can derive Cobb-Douglas utilities with asymmetric weight placed on $I^{(g)}$ and $S^{(b)}$ by considering the possibility that the utility of an individual itself depends upon social interactions taking place at the equilibrium contagion frequencies. For example, we can obtain a utility of the form $U(I^{(g)}, S^{(b)}) = (I^{(g)})^2 S^{(b)}$ under a process in which individuals are paired with a random partner, and an individual only receives a positive payoff if they are susceptible to the bad contagion and both they and their partner are infected by the good contagion. Analogously, we can derive a utility of the form $U(I^{(g)}, S^{(b)}) = I^{(g)} \left(S^{(b)}\right)^2$ in a scenario in which an individual only obtains positive payoff if they are infected with the good contagion and if they and a randomly chosen partner are both susceptible to the bad contagion.

By considering more complex matching processes and rules for assigning positive payoff, similar Cobb-Douglas utilities can be derived, highlighting the different ways in which the enjoyment of access to beneficial contagions and absence of harmful contagions can depend on the state of the whole population. While our underlying contagion processes are simple SIS contagions, the presence of utility functions of this form means that the evolutionary dynamics can depend on the population state in a manner reminiscent of complex contagion process and of models of evolutionary games with imitation protocols resembling complex contagion [26].

References

[1] O. Diekmann, J. A. P. Heesterbeek, and J. A. Metz, "On the definition and the computation of the basic reproduction ratio $\mathcal{R}_0$ in models for infectious diseases in heterogeneous populations," *Journal of Mathematical Biology*, vol. 28, no. 4, pp. 365–382, 1990.

[2] P. van den Driessche and J. Watmough, "Reproduction numbers and sub-threshold endemic equilibria for compartmental models of disease transmission," *Mathematical Biosciences*, vol. 180, no. 1-2, pp. 29–48, 2002.

[3] J. Yorke, H. Hethcote, and A. Nold, "Dynamics and control of the transmission of gonorrhea," *Sexually Transmitted Diseases*, vol. 5, no. 2, p. 51, 1978.

[4] R. M. May and R. M. Anderson, "The transmission dynamics of human immunodeficiency virus (HIV)," *Philosophical Transactions of the Royal Society B: Biological Sciences*, vol. 321, no. 1207, pp. 565–607, 1988.

[5] A. Scala, L. N. Amaral, and M. Barthélémy, "Small-world networks and the conformation space of a short lattice polymer chain," *EPL (Europhysics Letters)*, vol. 55, no. 4, p. 594, 2001.

[6] A. L. Lloyd and R. M. May, "How viruses spread among computers and people," *Science*, vol. 292, no. 5520, pp. 1316–1317, 2001.

[7] R. Pastor-Satorras and A. Vespignani, "Immunization of complex networks," *Physical Review E*, vol. 65, no. 3, p. 036104, 2002.

[8] R. Pastor-Satorras and A. Vespignani, "Epidemic dynamics in finite size scale-free networks," *Physical Review E*, vol. 65, no. 3, p. 035108, 2002.

[9] E. Koutsoupias and C. Papadimitriou, "Worst-case equilibria," in *Annual Symposium on Theoretical Aspects of Computer Science*, pp. 404–413, Springer, 1999.

[10] C. Papadimitriou, "Algorithms, games, and the internet," in *Proceedings of the Thirty-Third Annual ACM Symposium on Theory of Computing*, pp. 749–753, 2001.

[11] G. Christodoulou and E. Koutsoupias, "The price of anarchy of finite congestion games," in *Proceedings of the Thirty-Seventh Annual ACM Symposium on Theory of Computing*, pp. 67–73, 2005.

[12] R. Carmona, C. V. Graves, and Z. Tan, "Price of anarchy for mean field games," *ESAIM: Proceedings and Surveys*, vol. 65, pp. 349–383, 2019.

[13] S. A. Geritz, É. Kisdi, G. Meszéna, and J. A. Metz, "Evolutionarily singular strategies and the adaptive growth and branching of the evolutionary tree," *Evolutionary Ecology*, vol. 12, no. 1, pp. 35–57, 1998.

[14] O. Diekmann, "A beginners guide to adaptive dynamics," *Summer School on Mathematical Biology*, pp. 63–100, 2002.

[15] Å. Brännström, J. Johansson, and N. Von Festenberg, "The hitchhiker's guide to adaptive dynamics," *Games*, vol. 4, no. 3, pp. 304–328, 2013.

[16] K. J. Arrow, H. B. Chenery, B. S. Minhas, and R. M. Solow, "Capital-labor substitution and economic efficiency," *The Review of Economics and Statistics*, vol. 43, no. 3, pp. 225–250, 1961.

[17] A. Mas-Colell, M. D. Whinston, and J. R. Green, *Microeconomic Theory*, vol. 1. Oxford University Press New York, 1995.

[18] H. W. Hethcote and J. W. Van Ark, "Epidemiological models for heterogeneous populations: proportionate mixing, parameter estimation, and immunization programs," *Mathematical Biosciences*, vol. 84, no. 1, pp. 85–118, 1987.

[19] A. Grafen, "The hawk-dove game played between relatives," *Animal Behaviour*, vol. 27, pp. 905–907, 1979.

[20] T. C. Bergstrom, "The algebra of assortative encounters and the evolution of cooperation," *International Game Theory Review*, vol. 5, no. 03, pp. 211–228, 2003.

[21] M. van Veelen, B. Allen, M. Hoffman, B. Simon, and C. Veller, "Hamilton's rule," *Journal of Theoretical Biology*, vol. 414, pp. 176–230, 2017.

[22] D. M. Cornforth, D. J. Sumpter, S. P. Brown, and Å. Brännström, "Synergy and group size in microbial cooperation," *The American Naturalist*, vol. 180, no. 3, pp. 296–305, 2012.

[23] K. Coder Gylling and Å. Brännström, "Effects of relatedness on the evolution of cooperation in nonlinear public goods games," *Games*, vol. 9, no. 4, p. 87, 2018.

[24] S. Iyer and T. Killingback, "Evolution of cooperation in social dilemmas with assortative interactions," *Games*, vol. 11, no. 4, p. 41, 2020.

[25] C. M. Saad-Roy, N. S. Wingreen, S. A. Levin, and B. T. Grenfell, "Dynamics in a simple evolutionary-epidemiological model for the evolution of an initial asymptomatic infection stage," *Proceedings of the National Academy of Sciences*, 2020.

[26] V. V. Vasconcelos, S. A. Levin, and F. L. Pinheiro, "Consensus and polarization in competing complex contagion processes," *Journal of the Royal Society Interface*, vol. 16, no. 155, p. 20190196, 2019.
