# Supplementary material for: Social dilemmas of sociality due to beneficial and costly contagion
Source: PLoS Comput Biol. 2022 Nov 21;18(11):e1010670. doi: 10.1371/journal.pcbi.1010670 (PMC9721494; doi:10.1371/journal.pcbi.1010670)

In this notebook, we calculate the local selection gradient for our model with assortative interactions studied in Section B.3 in the appendix. These calculations are used to produce Equation (B.14), which, in turn, is used to calculate the evolutionarily stable sociality strategy for a given assortment probability ρ.

First, we introduce the equilibrium level of contagion I_M for the rare mutant population with sociality strategy corresponding to reproduction number M for the good contagion, given the resident level of sociality strategy with reproduction number R for the good contagion. This expression was calculated in the adaptive dynamics limit for the good contagion in Equation (B.12) and for the bad contagion in Equation (B.13).

```
ImMinus[M_, R_, ρ_] :=
```

$$\frac{1}{2 M \rho}\left(-1 - M + 2 M \rho - \sqrt{\left(-1 - M + 2 M \rho + \frac{M}{R} - \frac{M \rho}{R}\right)^2 + 4 M \rho \left(M - M \rho - \frac{M}{R} + \frac{M \rho}{R}\right)} + \frac{M}{R} - \frac{M \rho}{R}\right)$$

Next, we compute the Cobb-Douglas utility for the mutant population given the equilibrium levels of the good and bad contagion.

```
Utility[M_, R_, ρ_, α_, c_] :=
 α Log[ImMinus[M, R, ρ]] + (1 - α) Log[1 - ImMinus[c M, c R, ρ]]
```

To calculate the local selection gradient, we first differentiate the utility for mutant individuals with respect to the mutant reproduction number M, given a fixed resident reproduction number R.

```
D[Utility[M, R, ρ, α, c], M]
```

$$\left(2 M \alpha \rho \left(\frac{1}{2 M \rho}\left(1 - \frac{1}{R} - 2\rho + \frac{\rho}{R} - \left(4 M \rho \left(1 - \frac{1}{R} - \rho + \frac{\rho}{R}\right) + 2\left(-1 + \frac{1}{R} + 2\rho - \frac{\rho}{R}\right)\left(-1 - M + \frac{M}{R} + 2 M \rho - \frac{M \rho}{R}\right) + 4\rho\left(M - \frac{M}{R} - M\rho + \frac{M\rho}{R}\right)\right) \Big/ \left(2\sqrt{\left(\left(-1 - M + \frac{M}{R} + 2 M \rho - \frac{M\rho}{R}\right)^2 + 4 M \rho\left(M - \frac{M}{R} - M\rho + \frac{M\rho}{R}\right)\right)}\right)\right) - \frac{1}{2 M^2 \rho}\left(-1 + M - \frac{M}{R} - 2 M \rho + \frac{M\rho}{R} - \sqrt{\left(\left(-1 - M + \frac{M}{R} + 2 M \rho - \frac{M\rho}{R}\right)^2 + 4 M \rho\left(M - \frac{M}{R} - M\rho + \frac{M\rho}{R}\right)\right)}\right)\right)\right) \Big/ \left(-1 + M - \frac{M}{R} - 2 M \rho + \frac{M\rho}{R} - \sqrt{\left(-1 - M + \frac{M}{R} + 2 M \rho - \frac{M\rho}{R}\right)^2 + 4 M \rho\left(M - \frac{M}{R} - M\rho + \frac{M\rho}{R}\right)}\right) +$$

$$\left((1-\alpha)\left(-\frac{1}{2 c M \rho}\left(c - \frac{1}{R} - 2 c \rho + \frac{\rho}{R} - \left(4 c M \rho\left(c - \frac{1}{R} - c\rho + \frac{\rho}{R}\right) + 2\left(-c + \frac{1}{R} + 2 c \rho - \frac{\rho}{R}\right)\left(-1 - c M + \frac{M}{R} + 2 c M \rho - \frac{M\rho}{R}\right) + 4 c \rho\left(c M - \frac{M}{R} - c M \rho + \frac{M\rho}{R}\right)\right) \Big/ \left(2\sqrt{\left(\left(-1 - c M + \frac{M}{R} + 2 c M \rho - \frac{M\rho}{R}\right)^2 + 4 c M \rho\left(c M - \frac{M}{R} - c M \rho + \frac{M\rho}{R}\right)\right)}\right)\right) + \frac{1}{2 c M^2 \rho}\left(-1 + c M - \frac{M}{R} - 2 c M \rho + \frac{M\rho}{R} - \sqrt{\left(\left(-1 - c M + \frac{M}{R} + 2 c M \rho - \frac{M\rho}{R}\right)^2 + 4 c M \rho\left(c M - \frac{M}{R} - c M \rho + \frac{M\rho}{R}\right)\right)}\right)\right)\right) \Big/ \left(1 - \frac{1}{2 c M \rho}\left(-1 + c M - \frac{M}{R} - 2 c M \rho + \frac{M\rho}{R} - \sqrt{\left(\left(-1 - c M + \frac{M}{R} + 2 c M \rho - \frac{M\rho}{R}\right)^2 + 4 c M \rho\left(c M - \frac{M}{R} - c M \rho + \frac{M\rho}{R}\right)\right)}\right)\right)$$

To further calculate the local selection gradient, we evaluate the derivative of the mutant utility with respect to mutant reproduction number M for the case in which the mutant and resident reproduction numbers coincide (M = R). This allows us to see whether utility will increase or decrease for a mutant with sociality strategy near the resident sociality strategy.

```
D[Utility[M, R, ρ, α, c], M] /. {M → R} // FullSimplify
```

$$\frac{1}{R}\left(-\frac{\alpha\left(R+\sqrt{(R-\rho)^2}+\rho-2R\rho\right)}{\sqrt{(R-\rho)^2}\left(2+\sqrt{(R-\rho)^2}-\rho+R\left(-1+2\rho\right)\right)}+\frac{(-1+\alpha)\left(\rho+\sqrt{(-cR+\rho)^2}+c\left(R-2R\rho\right)\right)}{\sqrt{(-cR+\rho)^2}\left(2-\rho+\sqrt{(-cR+\rho)^2}+cR\left(-1+4\rho\right)\right)}\right)$$

We can simplify the expression for the local selection gradient using the fact that reproduction numbers for the good contagion always satisfy R >= 1. In addition, we will assume that cR > ρ, which simplifies our expression. This second assumption is justified if we restrict attention to cases in which the socially optimal sociality strategy is achieved at a critical point of the monomorphic utility function (i.e. R_{opt} > 1 / c, which occurs when c > 1 - α).

```
FullSimplify[
```

$$-\frac{1}{2R}\left(\frac{-2+cR+\rho+\sqrt{(-cR+\rho)^2}}{\sqrt{(-cR+\rho)^2}}+\alpha\left(-1-\frac{2}{\sqrt{(R-\rho)^2}}+\frac{2}{\sqrt{(-cR+\rho)^2}}-\frac{cR+\rho}{\sqrt{(-cR+\rho)^2}}\right)\right),$$

```
{R - ρ > 0, c R - ρ > 0}]
```

$$\frac{\frac{\alpha}{R-\rho}+\frac{(-1+cR)(-1+\alpha)}{cR-\rho}}{R}$$

$$\frac{\frac{\alpha}{R-\rho}+\frac{(-1+cR)(-1+\alpha)}{cR-\rho}}{R}$$

In the next two steps, we simplify the expression for the local selection gradient to obtain the formula presented in Equation (B.14) in the appendix.

$$\text{Together}\left[\frac{\frac{\alpha}{R-\rho}+\frac{(-1+cR)(-1+\alpha)}{cR-\rho}}{R}\right]$$

$$\frac{R-cR^2-R\alpha+cR\alpha+cR^2\alpha-\rho+cR\rho-cR\alpha\rho}{R\left(R-\rho\right)\left(cR-\rho\right)}$$

```
Collect[R - c R² - R α + c R α + c R² α - ρ + c R ρ - c R α ρ, R] / (R (R - ρ) (c R - ρ))
```

$$\frac{R^2\left(-c+c\alpha\right)-\rho+R\left(1-\alpha+c\alpha+c\rho-c\alpha\rho\right)}{R\left(R-\rho\right)\left(cR-\rho\right)}$$

Next, we set the selection gradient equal to zero to calculate the evolutionarily stable sociality strategy for a given assortment probability ρ. We can use the fact that the numerator of the selection gradient is

a decreasing function of R to see that the positive root (listed second) is the unique ESS.

$$\text{Solve}\left[\frac{\frac{\alpha}{R-\rho}+\frac{(-1+c\,R)\,(-1+\alpha)}{c\,R-\rho}}{R}==0,\,R\right]$$

$$\left\{\left\{R\to\frac{1}{2\,(-c+c\,\alpha)}\left(-1+\alpha-c\,\alpha-c\,\rho+c\,\alpha\,\rho-\sqrt{4\,(-c+c\,\alpha)\,\rho+(1-\alpha+c\,\alpha+c\,\rho-c\,\alpha\,\rho)^2}\right)\right\},\right.$$
$$\left.\left\{R\to\frac{1}{2\,(-c+c\,\alpha)}\left(-1+\alpha-c\,\alpha-c\,\rho+c\,\alpha\,\rho+\sqrt{4\,(-c+c\,\alpha)\,\rho+(1-\alpha+c\,\alpha+c\,\rho-c\,\alpha\,\rho)^2}\right)\right\}\right\}$$

Introducing shorthand to calculate the regions in which the local selection gradient is positive or negative (given assortment probability ρ).

$$G[R\_,\,p\_,\,c\_,\,\alpha\_]:=$$
$$-\frac{1}{2\,R}\left(\frac{-2+c\,R+\rho+\sqrt{(-c\,R+\rho)^2}}{\sqrt{(-c\,R+\rho)^2}}+\alpha\left(-1-\frac{2}{\sqrt{(R-\rho)^2}}+\frac{2}{\sqrt{(-c\,R+\rho)^2}}-\frac{c\,R+\rho}{\sqrt{(-c\,R+\rho)^2}}\right)\right)$$

Plot of regions in which local selection gradient is increasing (filled in blue) and decreasing (filled in orange) depending on the resident sociality reproduction number R and the assortment probability ρ. The boundary between the regions corresponds to the ESS sociality strategy. In first plot, we consider case in which c = 1/2, so the ESS with no assortative interactions ($\rho = 0$) features more interactions than experienced by the socially optimal sociality strategy.

```
RegionPlot[{G[R, ρ, 1/2, 3/4] > 0 , G[R, ρ, 1/2, 3/4] < 0},
 {R, 1, 10}, {ρ, 0, 1}, PlotLegends → "Expressions",
 FrameLabel → { Style["Resident Sociality Strategy R", 16],
   Style["Assortment Probability ρ" , 16]}]
```

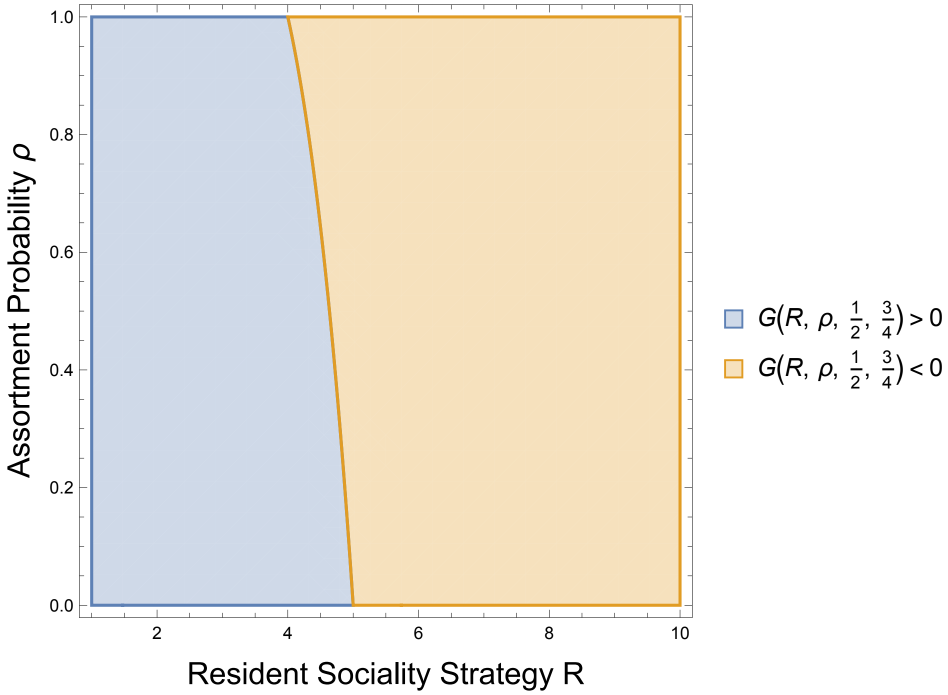

In second plot, we consider case in which c = 2, so the ESS with no assortative interactions ($\rho = 0$) features fewer interactions than experienced by the socially optimal sociality strategy.

```
RegionPlot[{G[R, ρ, 2, 3/4] > 0 , G[R, ρ, 2, 3/4] < 0},
 {R, 1, 8}, {ρ, 0, 1}, PlotLegends → "Expressions",
 FrameLabel → { Style["Resident Sociality Strategy R", 16],
   Style["Assortment Probability ρ" , 16]}]
```

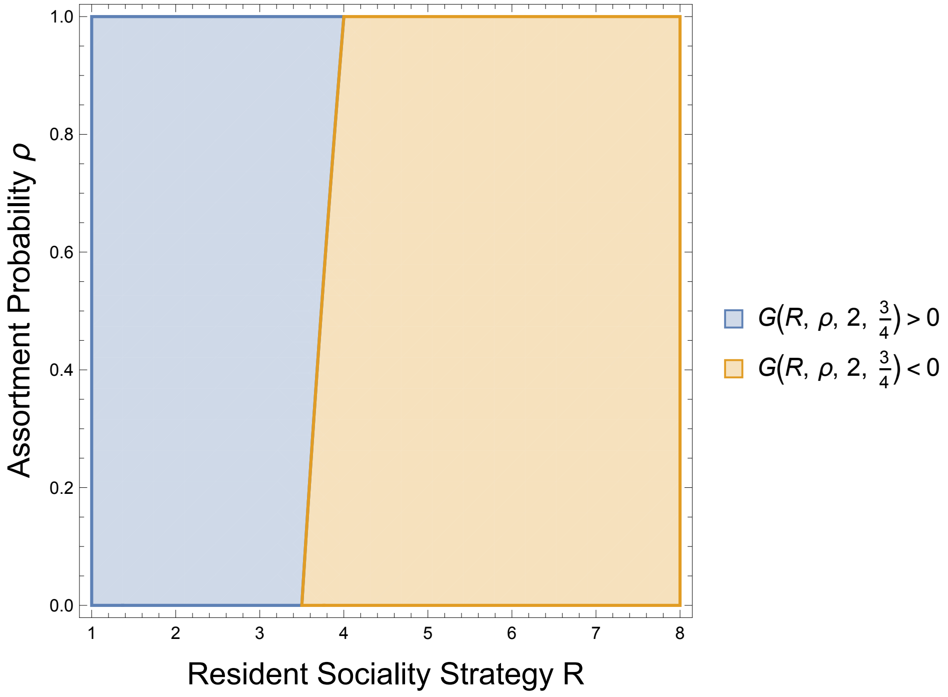

Supplement: S1 File — (PDF) [file pcbi.1010670.s002.pdf]
